# The human mitochondrial genome contains a second light strand promoter

**DOI:** 10.1016/j.molcel.2022.08.011

**Published:** 2022-08-30

**Authors:** Benedict G. Tan, Christian D. Mutti, Yonghong Shi, Xie Xie, Xuefeng Zhu, Pedro Silva-Pinheiro, Katja E. Menger, Héctor Díaz-Maldonado, Wei Wei, Thomas J. Nicholls, Patrick F. Chinnery, Michal Minczuk, Maria Falkenberg, Claes M. Gustafsson

**Affiliations:** 1 Department of Medical Biochemistry and Cell Biology, University of Gothenburg, Gothenburg 405 30, Sweden; 2 Medical Research Council Mitochondrial Biology Unit, University of Cambridge, Cambridge CB2 0XY, UK; 3 Wellcome Centre for Mitochondrial Research, Biosciences Institute, Newcastle University, Newcastle upon Tyne NE2 4HH, UK; 4 Department of Clinical Neurosciences, School of Clinical Medicine, University of Cambridge, Cambridge, UK

## Abstract

The human mitochondrial genome must be replicated and expressed in a timely manner to maintain energy metabolism and supply cells with adequate levels of adenosine triphosphate. Central to this process is the idea that replication primers and gene products both arise via transcription from a single light strand promoter (LSP) such that primer formation can influence gene expression, with no consensus as to how this is regulated. Here, we report the discovery of a second light strand promoter (LSP2) in humans, with features characteristic of a bona fide mitochondrial promoter. We propose that the position of LSP2 on the mitochondrial genome allows replication and gene expression to be orchestrated from two distinct sites, which expands our long-held understanding of mitochondrial gene expression in humans.

## Graphical abstract

**Graphical abstract:**
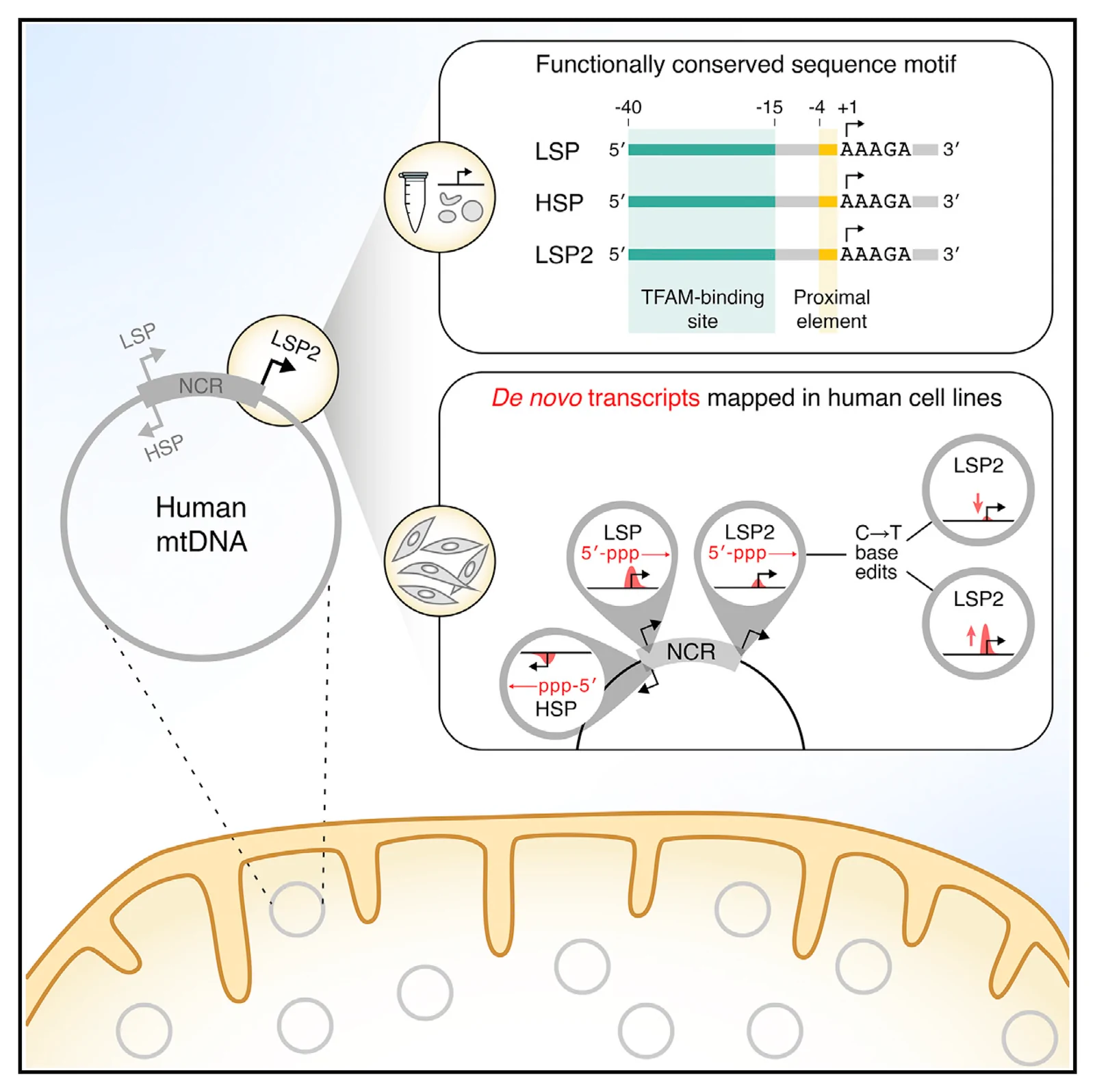

## Introduction

Nearly all eukaryotic cells rely on mitochondria to supply the majority of cellular adenosine triphosphate (ATP) through oxidative phosphorylation. A subset of proteins required for this process are uniquely encoded by mitochondrial DNA (mtDNA) (Gustafsson et al., 2016), which must be copied and expressed at appropriate levels to match the metabolic requirements of a cell.

**Figure 1:**
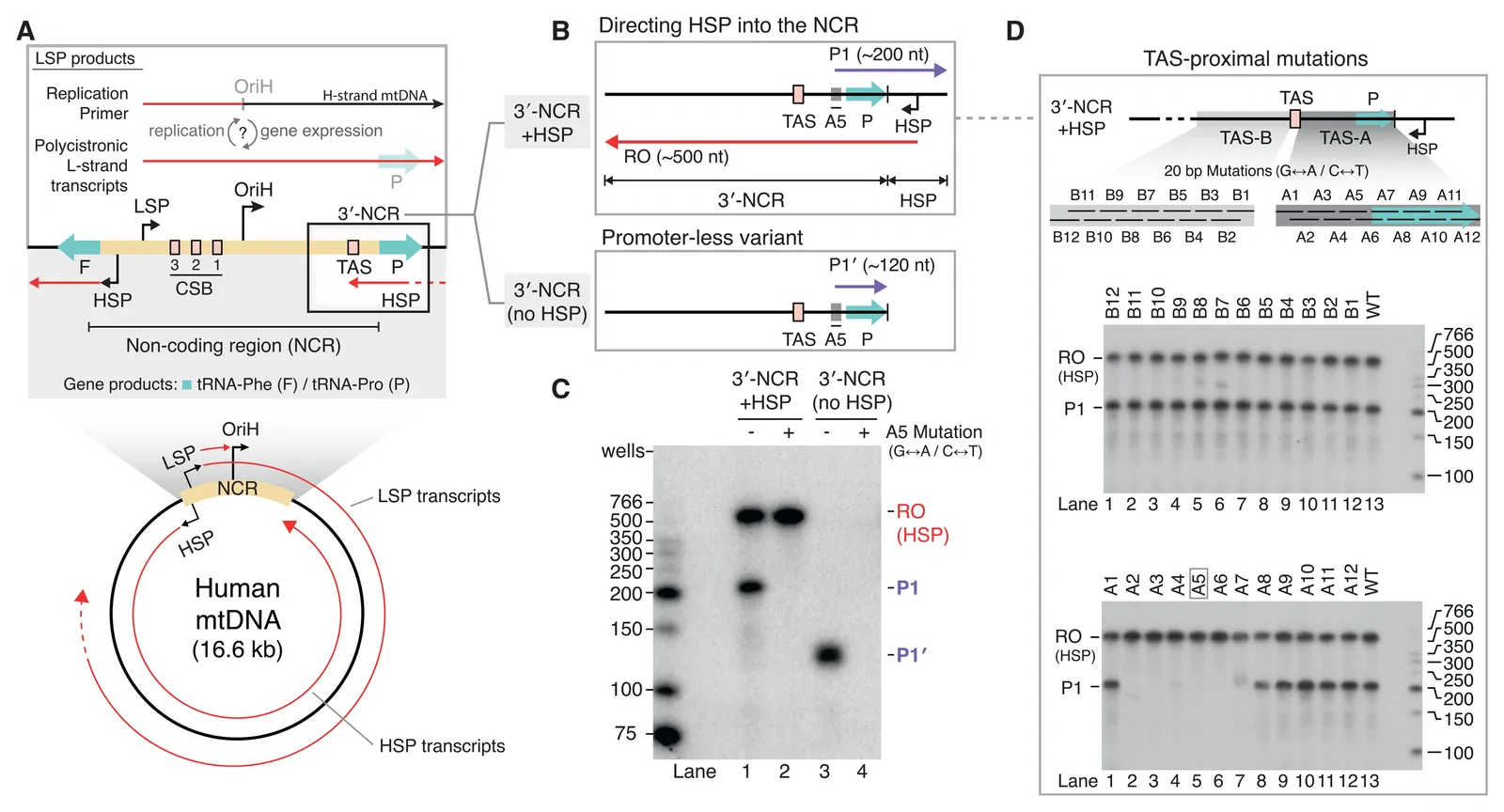
A locus at the 3′-end of the NCR is capable of initiating transcription (A) Simplified map of the human mitochondrial genome, with a magnified view of the NCR emphasizing two products of LSP. Red lines/arrows indicate RNA. A region at the 3′-end of the NCR (“3′-NCR”) was used to design templates in (B). (B) Schematic of DNA templates directing transcription from HSP into the 3′-NCR region. Red and purple arrows indicate potential transcription products found in (C) and (D). A locus proximal to TAS (“A5”, which was subjected to mutagenesis in (C), is highlighted in dark gray on each template. “RO” indicates run-off transcription products from HSP. (C) Products of *in vitro* transcription by POLRMT from the templates described in (B), separated on 8% urea-PAGE mini gels. (D) Mutagenesis of TAS-proximal sequences in template 3′-NCR+HSP, categorized into TAS-A and TAS-B and subdivided into 20 bp sub-regions with 10 bp overlaps, at which mutations were introduced (listed in Table S4). *In vitro* transcription products were separated on 8% urea-PAGE mini gels.

Human mtDNA consists of light (L) and heavy (H) strands. Each strand contains a promoter (LSP and HSP, respectively), which are located within a non-coding region (NCR) and were originally mapped by guanylyltransferase capping experiments in the early 1980’s (Montoya et al., 1982). LSP and HSP are located proximally to one another (~150 bp apart) close to the 5′-end of the NCR. This positions HSP immediately upstream of H-strand genes, whereas LSP is positioned upstream of the origin of H-strand mtDNA replication (OriH) and must transcribe through ~1 kb of the NCR before reaching the first L-strand gene (tRNA^Pro^, Figure 1A) (Gustafsson et al., 2016). Transcription in mitochondria is polycistronic, and transcripts synthesized from LSP and HSP are rapidly processed by the endoribonucleases RNase P and ELAC2 to release 5′-monophosphorylated mRNAs, tRNAs, and rRNAs for mitochondrial translation (Sanchez et al., 2011).

Transcription initiation from LSP and HSP requires the mitochondrial RNA polymerase (POLRMT) and two essential proteins, TFAM and TFB2M (Falkenberg et al., 2002; Shi et al., 2012). The binding of TFAM at the promoter region creates a sharp bend in the DNA and recruits POLRMT to the transcription start site (TSS) (Morozov et al., 2014). TFB2M binds to this complex and promotes the melting of the promoter DNA, enabling transcription initiation by POLRMT (Gaspari et al., 2004; Posse and Gustafsson, 2017; Sologub et al., 2009). A transcription elongation factor, TEFM, has also been identified that binds to POLRMT during translocation and promotes the formation of long transcription products both *in vitro* (Minczuk et al., 2011; Posse et al., 2015) and *in vivo* (Jiang et al., 2019). Apart from LSP and HSP, it has been proposed that a second H-strand promoter (HSP2) exists downstream of HSP, but its activity *in vivo* has remained controversial (Gustafsson et al., 2016; Litonin et al., 2010). This is notably because the TSSs of HSP2 mapped *in vitro* and in living cells are not in agreement (Montoya et al., 1982; Zollo et al., 2012) and because TFAM (a highly abundant protein component of mitochondrial nucleoids) represses transcription from HSP2 *in vitro* (Lodeiro et al., 2012; Zollo et al., 2012).

Mitochondria lack a dedicated primase that is typically used to synthesize short RNA primers at replication forks. This, along with the position of the mitochondrial promoters, has favored a model in which POLRMT instead uses LSP-derived transcripts to provide RNA primers for the initiation of mtDNA replication at the NCR (Figure 1A) (Pham et al., 2006; Wanrooij et al., 2010, 2012). Alternatively, if not precluded by primer formation, transcription from LSP can proceed beyond the NCR to supply L-strand genes. In this way, mtDNA replication and gene expression are seen as coupled events. The regulation of these two processes is not fully established, and the mechanism favoring L-strand gene expression over primer formation has been the subject of recent debate (Agaronyan et al., 2015; Falkenberg, 2018; Jiang et al., 2019).

In this work, we detail the unexpected discovery of a second light strand promoter (LSP2) found at the 3′-end of the human mitochondrial NCR, which possesses both a TSS motif and protein requirements for activation identical to those of the canonical mitochondrial promoters LSP and HSP. We also used mitochondrially targeted cytosine base editors (DdCBEs) to manipulate the accumulation of *de novo* transcripts detected at LSP2 in human cell lines to establish its *in vivo* context.

## Results

### A second light strand promoter within the human mitochondrial NCR

The termination-associated sequence (TAS) demarcates the 3′-end of the NCR and is almost immediately followed by tRNA^Pro^, the first gene product of LSP. It has been previously documented that 3′-ends of transcripts arising from neargenome length transcription from HSP also accumulate in this region (Jemt et al., 2015) (Figure 1A), and thus, we re-evaluated whether the TAS region could affect transcription by POLRMT *in vitro*. To test this, we designed a chimeric template containing a region of the NCR encompassing TAS (“3′-NCR”), flanked by HSP to create “3′-NCR+HSP” (Figure 1B). Mitochondrial transcription was reconstituted *in vitro* using PCR-generated DNA templates mixed with recombinant human proteins (POLRMT, TFAM, and TFB2M), where nascent RNAs were labeled by the incorporation of radioactive nucleotides and separated on denaturing polyacrylamide gels.

Transcription of 3′-NCR+HSP by POLRMT produced a ~500 nucleotide (nt) “run-off” (RO) product corresponding to transcription initiated from HSP toward the end of the DNA template (Figures 1B and 1C; “RO,” red arrows). We also observed ~200 nt transcripts shorter than the RO product (P1, Figure 1C, lane 1) that were affected by transition (G↔A/C↔T) mutations within a region between TAS and tRNA^Pro^ (region TAS-A2 to TAS-A7 Figure 1D; the effect of mutations to TAS-A5 is shown as a representative in Figure 1C). To our surprise, these shorter transcripts were still formed on a “promoter-less” template lacking HSP (P1′ products in “3′-NCR(no HSP)”; Figure 1C, lane 3), demonstrating that the observed products did not result from transcription from HSP. Instead, the shorter transcripts (P1 and P1′) were consistent with RO transcription originating from a locus within TAS-A5 and transcribing in the same direction in both 3′-NCR+HSP and 3′-NCR(no HSP) templates (Figure 1B, purple arrows). This direction of transcription additionally explains the truncation of P1 into P1′ (Figure 1B, compare purple arrows in 3′-NCR+HSP and 3′-NCR(no HSP)), since truncations to the DNA template made by omitting HSP (a deletion of 87 bp) also truncated P1 by a similar amount (into P1′). Based on this assumption, the shorter transcripts appeared to occur co-directionally to LSP (i.e., co-directional to tRNA^Pro^) and thus make use of the L-strand as a template for transcription. We therefore designated this locus as “LSP2.”

### The transcription start site of LSP2

**Figure 2:**
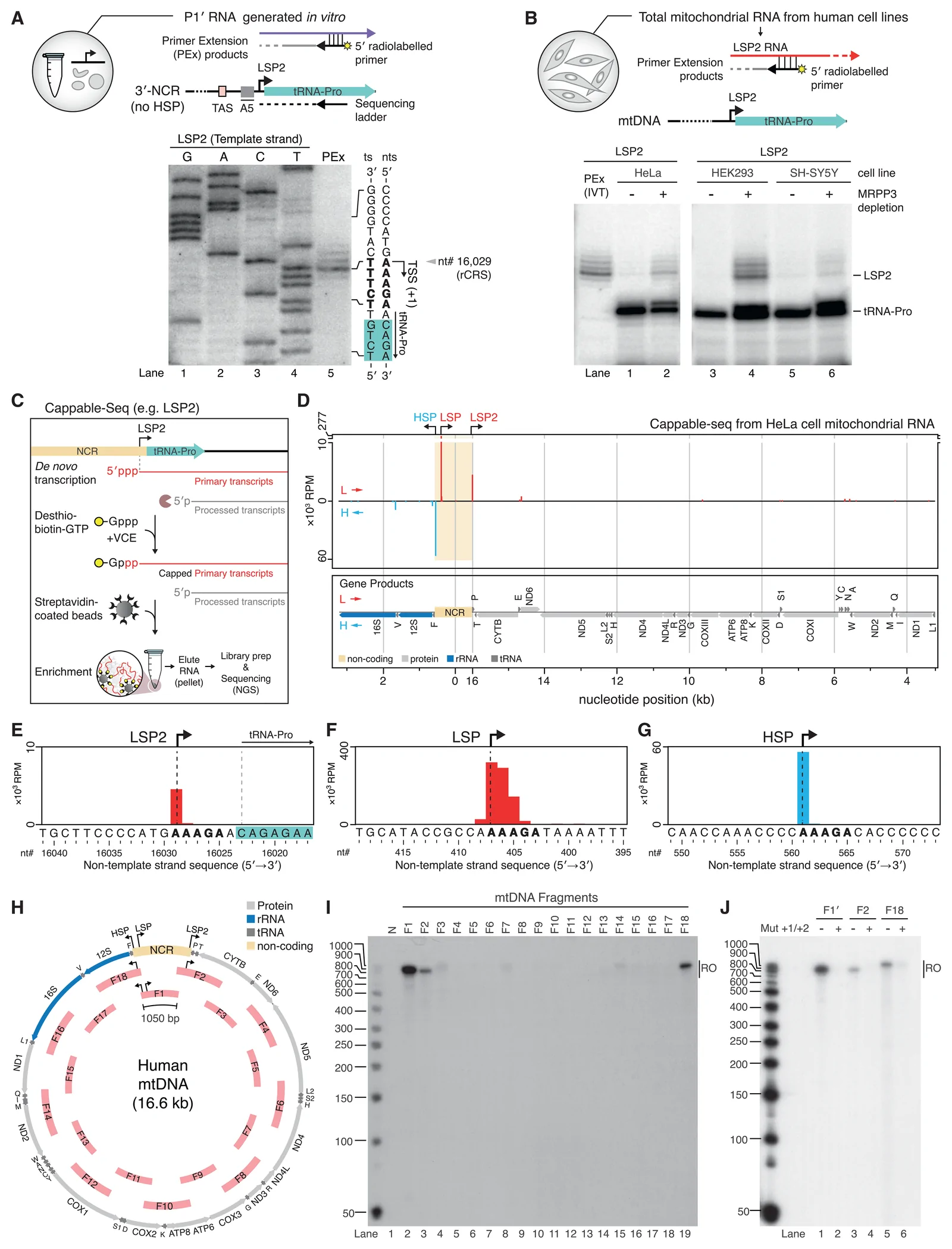
The transcription start site of LSP2 (A) Primer extension (PEx) experiments mapping the 5′-ends of P1′ from Figure 1C. Note that sequencing ladders provide the base identity of the template strand. (B) Mapping the 5′-ends of LSP2 in human cell lines. Primer extension products from *in vitro* transcripts in (A) (“PEx (IVT)” were used as a reference. MRPP3 depletion increases the abundance of detected LSP2 5′-ends. (C) Schematic of the Cappable-seq procedure emphasizing the enrichment of *de novo* synthesized 5′-triphosphorylated (“5′ppp” transcripts (e.g., from LSP2) using the guanylyltransferase activity of the vaccinia capping enzyme (VCE). (D) Cappable-seq profile of 5′-triphosphorylated mitochondrial RNA termini (reads per million, RPM) from the L-strand (red peaks) and H-strand (blue peaks), corresponding to TSS sites in HeLa cell mtDNA. “L” and “H” labels with arrows: direction of transcription from either strand. (E–G) Magnified view of the LSP2, LSP, and HSP loci from Cappable-seq profiles in (D). (H) Schematic of DNA templates F1–F18 (pink), which encompass the entire mitochondrial genome (coordinates listed in Table S5). Both ends of each fragment include a 130 bp sequence overlap with adjacent fragments to account for potential promoters positioned near the terminal ends that may otherwise have been truncated by the template design. (I) *In vitro* transcription products of mtDNA fragments from (H) in the presence of TEFM, separated on 4% urea-PAGE midi gels. “N,” negative control (1.1 kb NdeI/ BsaI fragment of pUC19 blunted with Klenow fragment). “RO,” run-off transcription products from each template. (J) *In vitro* transcription of fragments F1′, F2, and F18 from (I), alongside the same templates carrying G↔T/C↔A mutations at the +1/+2 sites (“Mut +1/+2” of LSP, LSP2, or HSP, respectively, corresponding to 5′-AAAGA-3′ of the TSS motif (based on Figures 2A and S1A–S1C). F1′ is a modified version of F1 that omits HSP (see I).

The primary sequences of the non-template strand in both human LSP and HSP promoters contain a consensus motif of 5′-AAAGA-3′, where transcription initiates primarily from the first adenine residue in this motif (Chang and Clayton, 1984; Gaspari et al., 2004). Note that we refer to the sequence of the non-template strand on the promoter for the majority of this work to emphasize the direction of transcription, as well as the sequence of RNA products. To determine the TSS of LSP2, we mapped the 5′-end of the P1′ product from non-radioactive *in vitro* transcripts of 3′-NCR(no HSP) (used in Figure 1) by primer extension of a radiolabeled DNA probe that anneals to the tRNA^Pro^ region of the predicted P1′ transcript (Figure 2A). Extension of this probe by reverse transcriptase resulted in a dominant complementary DNA (cDNA) product corresponding to an adenine residue at position 16,029 (A_16,029_) of the reference mitochondrial genome (rCRS; Andrews et al., 1999), located 6 nt upstream of tRNA^Pro^. This was the first adenine in a locus containing a motif (5′-**A**AAGA-3′) identical to the TSS of LSP and HSP which we mapped from *in vitro* transcription reactions (Figures S1B and S1C). Primer extension assays also mapped similar 5′-ends corresponding to the TSS of LSP2 in three human cell lines (HeLa, HEK293, and SH-SY5Y) (Figures 2B and S1D–S1G), supporting the relevance of our *in vitro* findings in living cells. These 5′-ends were typically unabundant but were enriched when we depleted MRPP3, the catalytic component of RNase P involved in the 5′-cleavage of pre-tRNA molecules in human mitochondria (Holzmann et al., 2008) (e.g., lane 4, Figure 2B). This suggests that transcripts arising from LSP2 are actively processed by RNase P to form the 5′-ends of tRNA^Pro^. The short distance from tRNA^Pro^ together with efficient processing by RNase P may also help to explain why LSP2 has remained undetected until now.

A distinct hallmark of *de novo* transcription from a TSS is that nascent RNAs are generated with a triphosphate at the 5′-end, whereas processed or capped transcripts instead possess 5′-monophosphorylated ends (Figure 2C). This has been shown previously for LSP and HSP using the guanylyltransferase activity of the vaccinia capping enzyme (VCE), which selectively caps 5′-triphosphorylated species with derivatives of GTP (Montoya et al., 1982). To interrogate whether the same was true for LSP2, we used a modified approach in which 5′-triphosphorylated transcripts were capped with biotinylated GTP, enabling their enrichment on streptavidin beads for high-throughput sequencing (Cappable-seq; Ettwiller et al., 2016; Figures 2C and 2D). Cappable-seq profiles from HeLa cells confirmed distinct 5′-termini mapping to LSP2, as well as at LSP and HSP (Figures 2E–2G), and was consistent with the observation that, among a series of DNA fragments encompassing the mitochondrial genome, only fragments containing LSP, HSP, or LSP2 produced prominent transcription products *in vitro* (Figures 2H–2J). Additionally, we did not detect any 5′-termini corresponding to HSP2. The observation of 5′-triphosphorylated species at the LSP2 locus and the absence of HSP2 transcripts were both also independently reported in a recent dataset using a related technique termed ReCappable-seq (Yan et al., 2022), corroborating our findings.

### Primary sequence requirements of LSP2

**Figure 3:**
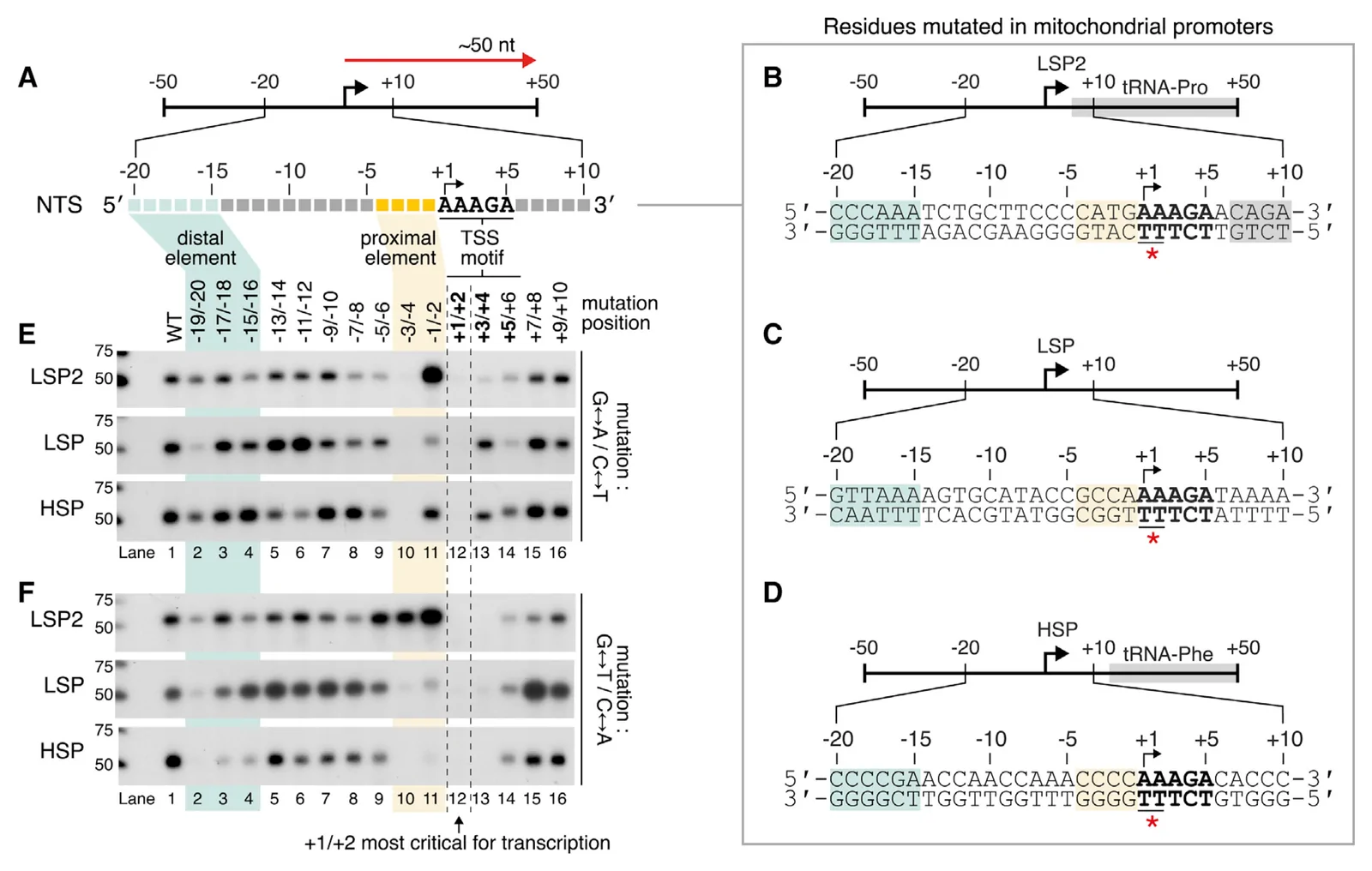
Mutational analysis of mitochondrial promoters (A) Schematic of DNA templates containing -50 to +50 residues relative to the mapped TSS of LSP2, LSP, or HSP wherein a series of 2 bp transition (G↔A/C↔T) or transversion (G↔T/C↔A) mutations have been introduced. Transcription from these templates is expected to produce a 50 nt product (red arrow). (B–D) Wild-type sequences of the mutated region on (B) LSP2, (C) LSP, and (D) HSP templates. The +1/+2 residues are indicated by a red asterisk. (E and F) *In vitro* transcription products of mutant DNA substrates described in (A–D), separated on 8% urea-PAGE mini gels.

Designating the adenine mapped to position 16,029 of the rCRS (A_16,029_) as the TSS (+1) of LSP2, we compared the primary sequence requirements of LSP2 with LSP and HSP using a series of linear DNA templates that had 2 bp transition (G↔A/C↔T) or transversion (G↔T/C↔A) mutations from positions −20 to +10 relative to all three promoters (Figures 3A–3D). Consistent with our assumptions about the TSS of LSP2 from primer extension experiments, both types of mutations to the +1/+2 residues unequivocally abolished RO transcription from all 3 promoters (indicated by an arrow in Figure 2E). We also found that although LSP, HSP, and LSP2 share very little sequence homology apart from the TSS motif, mutations to the −1 to −4 residues (“TSS-proximal element”) also dramatically affected transcription in all three promoters, causing specific changes according to the type of mutation used (e.g., compare HSP mutations in lane 13, Figure 3E). This is in excellent agreement with a similar analysis performed previously for LSP (Gaspari et al., 2004) and demonstrates that the function of the TSS-proximal residues is also conserved at LSP2. However, only at LSP2 did some mutations dramatically increase transcription *in vitro*; for instance, both transition and transversion mutations at the −1G/−2T of LSP2 appeared to stimulate transcription (compare lanes 1 and 11 [LSP2], Figures 3E and 3F). One interesting observation from these experiments is that transition mutations to the −1/−2 residues of LSP and LSP2 exchange the TSS-proximal residues between these promoters, which appear to result in congruent effects. That is, replacing the −1/−2 residues of LSP2 with that of LSP *increases* transcription from LSP2 (compare lanes 1 and 11 [LSP2] in Figure 3E) and conversely replacing the −1/−2 of LSP with that of LSP2 *decreases* transcription from LSP (compare lanes 1 and 11 [LSP], Figure 3E). Taken together, these results demonstrate the importance of the TSS-proximal residues in establishing the relative strengths of mitochondrial promoters.

### The mitochondrial transcription machinery assembles canonically at LSP2

**Figure 4:**
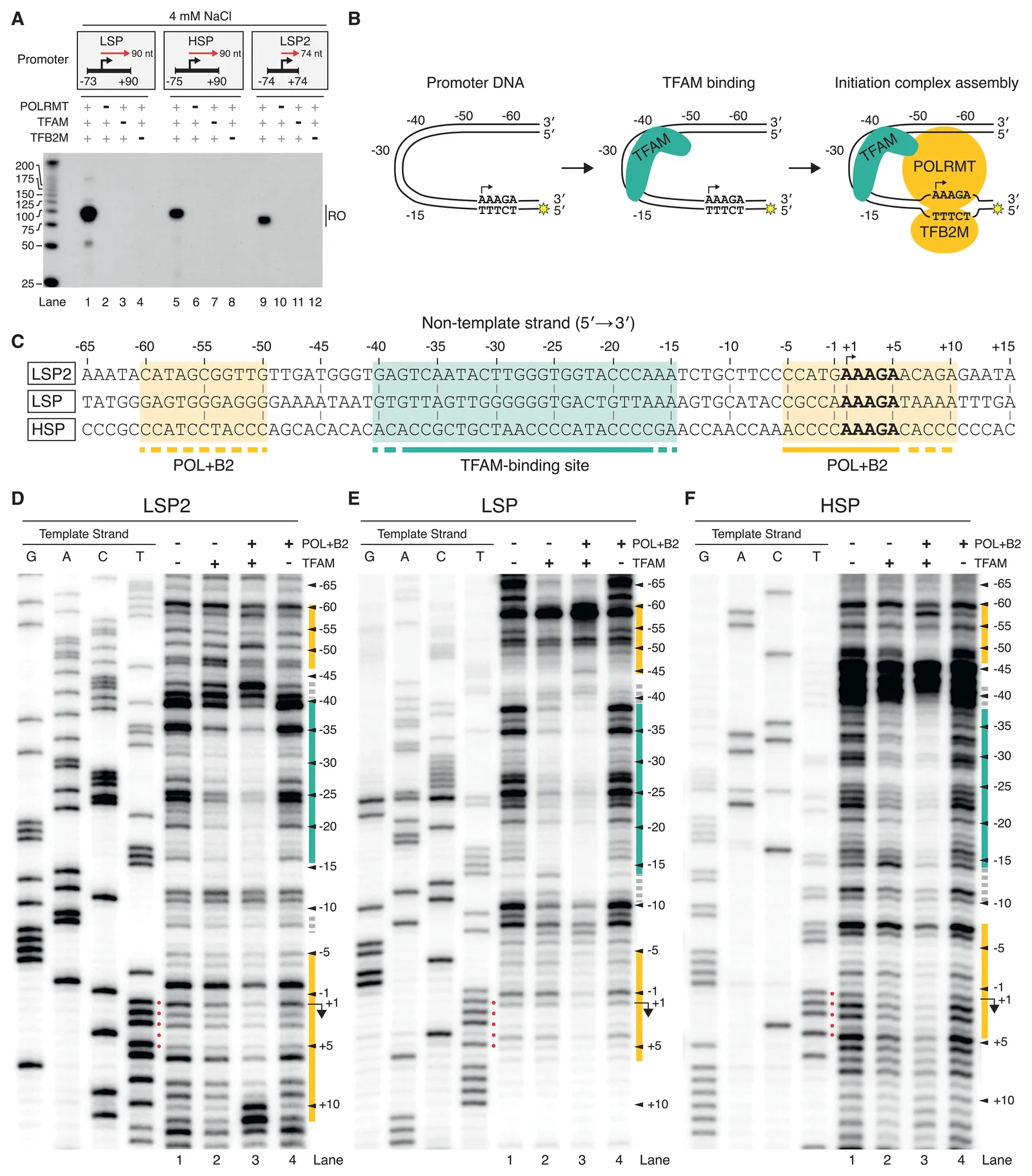
The mitochondrial transcription machinery canonically assembles at LSP2 (A) LSP2 requires POLRMT, TFAM, and TFB2M for transcription. Template schematics for each promoter indicate the length of upstream and downstream bases and the expected sizes of transcripts (red arrows). Run-off (RO) products of *in vitro* transcription are shown below the schematics where either POLRMT, TFAM, or TFB2M were omitted from the reaction. Products were separated on 8% urea-PAGE mini gels. (B) Simplified assembly of the mitochondrial transcription initiation complex where the template DNA strand is 5′-radiolabeled (yellow star) for DNase I footprinting experiments. Note that promoter DNA is not pre-bent prior to TFAM binding. TFAM induces a U-turn structure on the DNA template and recruits POLRMT and TFB2M at the promoter. Regions relative to the TSS are indicated to illustrate interactions of POLRMT, TFAM, and TFB2M with these residues. (C) Primary sequences (non-template strand) of mitochondrial promoters, including putative (LSP, HSP) or predicted (LSP2) TFAM binding sites. Regions in green or orange correspond to mapped sites in footprints shown in (D)–(F). (D–F) DNase I footprinting of mitochondrial promoters using templates (D) LSP2-FP, (E) LSP-FP, and (F) HSP-FP, alongside sequencing ladders to provide base contexts of DNase I cleavage products. “POL+B2” denotes conditions where both POLRMT and TFB2M were added. Regions relative to the promoter (−65 to +10) are labeled based on the location of sequencing ladders (N.B. template strand sequence). Red dots indicate regions of the sequencing ladders corresponding to the consensus TSS motif. Densitometric analysis is provided in Figure S4. DNase I cleavage products were separated on 8% urea-PAGE sequencing gels.

To initiate transcription from mitochondrial promoters, POLRMT requires TFAM and TFB2M as transcription factors (Gustafsson et al., 2016), and in keeping with this, LSP2 also required all three “core” transcription proteins for activity (Figure 4A). These proteins form distinct interactions with mitochondrial promoters to initiate transcription, defined firstly by TFAM binding upstream of the TSS, which distorts DNA into a “U-turn,” and secondly by the recruitment of POLRMT and TFB2M at the TSS (Figure 4B) (Gaspari et al., 2004; Hillen et al., 2017; Morozov and Temiakov, 2016). To interrogate whether such interactions also exist at LSP2, we generated DNA templates by PCR containing LSP, HSP, or LSP2, where the 5′-end of the template strand was selectively radiolabeled (yellow star on the substrate in Figure 4A). These templates were incubated with different combinations of the core transcription proteins under conditions that promote the formation of the initiation complex. We then used DNase I footprinting to probe the occupancy of the transcription proteins on the templates.

In the presence of TFAM alone, a clear TFAM footprint was observed at LSP2 encompassing positions −15 to −40 (Figure 4C, lane 2), identical to the TFAM binding sites upstream of the TSS at LSP and HSP (Figures 4D and 4E, lane 2) and notably containing the G/C-N_10_-G/C TFAM recognition motif spanning positions −20 to −31 that was recently characterized for LSP and HSP (Choi and Garcia-Diaz, 2022). This TFAM footprint was enhanced in the combined presence of POLRMT, TFB2M, and TFAM, which is likely the result of more stable TFAM interactions arising from the formation of the initiation complex. In the presence of POLRMT, TFB2M, and TFAM, additional DNase I protection was also observed at regions encompassing the TSS (e.g., −5 to +5) for all promoters, indicative of the binding of both POLRMT and TFB2M at the TSS (Figures 4D–4F, lane 3). Under the same conditions, we also observed subtle changes in the cleavage patterns further upstream of the TFAM binding site around the −50 region in all promoters. We take this to indicate additional contacts from POLRMT and TFB2M resulting from the TFAM-induced U-turn during transcription initiation (Figure 4A). Densitometric analyses of footprinting experiments are provided in Figure S4 to emphasize the changes in DNase I cleavage observed at both the TFAM binding site and the TSS in the presence of all three transcription proteins. The TFAM binding site found for LSP2 is required for transcription, as mutations to the TAS-A2 to TAS-A4 regions that encompass the TFAM binding site abolished transcription from LSP2 (Figures 1D; P1 products, coordinates in Table S4). Collectively, this offers evidence that the assembly of the initiation complex at LSP2 is identical to that of LSP and HSP and is in agreement with structural and biochemical work performed on LSP and HSP (Gaspari et al., 2004; Hillen et al., 2017; Morozov and Temiakov, 2016).

### LSP2 is functionally conserved among humans and apes

**Figure 5:**
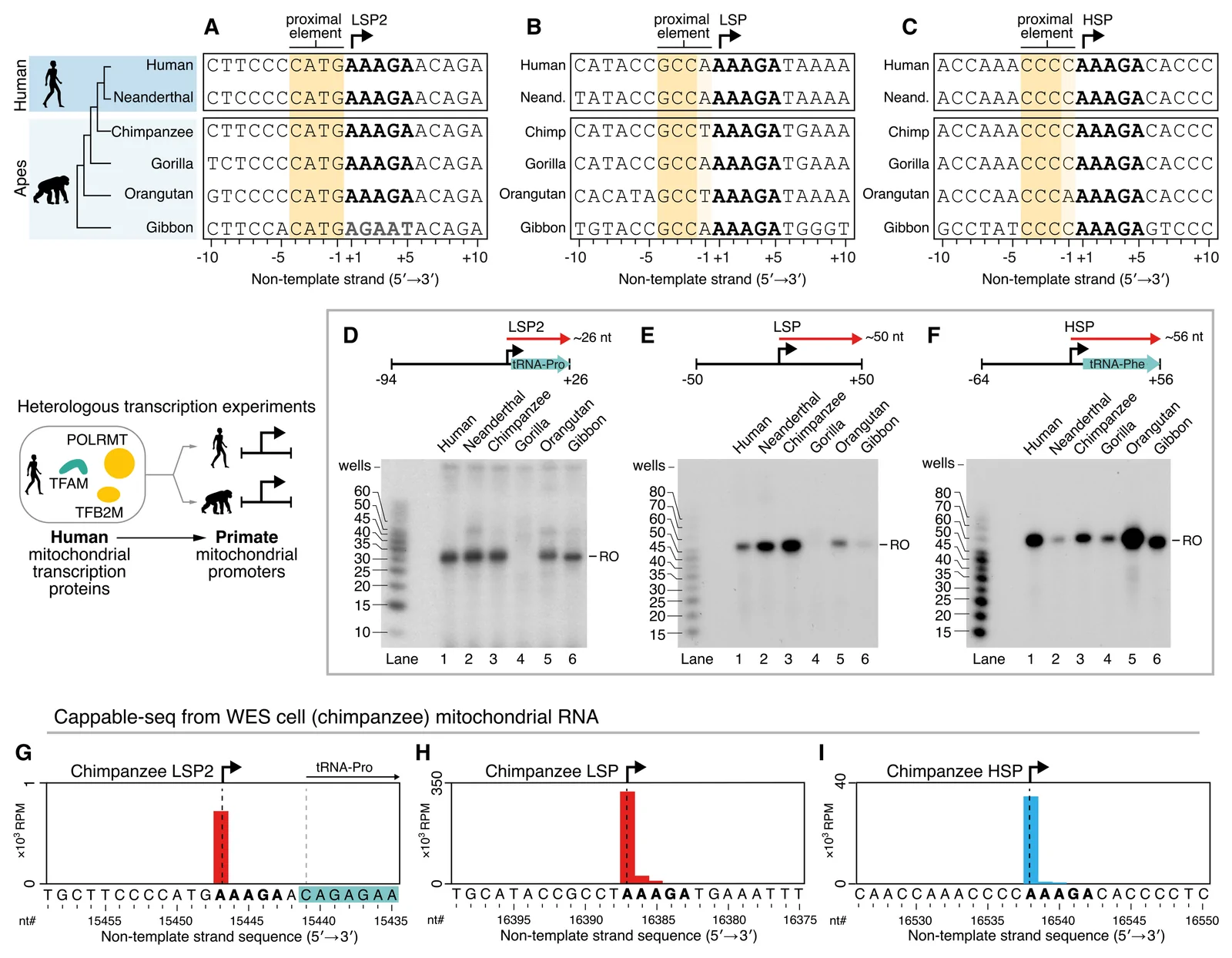
LSP2 is functionally conserved in primates (A–C) Sequence alignments of (A) LSP2, (B) LSP, and (C) HSP among human and ape mtDNA (phylogeny branch lengths not to scale), highlighting residues of the TSS-proximal element. (D–F) Human mitochondrial transcription proteins can use primate promoter sequences as templates for transcription. Schematic of DNA templates encompassing respective promoter regions in humans and apes are indicated, along with the expected size of transcription products. Products of *in vitro* transcription using human POLRMT, TFB2M, and TFAM are shown below each schematic, separated on 15% urea-PAGE mini gels. (G–I) Cappable-seq profiles of 5′-triphosphorylated mitochondrial RNA termini (reads per million, RPM) at (G) LSP2, (H) LSP, and (I) HSP, corresponding to TSS sites in WES (chimpanzee) cell mtDNA. Global Cappable-seq profiles can be found in Figure S3.

We found from sequence alignments that the TSS-proximal residues of LSP2, which are critical for transcription from mitochondrial promoters (Figure 3), were particularly well conserved between humans, Neanderthals, and apes (Figures 5A–5C). A more comprehensive series of point mutations to these residues in all three human promoters showed a dramatic effect on transcription *in vitro* (Figures S2A–S2I). However, although some of these mutations can increase transcription from LSP2 (e.g., lanes 4 and 6, Figure S2G), these were not tolerated in the mitochondrial genomes of human cohorts (Turro et al., 2020; Wei et al., 2017, 2019) (Figures S2J–S2L). In support of the evolutionary conservation of LSP2 among primates, ape mitochondrial promoters were in most cases able to support heterologous transcription *in vitro* using human proteins (Figures 5D–5F). Furthermore, in a cell line derived from chimpanzees (WES), the closest-related ape species to humans, we were able to detect *de novo* transcripts mapping to LSP, HSP, and LSP2 by Cappable-seq (Figures 5G–5I and S3). The intensity of LSP2 peaks in WES cells were comparatively lower than in human profiles (compare Figure 5G with Figure 2F), which could be due to differences in the activity of LSP2 or differences in the efficiency of mitochondrial RNA processing. We also found L-strand transcripts mapping to an A/T-rich locus at tRNA^Glu^ (Figure S3E) that were not observed in Cappable-seq profiles from human cell lines, potentially highlighting divergent mechanisms of mtDNA transcription between humans and chimpanzees.

### LSP2 is regulated by TFAM and is more sensitive to the topology of the DNA template

**Figure 6:**
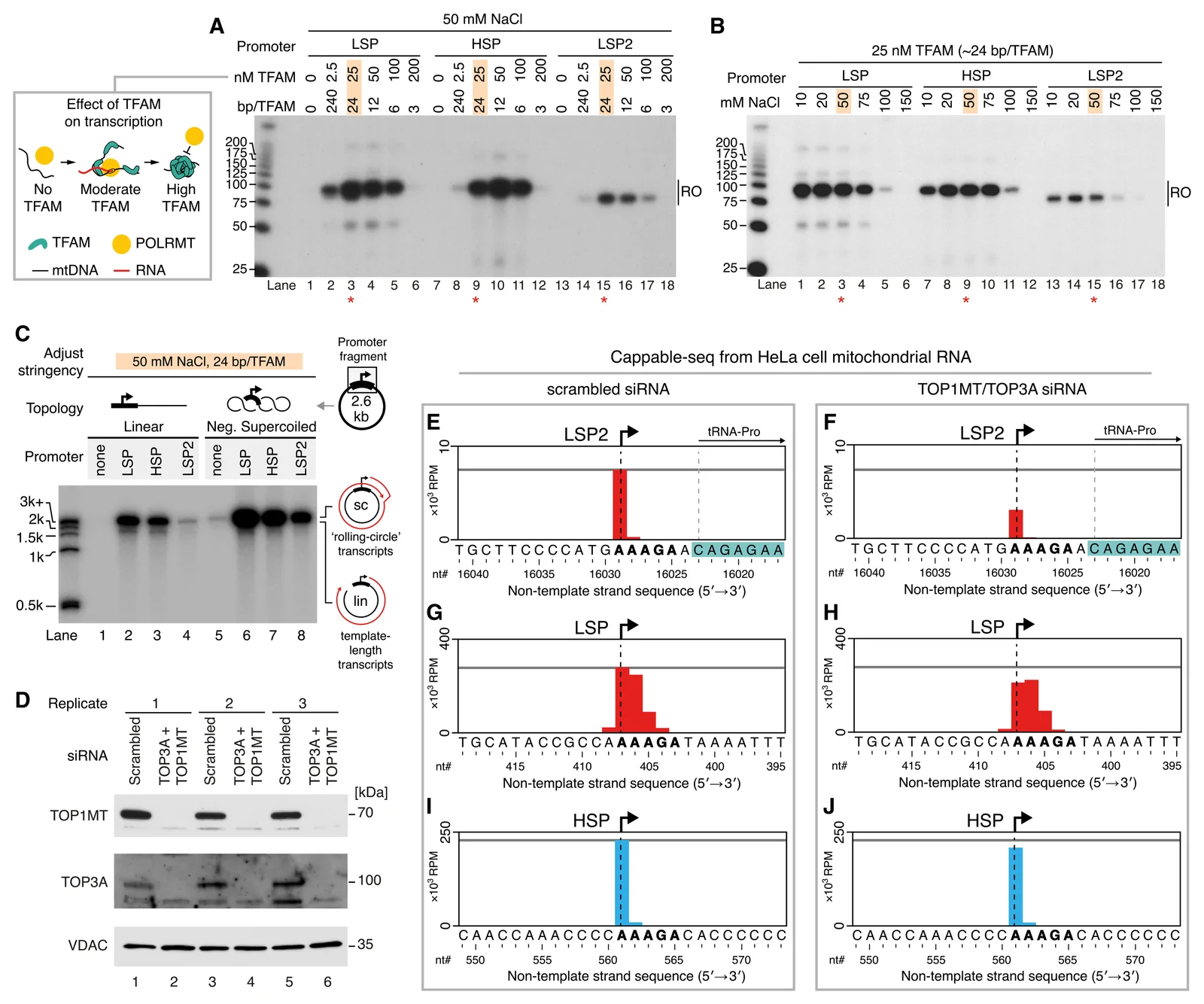
LSP2 transcription is regulated by TFAM and is sensitive to the topology of template DNA (A and B) Effect of TFAM and salt titrations on LSP2 transcription *in vitro*. Promoter fragments were the same as those in Figure 4A. (A) TFAM titrations with 50 mM NaCl. The ratio of the DNA template to TFAM (bp/TFAM) per concentration of TFAM is indicated. (B) NaCl titrations, at ~24 bp/TFAM. Orange boxes and red asterisks in (A) and (B) mark lanes and conditions chosen for experiments in (C). Products were separated on 8% urea-PAGE mini gels. (C) The effect of DNA topology on transcription from LSP2. *In vitro* transcription was carried out on plasmids carrying the same promoter fragments used in (A) and (B), either linearized upstream of the promoter with HindIII (“linear,” “lin”, or in their native negatively supercoiled form (“neg. supercoiled,” “sc”. Human TEFM also was included in these reactions to encourage processive transcription of the long RNA products. pUC19 was used as a negative control to represent templates lacking a mitochondrial promoter (lanes 1 and 5), from which transcripts observed from supercoiled templates (lane 5) likely occur due to non-specific transcription initiation. Products were separated on 4% urea-PAGE mini gels. (D) Western blots illustrating effective siRNA-mediated depletion of both TOP1MT and TOP3A in HeLa cells. VDAC was used as a loading control. (E–J) Cappable-seq profiles (reads per million, RPM) from control and TOP1MT/TOP3A-depleted HeLa cells at (E and F) LSP2, (G and H) LSP, and (I and J) HSP. Horizontal gray lines in indicate peak heights from control profiles. Global Cappable-seq profiles are shown in Figure S5.

TFAM is a ubiquitous mitochondrial protein, and in addition to its role at mitochondrial promoters, it can also compact mtDNA into nucleoids (Kukat et al., 2015). Low to moderate TFAM concentrations relative to the DNA template can stimulate transcription, whereas high TFAM concentrations, which induce dense DNA compaction *in vitro*, can attenuate transcription from LSP and HSP (Farge et al., 2014; Shi et al., 2012). We observed the same effect with LSP2 (Figure 6A). This is in contrast to the activity reported for HSP2 *in vitro*, which is instead repressed by TFAM (Lodeiro et al., 2012). Furthermore, LSP2 appeared to be less active than LSP and HSP at elevated salt concentrations (Figure 6B).

We also examined whether the topology of the DNA template could influence the magnitude of transcription initiation from LSP2. *In vivo*, DNA is typically maintained in an underwound (negatively supercoiled) state, which stimulates transcription initiation by reducing the energy required to unwind duplex promoter DNA (Drew et al., 1985; Ma and Wang, 2016; Strick et al., 1998). Negative supercoiling can also lead to non-specific initiation, especially at low ionic strength, but in the context of mitochondrial promoters, the occupancy of TFAM on DNA can help to prevent spontaneous unwinding of duplex DNA, allowing promoter-specific transcription to predominate over non-specific events. Since our transcription assays thus far used linear DNA templates, we considered the effect of negatively supercoiled DNA on LSP2 transcription. Using salt and TFAM concentrations that on linear DNA templates virtually inhibited LSP2 while still supporting transcription from LSP and HSP (indicated in Figures 6B and 6C), we carried out transcription reactions on linear versus negatively supercoiled plasmid DNA templates containing the same promoter fragments used in Figure 4A. We also included the elongation factor TEFM in these reactions to promote processive transcription of the long DNA templates used (Agaronyan et al., 2015; Posse et al., 2015). Under these conditions, transcription from LSP2 was markedly lower than from LSP and HSP on linear templates (Figure 6D, lanes 1–4) but was increased on negatively supercoiled templates to levels more comparable with those of LSP and HSP (Figure 6C, compare lanes 4 and 8).

Topological stress *in vivo* is removed by topoisomerases; enzymes that create transient breaks in DNA to relieve supercoiling or to remove DNA interlinkages. The topoisomerases TOP3A and TOP1MT localize to mitochondria in human cells (Wang et al., 2002; Zhang et al., 2001), and their simultaneous depletion dysregulates mtDNA topology, resulting in severe defects in mtDNA maintenance (Nicholls et al., 2018). We additionally performed Cappable-seq analysis of cells depleted of TOP3A and TOP1MT, which showed a selective loss of transcripts initiating at LSP2 relative to those at LSP or HSP (Figures 6E–6J). This supports the conclusion from *in vitro* data (Figure 6D) that the activity of LSP2 is highly sensitive to the topological state of the template DNA.

### Transcriptional alterations in LSP2-edited cells parallel *in vitro* effects

**Figure 7:**
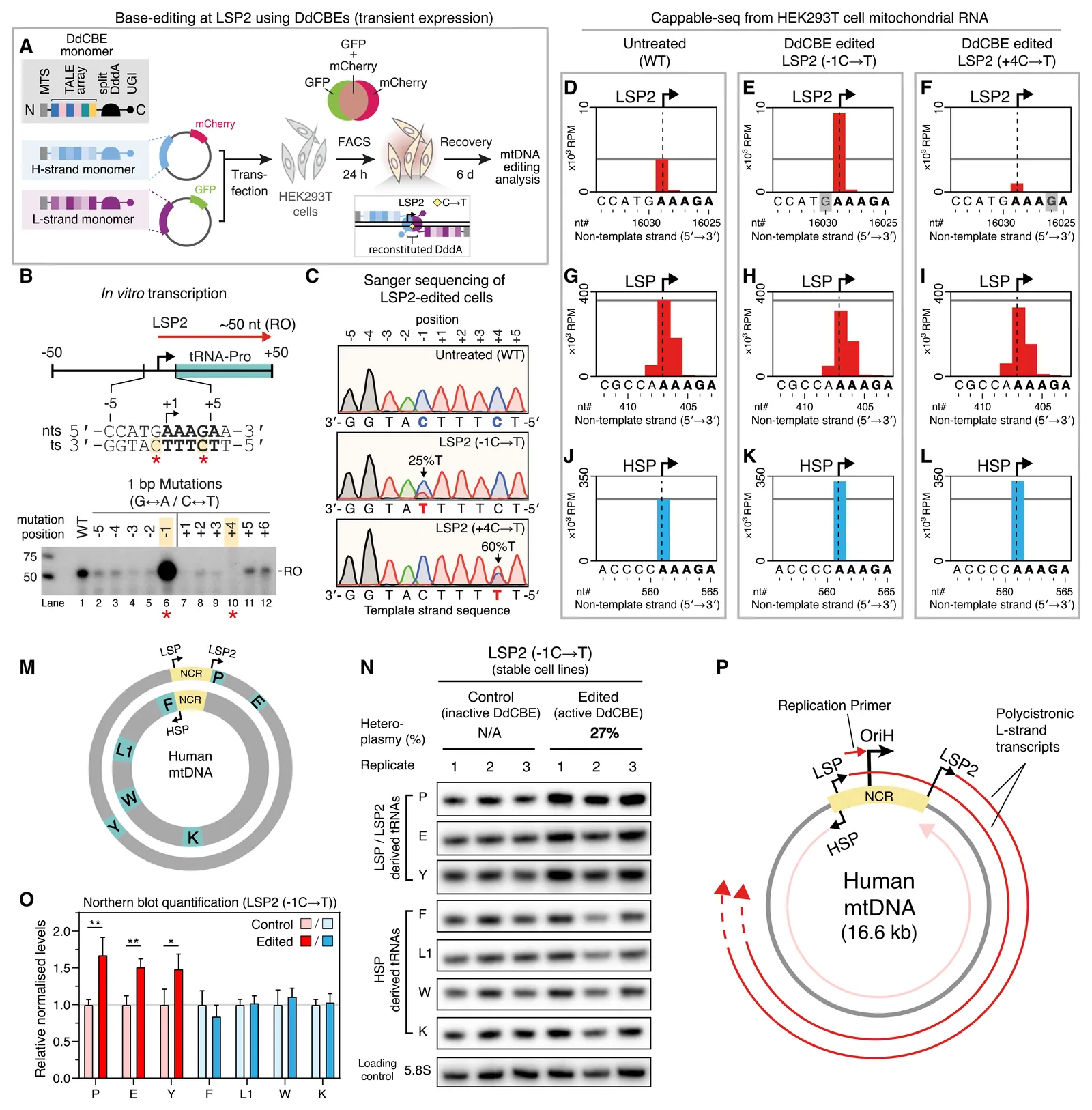
Transcriptional alterations in LSP2-edited cells parallel *in vitro* effects (A) Workflow schematic of DdCBE-induced base editing of mtDNA in HEK293T cells. MTS, mitochondrial targeting sequence; TALE, transcription activator-like effector array (targeting a specific genetic locus), DddA, a split fragment of the DddA_tox_ cytosine deaminase; UGI, uracil DNA glycosylase inhibitor. (B) The effect of C→T mutations of the -1 and +4 residues of LSP2 (red asterisks) on run-off (RO) transcription *in vitro* (cropped from Figure S2D). A template schematic (red arrow: transcription products) is shown in which a series of residues at LSP2 have been mutated. (C) Sanger sequencing profiles of the LSP2 locus showing heteroplasmies in DdCBE-edited cells. (D–L) The effect of LSP2 mutations on *de novo* mitochondrial transcription in cultured human cells. Cappable-seq profiles at (D–F) LSP2, (G–I) LSP, and (J–L) HSP are shown comparing the abundance of 5′-triphosphorylated mitochondrial transcripts (reads per million, RPM) in control (“untreated”) and LSP2-edited (“DdCBE-edited”) HEK293T cells. Horizontal gray lines in graphs indicate peak heights from control profiles. Global Cappable-seq profiles: Figure S7. (M–O) The LSP2(-1C→T) mutation affects steady-state L-strand tRNA levels. (M) Position on the human mitochondrial genome of LSP/LSP2 or HSP-derived tRNAs (1-letter amino acid code) probed in (N). (N) Northern blots of mitochondrial tRNAs from stable cell lines expressing mtDdCBEs targeting the -1 site of LSP2. (O) Mean tRNA levels relative to control cells quantified from northern blots (n = 3, error bars SD); statistical significance (indicated by asterisks) was computed from a one-tailed Student’s t test (p ≤ 0.05). (P) Working model summarizing the position and possible role of LSP2 in the human mitochondrial genome. Transcription from LSP produces prematurely terminated transcripts that are thought to form replication primers upstream of the origin of leading strand mtDNA synthesis (OriH), as well as polycistronic L-strand mRNA. LSP2 can also synthesize L-strand genes independent of primer formation at OriH.

The study of mitochondrial gene expression using reverse genetics has historically been precluded by an inability to genetically transform mammalian mitochondria (Silva-Pinheiro and Minczuk, 2022). Recently, a mitochondrially targeted DddA-derived cytosine base editor (DdCBE) was developed, which opened up the possibility of introducing precise C→T mutations in the mtDNA of living cells in a 5′-T**C**/**G**A-3′ context (Mok et al., 2020). These mitochondrially targeted DdCBEs (mtDdCBE) essentially consist of a transcription activator-like effector (TALE) array targeting a specific genetic locus, which is fused to one of two split fragments of the DddA_tox_ cytosine deaminase (DddA). Base editing is achieved from a two-vector system expressing mtDdCBEs with TALEs that target either the L- or H-strand flanking a desired mtDNA locus, which brings the split DddA fragments into close proximity and reconstitutes the active enzyme for base editing. Based on this technology, we have adopted a modified approach in which each vector additionally contains a fluorescent reporter, which allows HEK293T cells transfected with both plasmids to be enriched by fluorescence-activated cell sorting (FACS) (Figure 7A).

Using mtDdCBEs, we investigated whether the *in vitro* effect of two such mutations at LSP2 (−1C→T and +4C→T), which can increase or decrease transcription, respectively (Figure 7B), could be recapitulated in cultured human cells. Note that both of these cytosine bases are located on the *template* strand of LSP2. Wild-type and mutant variants of mtDNA can co-exist in a single cell (a phenomenon termed heteroplasmy), and in HEK293T cells transiently expressing mtDdCBEs targeting LSP2, we achieved heteroplasmy levels of either ~25% sustaining LSP2(−1C→T) or ~60% sustaining LSP2(+4C→T) (Figure 7C). These were in line with the 5%–50% range originally reported for DdCBEs (Mok et al., 2020). Remarkably, Cappable-seq profiles revealed that the level of *de novo* transcripts mapping to LSP2 increased in cells containing LSP2(−1C→T) edits and decreased in cells containing LSP2(+4C→T) edits, relative to untreated cells (Figures 7D–7F). In contrast, LSP and HSP did not exhibit a similar effect (Figures 7G–7L). These data are in excellent agreement with our *in vitro* findings (Figure 7B).

To investigate the involvement of LSP2 in mitochondrial gene transcription, we focused on the LSP2(−1C→T) mutation, reasoning that increased LSP2 transcription from this mutation would have an additive and therefore straightforward effect on steady-state L-strand transcript levels. To this end, we used the Flp-In system to generate cell lines stably expressing mtDdCBEs, thereby allowing us to analyze mitochondrial gene expression in cells that can actively sustain the LSP2 (−1C→T) mutation. Although this approach also aimed to increase the heteroplasmy of the LSP2(−1C→T) edits, it still appeared to persist within a similar range (~27% in stable cell lines [Figure S7E] versus ~25% from transient expression), possibly indicating that this level of heteroplasmy is the upper limit tolerated by cells and that the −1C→T mutation is subject to negative selection.

Steady-state levels of mitochondrial gene transcripts (mRNAs, tRNAs, and rRNAs) are each tightly regulated by independent and strand-specific pathways, with rRNAs being exclusively H-strand encoded (Rackham and Filipovska, 2022). Additionally, both mature and precursor transcripts of *ND6*, the only mRNA encoded by the L-strand, are rapidly turned over (Aloni and Attardi, 1971; Ojala et al., 1981) by a mechanism that is still poorly understood (Jedynak-Slyvka et al., 2021; Ohkubo et al., 2021). Therefore, we chose to inspect the effect of the LSP2(−1C→T) mutation on mito-chondrial tRNAs, which are more stable (Aloni and Attardi, 1971; King and Attardi, 1993) and more sensitive to changes in *de novo* transcription (Park et al., 2007). Consistent with an expected increase in transcription from LSP2, stable cell lines carrying LSP2(−1C→T) edits showed a significant increase in the steady-state levels of L-strand-derived tRNAs (P, E, and Y) relative to control cells expressing catalytically inactive mtDdCBEs (Figures 7M–7O)—an effect that was remarkable given the relatively low heteroplasmy (~27%) of LSP2-edited mtDNA. In contrast, H-strand-derived tRNAs were unaffected. This result supports the *in vivo* context of LSP2 as a bona fide promoter that participates in mitochondrial gene expression.

## Discussion

We report the discovery and characterization of a second promoter within the L-strand of human mtDNA, which we term LSP2. The promoters of human mtDNA (LSP and HSP) were first mapped using guanylyltransferase capping (Chang and Clayton, 1984; Montoya et al., 1982), and in this work, we have used a deep-sequencing method (Cappable-seq) derived from guany-lyltransferase capping to identify mitochondrial TSSs in human cells (Figures 2C–2G). This, together with primer extension and extensive biochemical characterization, establishes LSP2 as an additional bona fide mitochondrial promoter. We suggest that the endonucleolytic cleavage of triphosphorylated polycistronic transcripts by the RNase P complex, and the subsequent rapid degradation of non-coding RNA fragments, accounts for why LSP2 has not been previously discovered (Figure 2B). Notably, we found no evidence of transcription initiation from the proposed additional H-strand promoter HSP2, in agreement with another recent Cappable-seq dataset (Yan et al., 2022). Finally, we show that increased transcription from LSP2 induced by −1C→T mutations leads to an increase in steady-state levels of L-strand-derived tRNAs (Figure 7N), suggesting that LSP2 is involved in mitochondrial gene expression.

LSP2 shares core characteristics of the established mtDNA promoters LSP and HSP. All three promoters share a core sequence motif, 5′-AAAGA-3′, with transcription predominantly initiating from the 5′ nucleotide of this motif. All three promoters strictly require the three core proteins of the mitochondrial transcription machinery (POLRMT, TFAM, and TFB2M) for activity, with footprinting analyses indicating that the structure of the initiation complex is conserved between all three promoters. Interestingly, despite these similarities, we found two notable differences in the activity and regulation of LSP2 in comparison with LSP and HSP. First, LSP2 appears to be less active than LSP (Figure 2D, and at elevated salt and TFAM concentrations; Figure 6C); however, transversion mutations in the TSS-proximal motif of LSP2 that bring the sequence in line with that of LSP cause a dramatic increase in transcription activity *in vitro* (Figures 3, 7B, and S2A–S2I). These mutations do not appear to persist in humans (Figure S2) and may indicate that there is a selective pressure toward maintaining low LSP2 activity, possibly to maintain the balance of replication primers versus polycistronic transcripts. Second, the activity of LSP2 appears to be influenced by DNA template topology both *in vitro* and in human cells (Figure 6) in a manner not observed for LSP or HSP. We speculate that this unique property of LSP2 could be used to independently regulate its activity *in vivo*, promoted for instance by TFAM-induced negative supercoiling of mtDNA (Shi et al., 2012). Conversely, negative supercoiling could be reduced during mtDNA replication, as positive supercoiling is expected to accumulate ahead of the replication fork. This could intrinsically prevent concomitant L-strand gene transcription from LSP2 during mtDNA replication, particularly since LSP2 is positioned downstream of OriH (Figures 1A and 7P).

The prevailing model in which mtDNA replication and gene expression are regulated by a switch in POLRMT activity is based on the long-held assumption that L-strand transcription can only occur from one site (LSP) (Agaronyan et al., 2015; Chang and Clayton, 1985; Pham et al., 2006; Wanrooij et al., 2010) (Figure 1A). However, the position of LSP2 (downstream of the region in which the replication-transcription switch occurs and immediately upstream of the first L-strand gene) suggests that the complete repertoire of L-strand gene products can in fact be expressed from a site distinct from the synthesis of replication primers. This offers a new perspective on the control of mitochondrial gene expression that primarily builds on studies that mapped LSP and HSP in human cells 40 years ago (Chang and Clayton, 1984; Montoya et al., 1982). We speculate that the location of LSP2 may allow L-strand gene transcription to be decoupled from primer formation, such that under some conditions LSP may be predominantly used for primer formation, whereas LSP2 can serve as the promoter used to synthesize L-strand genes (Figure 7P). Separating mtDNA replication and gene transcription in this way could potentially avoid genome instability associated with coordinating these events from a single promoter. This could be relevant, for example, in post-mitotic cells such as cardiac and skeletal muscles, which have a high metabolic requirement and thus may need to tailor elevated levels of mitochondrial gene expression to meet an intrinsically high ATP demand, but which have a low requirement for mtDNA replication (Lawless et al., 2020). These cell types are also among the most frequently affected by mitochondrial disease (Gorman et al., 2016).

### Limitations of the study

Although our work establishes the existence of LSP2 as a bona fide mitochondrial promoter in humans, its physiological function remains an open question. Additionally, our data suggest that LSP2 is potentially weaker than LSP in human cells and so does not preclude the likely dual role of LSP in both replication primer synthesis and mitochondrial gene transcription. A detailed analysis of the physiological aspects of LSP and LSP2 function will most likely be rewarding but requires the development of new techniques that can unambiguously monitor the relative activity of these two promoters in cells and tissues under physiologically relevant conditions. In line with this, future work should seek to address, for example, whether the activity of LSP2 is differentially regulated in certain tissue types and between rapidly dividing and post-mitotic cells.

## Star⋆Methods

### Key Resources Table

**Table:** 

REAGENT or RESOURCE	SOURCE	IDENTIFIER
Antibodies		
Rabbit anti-MRPP3, monoclonal	Abcam	Cat# ab185942
Mouse anti-β-Actin, monoclonal	Abcam	Cat# ab6276, RRID:AB_2223210
HRP sheep anti-mouse IgG	GE Healthcare	Cat# NXA931, RRID:AB_772209
HRP donkey anti-rabbit IgG	GE Healthcare	Cat# NA9340, RRID:AB_772191
Mouse anti-iTOP1MT, polyclonal	Proteintech	Cat# 16540-1-AP, RRID:AB_2878274
Rabbit anti-TOP3A, polyclonal	Proteintech	Cat# 14525-1-AP, RRID:AB_2205881
Mouse Anti-VDAC1, monoclonal	Abcam	Cat# ab14734, RRID:AB_443084
Rabbit anti-mouse HRP, polyclonal	Agilent	Cat# P0260, RRID:AB_2636929
Swine anti-rabbit HRP, polyclonal	Agilent	Cat# P0217, RRID:AB_2728719
Bacterial and virus strains		
OneShot Top10 Chemically competent *E. coli* cells	Invitrogen	Cat# C404003
Chemicals, peptides, and recombinant proteins		
Benzamidine hydrochloride hydrate	Sigma-Aldrich	Cat# B6506
Phenylmethylsulfonyl fluoride (PMSF)	Sigma-Aldrich	Cat# P7626
Pepstatin	Thermo Fisher Scientific	Cat# BP2671-250
Leupeptin	Serva	Cat# 51867-03
Triton X-100	Sigma-Aldrich	Cat# T8787
D-Mannitol	Sigma-Aldrich	Cat# M4125
D(+)-Sucrose	VWR chemicals Sweden	Cat# CA97061-428
TRIzol reagent	Thermo Fisher Scientific	Cat# 15596026
Dulbecco’s modified Eagle’s medium (DMEM)	Thermo Fisher Scientific	Cat# 10569010
Foetal bovine serum (FBS, heat-inactivated)	Thermo Fisher Scientific	Cat# A3840402
Penicillin-streptomycin solution	Thermo Fisher Scientific	Cat# 15140122
Nuclease-free water (not DEPC-Treated)	Ambion	Cat# AM9937
Ampicillin, sodium salt	Serva	Cat# 13399.02
Glycogen	Roche	Cat# 10901393001
GTP, 100 mM	Thermo Fisher Scientific	Cat# R0461
CTP, 100 mM	Thermo Fisher Scientific	Cat# R0451
ATP, 100 mM	Thermo Fisher Scientific	Cat# R0441
UTP, 100 mM	Thermo Fisher Scientific	Cat# R0471
α-32P-UTP (3000 Ci/mmol)	Hartmann Analytic GmbH	Cat# SRP-210
γ-32P-ATP (3000 Ci/mmol)	Hartmann Analytic GmbH	Cat# SRP-301
α-32P-dATP (3000 Ci/mmol)	Hartmann Analytic GmbH	Cat# SRP-203
DNase I (1 U/μL)	Thermo Fisher Scientific	Cat# EN0521
Turbo DNase (2 U/μL)	Thermo Fisher Scientific	Cat# AM2238
Transcriptor Reverse transcriptase	Roche	Cat# 3531317001
Proteinase K	Ambion	Cat# AM2546
HindIII-HF	New England Biolabs	Cat# R3104S
Antarctic Phosphatase	New England Biolabs	Cat# M0289S
T4 Polynucleotide Kinase (T4 PNK)	New England Biolabs	Cat# M0201S
RNase Inhibitor, murine (40 U/μL)	New England Biolabs	Cat# M0314S
Bovine Serum Albumin (BSA), Molecular Biology Grade (20 mg/mL)	New England Biolabs	Cat# B9000S
Phusion High-Fidelity DNA Polymerase	New England Biolabs	Cat# M0530L
PerfectHyb™ PLUS hybridisation buffer	Sigma-Aldrich	Cat# H7003
Recombinant human POLRMT	Falkenberg et al., 2002	N/A
Recombinant human TFAM	Falkenberg et al., 2002	N/A
Recombinant human TFB2M	Falkenberg et al., 2002	N/A
Recombinant human TEFM	Posse et al., 2015	N/A
Critical commercial assays		
QIAquick PCR purification kit	Qiagen	Cat# 28104
QiaEXii gel extraction kit	Qiagen	Cat# 20021
Qiaprep Spin Miniprep kit	Qiagen	Cat# 27106X4
Microspin G25 size exclusion columns	GE Healthcare	Cat# GE27-5325-01
USB Sequenase Version 2.0 DNA Polymerase	Affymetrix	Cat# 70775Y/Z
Lipofectamine RNAiMAX	Thermo Fisher Scientific	Cat# 13778075
SuperSignal™ West Dura Extended Duration Substrate kit	Thermo Fisher Scientific	Cat# 34075
SuperSignal West Pico Plus	Pierce	Cat# 34580
DNeasy Blood and Tissue kit	Qiagen	Cat# 69504
Quick RNA Micro prep kit	Zymo Research	Cat# R1050
RNeasy mini kit	Qiagen	Cat# 74106
FuGENE-HD	Promega	Cat# 32042
5′/3′ RACE kit (2^nd^ Generation)	Roche	Cat# 03353621001
RNA clean and concentrator-5 kit	Zymo Research	Cat# R1013
Phusion High-Fidelity PCR Master Mix	New England Biolabs	Cat# M0531
Hybond N+ Hybridization membrane	Cytiva	Cat# RPN303B
Deposited data		
Cappable-seq raw data	This study	GEO: GSE176046
Uncropped gel images	This study	https://doi.org/10.17632/s5434tzz78.1
Human reference mitochondrial genome	Andrews et al., 1999	GenBank: NC_012920
Neanderthal reference mitochondrial genome	Green et al., 2008	GenBank: AM948965
Chimpanzee reference mitochondrial genome	Horai et al., 1995	GenBank: NC_001643
Gorilla reference mitochondrial genome	Horai et al., 1995	GenBank: NC_001645
Orangutan (Sumatran) reference mitochondrial genome	Xu and Arnason, 1996	GenBank: NC_002083
Lar Gibbon reference mitochondrial genome	(Arnason et al., 1996)	GenBank: NC_002082
Experimental models: Cell lines		
Human: HeLa	ATCC	CCL-2, RRID:CVCL_0030
Human: HEK293	ATCC	CRL-1573, RRID:CVCL_0045
Human: HEK293T	ATCC	Cat# CRL-3216, RRID:CVCL_0063
Human: SH-SY5Y	Cell line service (CLS)	300154, RRID:CVCL_0019
Chimpanzee: WES	European Collection of Authenticated Cell Cultures (ECACC)	91011612, RRID:CVCL_4337
Oligonucleotides		
10-100 bp low molecular weight DNA ladder	Affymetrix	Cat# J76410
Low molecular weight DNA ladder	New England Biolabs	Cat# N3233S
25 bp DNA step ladder	Promega	Cat# G451A
50 bp Generuler	Thermo Fisher Scientific	Cat# SM0371
MRPP3 siRNA	Horizon Discovery	Cat# M-020859-01-0050
Non-targeting control siRNA	Horizon Discovery	Cat# D-001210-01-50
TOP1MT siRNA	Thermo Fisher Scientific	Cat# 4392420
TOP3A siRNA	Thermo Fisher Scientific	Cat# 4392420
PCR templates and primers used to generate *in vitro* transcription templates and as Primer Extension probes in this work.	This work	Tables S1–S13
Recombinant DNA		
Promoter Fragments inserted in pEXA128 (‘HB’ and ‘BH’ orientations) and used for *in vitro* transcription	Eurofins Genomics	Table S3
L-strand DdCBE monomer constructs cloned into pTracer-CMV/Bsd	This work	N/A
H-strand DdCBE monomer constructs cloned into pcmCherry3.1(-)	This work	N/A
Software and algorithms		
IGVv2.8.4	Robinson et al., 2017	RRID:SCR_011793
Cutadapt v.1.18	Kechin et al., 2017	RRID:SCR_011841
TopHat2 v.2.1.1	Kim etal., 2013	RRID:SCR_013035
Samtools v.1.9	Li et al., 2009	RRID:SCR_002105
Bedtools v.2.28.0	Quinlan and Hall, 2010	RRID:SCR_006646
bowtie2 v2.2.5	Langmead and Salzberg, 2012	RRID:SCR_016368
bigwigCompare v3.1.3	Ramírez et al., 2016	RRID:SCR_016366
Clustal Omega	European Bioinformatics Institute (EBI)	RRID:SCR_001591
MultiGauge version 3.0	Fujifilm	N/A
Graphpad Prism version 8	Graphpad	RRID:SCR_002798
ImageQuant TL 8.2	Cytiva	RRID:SCR_018374

### Resource Availability

#### Lead contact

Further information and requests for resources and reagents should be directed to and will be fulfilled by the lead contact (claes.gustafsson@medkem.gu.se).

#### Materials availability

All unique/stable reagents in this study can be generated using protocols in the Method details section or can be requested from the lead contact.

### Experimental Models and Subject Details

#### Cell lines and cell culture conditions

Human (HeLa (ATCC CCL-2), HEK293 (ATCC CRL-1573), HEK293T (ATCC CRL-3216), SH-SY5Y (CLS 300154)) and chimpanzee (WES, ECACC 91011612) cell lines were grown at 37 °C with 5% (v/v) CO_2_ in Dulbecco’s Modified Eagle Medium (DMEM) supplemented with 10% foetal bovine serum (FBS) and 5% (w/v) penicillin/streptomycin.

### Method Details

#### Proteins

Recombinant human POLRMT, TFB2M, TFAM, and TEFM were expressed and purified according to previously described protocols (Falkenberg et al., 2002; Posse et al., 2015).

#### DNA templates

Linear DNA templates used for *in vitro* transcription and DNase I footprinting assays were generated by PCR using Phusion DNA polymerase (New England Biolabs) from synthetic DNA obtained from Eurofins Genomics in the form of linear dsDNA (GeneStrands), DNA fragments cloned into the multiple cloning site (MCS) of pEX-A128 (Standard Genes), or single-stranded (ssDNA) oligonucleotides. PCR reactions (35 cycles) were supplemented with 3% (v/v) DMSO, and made use of the GC buffer provided by the kit. Details of PCR primers and PCR templates used to generate the linear DNA templates used throughout this work are listed in Table S1. DNase I footprinting templates follow a modified PCR protocol described below. Impurities from PCR reactions, as well as from HindIII linearised plasmid DNA (Figure 6D) were removed using a QIAquick PCR Purification kit (Qiagen), followed by gel extraction of bands of relevant size from TAE agarose gels (40 mM Tris-acetate, 1 mM EDTA, pH 8.0) using a QIAEXii gel extraction kit (Qiagen). All DNA templates were stored in TE buffer (10 mM Tris-HCl, 1 mM EDTA, pH 8.0).

The chimeric template 3′-NCR+HSP was obtained as a synthetic DNA fragment from Eurofins Genomics and was designed to allow transcription from HSP over the 3′-end of the NCR wherein the intermediate sequences between both promoters and the TAS region were reduced, while preserving the strand-usage of HSP. 3′-NCR+HSP was created by fusing nt# 16403–15954 with nt# 610–496 (residues +50 to −63 relative to the TSS of HSP); the order of nucleotides in this notation follows the template schematic shown in Figure 1B. The chimeric template was designed to position HSP 133 bp from the 15 bp ‘core TAS’ region (nt# 16087–16101) from Jemt et al (Jemt et al., 2015). The sequence of 3′-NCR+HSP is provided in Table S3.

DNA templates for promoter mutation experiments in Figures 3, 7B, and S2 were PCR amplified from a series of ssDNA oligonucleotides encompassing −50 to +50 regions of LSP, HSP, or LSP2, and which contain 2 bp (Figures 3E and 3F) or 1 bp (Figures 7B and S2) mutations between −20 and +10 positions (denoted as the LSP+50, HSP+50, and LSP2+50 series, details in Table S1 and sequences listed in Tables S6–S11). Primate mitochondrial promoters used in Figures 5D–5F were also PCR amplified from synthetic ssDNA templates, which encompass −50 to +50 regions of LSP, −64 to +56 regions of HSP, or −94 to +26 regions of LSP2 (based on human mtDNA). The regions equivalent to the human mitochondrial promoters in primate mtDNA were derived from a Clustal Omega sequence alignment (with default parameters) of ape and Neanderthal NCR regions that used tRNA^Phe^ and tRNA^Pro^ as reference points to derive equivalent HSP and LSP2 regions, respectively. GenBank accession numbers and mtDNA coordinates are indicated in Table S12, along with and ssDNA template sequences of human and ape fragments used in this work.

Plasmids carrying fragments corresponding to positions −73 to +90 of LSP, −75 to +90 of HSP, and −74 to +74 of LSP2 (nt# 318–480, nt# 486–1650, and nt# 15956–16103 of the rCRS, respectively) and contained between HindIII and BamHI restriction sites, were ordered from Eurofins Genomics as synthetic fragments cloned into the MCS of pEX-A128 to create pEXA128-LSP(HB), pEXA128-HSP(HB), and pEXA128-LSP2(HB), where ‘**HB**’ refers to the orientation of the HindIII and BamHI sites. These constructs were used to generate linear DNA templates for *in vitro* transcription and DNase I footprinting. The orientation of these promoter fragments in pEXA-128 along with the HindIII and BamHI sites were inverted to create pEXA128-LSP(BH), pEXA128-HSP(BH), and pEXA128-LSP2(BH) which were used in experiments involving negatively supercoiled DNA (Figure 6D). This was done to circumvent the occurrence of a prominent truncated transcript that only occurred when promoter fragments were in the ‘HB’ orientation, which we suspected to occur from transcription termination caused by the structured RNAI sequence at the ColE1 origin of pEXA128 (data not shown). Linear versions of the ‘BH’ constructs in Figure 6D were made by HindIII digestion of plasmids, which cleaves upstream of the promoter fragments. The sequence of the promoter fragments in the ‘HB’ and ‘BH’ orientation in the pEXA128 series can be found in Table S3. Negatively supercoiled plasmid templates used for transcription assays were isolated using a Qiaprep Spin Miniprep kit (Qiagen) from 10 mL cultures of Top10 *Escherichia coli* cells (Invitrogen) transformed with the appropriate plasmids and grown overnight at 37 °C in LB media supplemented with 100 μg/mL ampicillin. Eluted plasmids were stored at 4 °C in TE buffer.

Linear DNA templates for DNase I footprinting experiments (termed LSP-FP, HSP-FP, and LSP2-FP, which carry the promoters indicated) were made by PCR wherein primers corresponding to the template strand were 5′-radiolabelled to uniquely label the template strand of respective substrates. Primers that amplify the template strand of LSP (bt22_aR), HSP (bt22_bR), and LSP2 (bt14_aR) were 5′ end-labelled in a reaction consisting of 33 pmol primer, 3.3 μL γ-^32^P-ATP (3000 Ci/mmol), and 20 U T4 PNK (New England Biolabs) in a final volume of 25 μL, incubating at 37 °C for 1 h followed by heat inactivation at 65 °C for 30 min. The labelled primer was passed through a Microspin™ G25 size-exclusion column (GE Healthcare) to remove unincorporated nucleotides, and 21 μL of the eluate (~25 pmol assuming 90% recovery from the G25 column) was used directly in a 50 μL PCR reaction containing appropriate templates and having ~0.5 μM final concentration of forward and reverse primers. Typically four 50 μL PCR reactions were pooled for footprinting experiments, and were gel-extracted with 30 μL QIAEXii resin, eluting DNA templates twice with 20 μL TE buffer with a 1 min heating step at 50 °C during the 5 min elution step to increase template recovery. The counts per minute (cpm) of labelled DNA was estimated from 1 μL of eluted DNA positioned ~1 cm from the detection plate of a hand-held radiation meter (Contamat FHT 111, Thermo Fisher Scientific). The cpm/μL recorded immediately following gel extraction was used to determine the volume of DNA template required to achieve the 30,000 cpm used for each footprinting reaction, and was kept constant in subsequent experiments without re-measuring the cpm. Unlabelled bt22_aR, bt22_bR, and bt14_aR primers were also used to generate sequencing ladders from their associated DNA templates.

#### *In vitro* transcription

All reactions support multi-round transcription initiation and were prepared on ice prior to the addition of nucleotides. Appropriate DNA templates were mixed with proteins in Transcription Buffer (25 mM Tris-HCl pH 8.0, 10 mM MgCl_2_, 0.1 mg/mL BSA, 1 mM DTT, and 0.16 U/μL RNase Inhibitor (Murine source, New England Biolabs), and appropriate amounts of NaCl (indicated below unless stated in figures). Samples were pre-equilibrated for 2 min at 32 °C, and transcription was initiated by the addition of nucleotides. Reactions were carried out at 32 °C for 30 min and contained 4 nM DNA template (made by PCR, see ‘DNA templates’), 10 nM POLRMT, 20 nM TFB2M, TFAM (adjusted based on the length of the DNA template, see below), 400 μM ATP, 150 μM GTP, 150 μM CTP, 10 μM UTP and 0.027 μM α-^32^P-UTP (3000 Ci/mmol, Hartmann Analytic GmbH), in a final volume of 25 μL. For reactions involving TFAM titrations, TFAM was diluted using its storage buffer (25 mM HEPES-NaOH, 0.2 M NaCl, 1 mM DTT, 10% (v/v) Glycerol, pH 7.2) to normalise the salt contribution from TFAM stocks between reactions. When indicated, TEFM was added to a final concentration of 20 nM (calculated as a dimer). Reactions were stopped by the addition of 200 μL transcription stop buffer (10 mM Tris-HCl, pH 8.0, 0.2 M NaCl, 1 mM EDTA, 0.1 mg/mL glycogen, and 100 μg/mL proteinase K), incubated at 42 °C for 30 min to degrade proteins, ethanol precipitated, and dried at 45 °C under a vacuum using a Vacufuge™ Concentrator (Eppendorf). The resulting pellet was resuspended in 20 μL of 1 × Formamide buffer (25 mM Tris-HCl pH 8.0, 5 mM EDTA, 47.5% (v/v) formamide, 0.025% (w/v) bromophenol blue, 0.025% (w/v) xylene cyanol FF). Products were separated on TBE-urea acrylamide gels of appropriate percentages (90 mM Tris-borate, 2 mM EDTA, and containing 5.2 M (15% acrylamide gel), 6.7 M (8% acrylamide gel), 7 M (6% acrylamide gel), or 7.5 M urea (4% acrylamide gel), and cast using ‘mini’ (28 × 15 × 13 cm) or ‘midi’ (24 × 31 × 17 cm) Reflection™ gel Systems (Gallileo Bioscience). Mini and midi gels were pre-run at 200 V for 1 h without temperature control and electrophoresis was performed at room without temperature control. Gels were exposed without fixing or drying to either storage phosphor screens (Fu-jifilm) at room temperature, or X-ray films (Fujifilm) at −80 °C, in storage cassettes with intensifying screens.

TFAM and NaCl concentrations, when not indicated in figures, are as follows: Figures 1C and 1D (100 nM TFAM, 4 nM NaCl), Figures 2I and 2J (175 nM TFAM, 50 mM NaCl), Figures 3E and 3F (17 nM TFAM, 50 mM NaCl), Figure 4A (25 nM TFAM, 4 mM NaCl), Figures 5D–5F (17 nM TFAM, 50 mM NaCl), Figure 6A (~433 nM TFAM, 50 mM NaCl), Figures S2D–S2I (17 nM TFAM, 50 mM NaCl).

#### Mapping 5′-ends of transcripts by primer extension

RNA templates used in primer extension assays made use of either mitochondrial RNA extracted from human cell lines, or *in vitro* generated RNA from a single 25 μL *in vitro* transcription reaction using appropriate templates. *In vitro* generated RNA was made using standard reaction conditions with 4 mM NaCl but using elevated concentrations of UTP (37 μM) to increase transcript yield and omitting α-^32^P-UTP, handling samples in the same manner as reactions with radioactive UTP. Ethanol-precipitated RNA pellets were dissolved in 8 μL Nuclease-free water (Ambion) supplemented with 1 U/μL RNase Inhibitor. Primers specific to transcripts from appropriate promoters (100 pmol) were end-labelled with 6 μL γ-^32^P-ATP (3000 Ci/mmol, Hartmann Analytic GmbH) and 20 U of T4 PNK in 20 μL, removing unincorporated nucleotides using a Microspin™ G25 column. Unlabelled primers of the same sequence were also used to generate sequencing ladders from the DNA templates used to generate the *in vitro* transcripts in these experiments.

Primer extension reactions were set up on ice. Reactions involving *in vitro* generated RNA made use of 8 μL RNA, 5 μL (~25 pmol) of 5′-radiolabelled primer, and 1 μL (20 U) Transcriptor Reverse Transcriptase (Roche), in a final volume of 20 μL containing components described in the 5′/3′ RACE kit (2^nd^ Generation, Roche), and performed according to kit instructions. For primer extension assays on mitochondrial RNA from human cell lines (Figures 2B, S1E, and S1F), ~4 μg of mitochondrial RNA (in a maximum volume of 8 μL nuclease-free water) was first heat-denatured at 70 °C for 5 min and snap cooled on ice prior to the addition of reaction components, and made use of ~1 pmol labelled primer. To the completed primer extension reactions, an equal volume of 2 × Formamide buffer (95% (v/v) formamide, 10 mM EDTA, 0.05% (w/v) bromophenol blue, 0.05% (w/v) xylene cyanol FF, pH 8.0) was added. Samples were heated at 95 °C for 2 min, and snap cooled on ice for at least 2 min before loading on 10% TBE-urea Sequencing gels (17 × 40 × 0.4 cm Sequi-Gen System, Bio-Rad) containing 6.2 M urea. Sequencing gels were heated to 55 °C by pre-running at 60 W (max. 2 kV) for 1 h, and electrophoresis was performed with temperature control at 55 °C. Following electrophoresis, gels were fixed in Fixing Solution (10% (v/v) methanol, 10% (v/v) acetic acid) for 5 min before being directly exposed to storage phosphor screens (Fujifilm) at room temperature without drying. Images were developed using an FLA-7000 phosphorimager (Fujifilm). Appropriate primers and source RNA used for primer extension experiments in this study are listed in Table S2.

#### DNase I footprinting

DNase I cleavage reactions made use of ~30,000 cpm radiolabelled DNA template in Transcription Buffer supplemented with 1 mM ATP, and the indicated amount of salt and proteins (see below), in a final volume of 20 μL. After a 30 min incubation at 21 °C, 2 μL of 50–100 mU/μL DNaseI (Thermo Fisher Scientific) diluted in 2.5 × DNase I reaction buffer (with MgCl_2_) was added to the samples, and the reaction was mixed by gently tapping the tubes. The dose of DNase I required to achieve the best cleavage was determined empirically for each template preparation. DNase I digestion was quenched by adding 20 μL DNase I stop buffer (200 mM NaCl, 20 mM EDTA pH 8.0, 1% (w/v) SDS, and 1 μg/μL glycogen), vortexed vigorously, and immediately incubated on ice. Footprinting Molecular reactions were extracted with equal volumes of phenol:chloroform:isoamyl alcohol (25:24:1), then with equal volumes of chloroform:isoamyl alcohol (24:1), before the aqueous phase containing DNA was ethanol precipitated in the presence of 0.3 M sodium acetate (pH 5.2). The pellet was spin-dried at 45 °C under vacuum as with ethanol precipitated RNA from *in vitro* transcription reactions and redissolved in 10 μL of 1 × Formamide Buffer (see in vitro transcription). Samples were heat denatured at 95 °C for 2 min and snap-cooled on ice for 2 min prior to separation on 8% TBE-urea Sequencing gels containing 6.7 M urea, which were run, fixed, and imaged in the same way as in Primer Extension experiments. Densitometric analysis of cleavage products is described in Quantification and statistical analysis. The DNase I dose, amount of proteins, and salt concentrations of footprints shown in Figures 4D–4F and S4 are as follows: LSP-FP made use of 150 mU DNase I, 2 pmol TFAM, 4 pmol POLRMT, 4 pmol TFB2M, and 50 mM NaCl; HSP-FP made use of 150 mU DNase I, 3 pmol TFAM, 6 pmol POLRMT, 6 pmol TFB2M, and 65 mM NaCl; LSP2-FP made use of 100 mU DNase I, 3 pmol TFAM, 2 pmol POLRMT, 2 pmol TFB2M, and 65 mM NaCl.

#### Sequencing ladders

Sequencing ladders were generated from non-radioactive PCR-amplified linear DNA templates using the USB® Sequenase™ Version 2.0 DNA polymerase kit (Affymetrix), following kit instructions for ‘generating sequencing ladders closer to the primer’. All reactions were set up on ice, and labelling reactions using α-^32^P-dATP (3000 Ci/mmol, Hartmann Analytic GmbH) used a 1:15 dilution of labelling mix (dGTP), 1:8 dilution of Sequenase, supplemented with 1 μL Mn Buffer, and were performed at 16 °C for 2 min prior to the dideoxy termination step. All sequencing reactions were carried out in thin-walled 200 μL PCR tubes and brought to the required temperatures using aluminium heating blocks. Variations specific to templates used throughout this work are indicated below.

DNA templates for sequencing ladders used alongside primer extension reactions for LSP and LSP2 were chemically denatured in the presence of 0.2 M NaOH and 0.2 mM EDTA as described in the Sequenase kit and recovered by ethanol precipitation, dissolving pellets in nuclease-free water, and assuming 90% sample recovery from this method. The annealing step for these templates was carried out at 65 °C according to kit instructions and used ~2 pmol each of the appropriate 5′-radiolabelled primer and denatured DNA template (primer bt13_aF2 for template LSP+90 and primer bt9_aF2 for template 3′-NCR(no HSP)). Sequencing ladders for primer extension reactions of HSP used 2 pmol of template HSP+90 annealed to 20 pmol of primer bt13_bR2 by heat denaturation at 98 °C for 8 min, followed by snap-cooling the mixture on ice for at least 2 min. The labelling reaction with α-^32^P-dATP for HSP+90 was also modified and carried out for 2 min on ice.

Sequencing ladders used alongside DNase I footprinting experiments were generated using primers bt22_aR for template LSP-FP, bt22_bR for template HSP-FP, and bt14_aR for template LSP2-FP, annealing the primers using the heat denaturation and snap cooling protocol described for HSP+90 (see above). Reactions for template LSP-FP were modified to use double the amount of Sequenase (1:4 dilution instead of 1:8) to improve the read-through beyond a homopolymeric G/C block positioned at the −26 region of LSP.

In all cases, sequencing reactions were stopped by adding equal volumes of 2 × Formamide loading buffer, then heated at 95 °C for 2 min followed by snap cooling on ice for at least 2 min prior to loading on sequencing gels. Typically, 2–5 μL of sequencing reactions were used alongside primer extension experiments, whereas 0.5–1 μL was used alongside DNase I footprints. Sequencing ladders were diluted in 1 × Formamide buffer (see in vitro transcription) to match the volume of samples loaded onto sequencing gels.

#### Size markers for electrophoresis

Primer extension and DNase I footprinting gels made use of a 10–100 bp low molecular weight DNA ladder (Affymetrix), whereas *in vitro* transcription reactions made use of either a low molecular weight DNA ladder (covering 25–766 bp, New England Biolabs), 50 bp Generuler (Thermo Fisher Scientific), or 25 bp DNA step ladder (Promega). Size markers were 5′ end-labelled using T4 PNK and γ-^32^P-ATP (3000 Ci/mmol), and passed through a Microspin™ G25 column to remove unincorporated nucleotides. In particular, both the 50 bp Generuler and the 25 bp DNA step ladder were dephosphorylated with Antarctic Phosphatase (New England Biolabs) prior to 5′ end-labelling. Size markers were diluted in 1 × Formamide buffer (see in vitro transcription) and heat denatured at 98 °C for 3 min prior to loading in TBE-urea PAGE gels.

#### siRNA treatment

For MRPP3 siRNA transfections, cells were reverse transfected with 20 nM of indicated siRNA (Horizon Discovery; MRPP3 siRNA catalogue number: M-020859-01-0050; Non-targeting control siRNA catalogue number: D-001210-01-50) using Lipofectamine RNAiMAX (Thermo Fisher Scientific), and harvested after three days. For dual TOP1MT and TOP3A siRNA transfections, approx. 1 × 10^7^ cells were reverse transfected using Lipofectamine RNAiMAX and 5 nM of each siRNA (Silencer Select siRNA, Ambion). After three days, cells were passaged and re-transfected as above for a further three days. Cells were collected after 6 days of transfection at confluency from one BioAssay dish (Nunc, approx. 600 cm^2^). Oligonucleotides used for siRNA transfection were: TOP1MT (Thermo Fisher Scientific cat. 4392420, Assay ID s42017, PubChem SID 160755427) and TOP3A (Thermo Fisher Scientific cat. 4392420, Assay ID s14310; PubChem SID 160718251).

#### Western blotting

We confirmed effective knockdowns by western blotting. For experiments involving MRPP3 siRNA, cells harvested at 80–90% confluence from a single well of a 6-well plate were lysed by resuspending cell pellets in 50 μL Lysis Buffer A (50 mM Tris-HCl pH 7.4, 150 mM NaCl, 1 mM EDTA, 1% (w/v) Triton X-100, 2 mM benzamidine, 2 mM pepstatin A, 1 mM phenylmethlsulfonylfluoride, 0.5 μM leupeptin). The lysate was incubated on ice for 20 min, followed by centrifugation at 12,000 × g for 10 min at 4 °C. The supernatant was transferred to a new microfuge tube, mixed with equal volumes of 2 × Laemmli sample buffer (Bio-Rad), and heated at 95 °C for 5 min. From this, 20 μL was loaded on a Criterion 4-20% TGX Stain-free SDS-PAGE gel (Bio-Rad) and proteins were then transferred to a nitrocellulose membrane (Amersham Protran Premium, 0.45 μm pore size). Membranes were blocked for 1h at room temperature with PBSTM buffer (PBST supplemented with 5% (w/v) milk: 10 mM Na_2_HPO_4_, 1.8 mM KH_2_PO_4_, pH 7.4, 137 mM NaCl, 2.7 mM KCl, 0.1% (v/v) Tween 20, 5% (w/v) milk powder) and blotted with primary antibodies for 1 h. Membranes were washed 3 times in PBST for 10 min each time, and incubated with appropriate HRP-conjugated secondary antibodies for 1 h, then washed again as with the primary antibody. A SuperSignal™ West Dura Extended Duration Substrate (Thermo Fisher Scientific) kit was used to generate enhanced chemiluminescence from the blotted membranes, which was imaged using a ChemiDoc MP Imaging system (Bio-Rad). Primary antibodies were: rabbit anti-MRPP3 antibody (Abcam, ab185942, diluted 1:1,000) or mouse anti-β-Actin antibody (Abcam, ab6276, diluted 1:5,000) as a loading control; diluted in PBSTM. Secondary antibodies were: HRP sheep anti-mouse IgG (GE Healthcare, NXA931) or HRP donkey anti-rabbit IgG (GE Healthcare, NA9340; diluted 1:10,000 PBSTM).

For experiments using TOP1MT and TOP3A siRNA, protein was extracted from mitochondrial pellets (see *c*rude mitochondrial isolation and mitochondrial RNA preparation) and gels were run as above with the following modifications: primary antibody incubations were carried out overnight at 4 °C without Tween-20, and blots were developed using SuperSignal West Pico Plus (Pierce) and imaged by exposure to X-ray film. Primary antibodies used were: TOP1MT (Proteintech 16540-1-AP, 1:1,000), TOP3A (Proteintech 14525-1-AP, 1:1,000), and VDAC (Abcam ab14734, 1:5,000); secondary antibodies used were: rabbit anti-mouse HRP (Agilent P0260, 1:3,000), and swine anti-rabbit HRP (Agilent P0217, 1:3,000); antibodies diluted in PBSM Buffer (PBSTM without Tween 20).

#### Crude mitochondrial isolation and mitochondrial RNA preparation

For preparations involving cells transfected with MRPP3 siRNA, cells at about 80% confluence were harvested by trypsinisation and centrifuged at 500 × g for 3 min at 4 °C, washed once with cold PBS (10 mM Na_2_HPO_4_, 1.8 mM KH_2_PO_4_, 137 mM NaCl, 2.7 mM KCl), and pelleted again at 500 × g. The cell pellet was resuspended in 1 mL of Lysis Buffer B (20 mM Tris-HCl pH 7.6, 220 mM mannitol, 70 mM sucrose, 1 mM EDTA, 2 mM benzamidine, 2 mM pepstatin A, 1 mM phenylmethlsulfonylfluoride (PMSF), 0.5 μM leupeptin, 1 mg/mL BSA), incubated on ice for 15 min, and transferred to a glass Dounce homogenizer with a 1 mL capacity. Cells were lysed with 20–25 strokes, and the lysate was centrifuged at 1,200 × g for 3 min at 4 °C. The supernatant was transferred to a new microfuge tube and centrifuged at 14000 × g for 3 min at 4 °C to pellet the crude mitochondria. Mitochondrial RNA was isolated from crude mitochondria by TRIzol (Thermo Fisher Scientific) extraction following manufacturer’s instructions and subsequently ethanol precipitated. RNA pellets were dissolved in nuclease-free water and treated with RNase-free DNase I at 37 °C for 20 min, followed by purification using a Quick RNA Micro prep column (Zymo Research). RNA quantity and purity was assessed using a Nanodrop spectrophotometer.

For preparations involving cells transfected with TOP1MT and TOP3A siRNA, HeLa cells (approx. 8 × 10^7^) were collected by trypsinisation, pelleted by centrifugation at 300 × g for 5 min at room temperature, washed with PBS, and pelleted again. Cell pellets were resuspended in 5 mL of Hypotonic Buffer (20 mM HEPES-NaOH (pH 8.0), 5 mM KCl, 1.5 mM MgCl_2_, 2 mM DTT, 1 mg/mL BSA, 1 mM PMSF, and 1 × protease inhibitors (Pierce Protease Inhibitor Tablets, EDTA-free)) and incubated on ice for 10 min. Cells were disrupted using 10 strokes of a glass Dounce homogeniser with a tight-fitting pestle, and then a two-thirds volume of 2.5 × MSH buffer (525 mM mannitol, 175 mM sucrose, 20 mM HEPES-NaOH (pH 8.0), 5 mM EDTA, 1 mg/mL BSA, 0.2 mM PMSF, 2 mM DTT, and 1 × protease inhibitors) was added. The homogenate was centrifuged at 1,600 × g for 10 min at 4 °C to pellet nuclei and unbroken cells, the supernatant was recovered, and this centrifugation step was repeated once more. The supernatant from this step was centrifuged at 10,000 × g for 10 min at 4 °C to pellet crude mitochondria. The mitochondrial fraction was washed twice by resuspension in 2 mL of 1 × MSH (210 mM mannitol, 70 mM sucrose, 20 mM HEPES-NaOH (pH 8.0), 2 mM EDTA, 1 mg/mL BSA, 0.2 mM PMSF, and 1 × protease inhibitors) and pelleted by centrifugation at 10,000 × g for 10 min at 4 °C. A sample of isolated mitochondria was retained for western blotting analysis. RNA was isolated from the remaining mitochondrial pellets using an RNeasy mini kit (Qia-gen) incorporating an on-column DNase digestion step (RNase-Free DNase Set, Qiagen), according to the manufacturer’s instructions. RNA quantity was determined using a Qubit RNA broad range assay kit, and purity was assessed using a Nanodrop spectrophotometer.

For preparations involving WES cells, as well as and HEK293T cells transfected with DdCBE constructs, cells at 70–80% confluence were washed with PBS and resuspended in Hypotonic Buffer, incubated on ice for 10 min and homogenised with a Balch homogeniser with a 12 μm clearance ball bearing. Two-thirds of the total homogenate volume of 2.5 × MSH buffer was immediately added, and differential centrifugation was used to obtain the different cell fractions (1,000 × g for 10 min to remove cell debris and nuclei, and subsequently at 10,000 × g for 10 min to pellet mitochondria). RNA from the crude mitochondrial pellet was the extracted using TRIzol reagent, treated with 2 U Turbo DNase (Thermo Fisher Scientific), followed by clean-up using RNA clean and concentrator column (Zymo) according to the manufacturer’s instructions.

#### Northern blotting of mitochondrial tRNAs

Whole-cell RNA was extracted from either control (expressing catalytically inactive DdCBEs) or LSP2-edited (expressing catalytically active DdCBEs) stable cell lines by treating cell pellets with TRIzol reagent, 2 U Turbo DNase, followed by precipitation with isopropanol. Approximately 10 μg extracted RNA were diluted to uniform volumes in TE buffer and mixed with equal volumes of 2 × Formamide buffer, heat denatured at 98 °C for 3 min, snap cooled on ice for 2 min, and equally split across four 15% urea-PAGE gels (loading ~2.1 μg RNA per lane in each gel) to simultaneously probe multiple tRNAs. PAGE-separated RNA were capillary-transferred from gels onto positively-charged nylon membranes (Hybond N+, Cytiva) overnight and cross-linked to membranes by exposure to 254 nm UV (200 mJ/cm^2^).

Single-stranded DNA probes freshly 5′-radiolabelled with γ-^32^P-ATP and T4 PNK (10 pmol probe, 2 μL γ-^32^P-ATP (3000 Ci/mmol), 20 U T4 PNK in 25 μL) were purified using a G25 column and diluted with 200 μL PerfectHyb™ PLUS hybridisation buffer (Sigma-Aldrich). Diluted probes were then added dropwise into hybridisation tubes containing sample membranes that have been equilibrated with 10 mL PerfectHyb™ PLUS buffer for 30 min at 45 °C in a hybridisation oven (without decanting the equilibration buffer). Hybridisation was performed for 4 h at 45 °C. Probe solutions were decanted and membranes were then washed 3 times in hybridisation tubes with 2 × SSC Buffer (300 mM NaCl, 30 mM sodium citrate) supplemented with 0.1% (w/v) SDS, with wash steps performed at 45 °C in a hybridisation oven for 15 min. Washed membranes were transferred to heat-sealed plastic (polyethylene film, 50 μm) pouches to prevent drying, and imaged by phosphorimaging after an overnight exposure to storage phosphor screens. Probes were stripped from membranes by treatment with a boiling solution of 0.1% (w/v) SDS (preheated to 100 °C) for 15 min, for a total of 3 times. Stripped membranes were washed 5 times with warm (40–50 °C) ultrapure water (autoclaved) to remove traces of SDS, and re-probed as described above. Hybridisation tubes and all equipment used in handling membranes were treated with RNaseZap™ (Ambion) prior to use. Quantification of northern blots is described in data and image analysis. Probes used were: tRNA^Pro^: bt32_tRNAPro; tRNA^Glu^: bt32_tRNAGlu; tRNA^Tyr^: bt32_tRNATyr; tRNA^Phe^: tRNAPhe_probe; tRNA^Leu1^: tRNALeu1_probe; tRNA^Trp^: tRNATrp_probe; tRNA^Lys^: tRNALys_probe; 5.8S RNA: 5.8SRNA_probe; sequences in Table S13.

#### Base-editing of mtDNA using DdCBEs

We screened for viable mtDdCBE monomer constructs from permutations of L- or H-strand binding TALE arrays (3 for each strand) fused with either N- or C-terminal halves of the ‘G1333’ or ‘G1397’ DddA_tox_ splits reported by Mok et al. (Mok et al., 2020) (Figure S6). TALE arrays were designed using Repeat Variable Diresidues (RVDs) containing NI, NG, NN and HD (1-letter amino acid abbreviations), recognizing A, T, G and C nucleobases, respectively. To construct the plasmids used in the cell screen, all DdCBE ORFs were synthesized as gene blocks (GeneArt, Thermo Fisher Scientific). DdCBE monomers binding the L-strand (L-strand monomer) of human mtDNA were cloned into 5′-NotI and 3′-XbaI sites of pTracer-CMV/Bsd (Life Technologies), which contains GFP as a fluorescent reporter. H-strand binding monomers (H-strand monomer) were cloned into 5′-NotI and 3′-KpnI sites of pcmCherry3.1(−), which contains mCherry as a fluorescent reporter (Gammage et al., 2016).

For DdCBE pair screens, HEK293T cells plated in 6-well tissue culture plates at a 70–80% confluence were transfected with 3.2 μg of each monomer (L- and H-monomers), using 16 μl of FuGENE-HD (Promega), following manufacturer’s guidelines. After 24 h, cells were collected for fluorescence-activated cell sorting (FACS) and sorted for GFP and mCherry double-positive cells using a BD FACSMelody™ cell sorter. The collected double-positive cells were allowed to recover for another 6 days (in DMEM with Glutamax) and subsequently used for DNA extraction and Sanger sequencing to assess the levels of base editing. In order to increase the heteroplasmy of these modifications, we subjected wild-type (WT) HEK293T cells to three rounds of transfection with the selected mtDdCBE pairs followed by a 14-day recovery after each delivery. The amino acid sequences of the DdCBE pairs used to edit LSP2 in Figure 7 are listed in Table S14. In total, we screened 36 combinations of L- and H-strand mtDdCBE monomer pairs transfected into HEK293T cells, and selected the pairs with highest base-editing for each position, which precisely targeted the −1 and +4 positions of LSP2, respectively.

For the generation of stable cell lines expressing DdCBEs, Flp-In™-HEK293T human cells in conjunction with the Flp recombinase expression plasmid (pOG44) were used following manufacturer’s guidelines. After 24 h, the cells were cultured in medium supplemented with 200 μg/mL hygromycin B (Thermo Fisher). Cells resistant to hygromycin B expressing DdCBEs were expanded for further analysis.

Genomic DNA for Sanger sequencing was extracted by resuspending HEK293T human cells Lysis Buffer C (50 mM Tris-HCl, pH 8.0, 1 mM EDTA, 1% (v/v) Tween 20, 200 μg/mL proteinase K), incubating lysates first at 56 °C for 1 h, then at 95 °C for 10 min, before use in PCR. The LSP2 base-edited region was PCR-amplified with Phusion High-Fidelity PCR Master Mix (New England Biolabs) using primers LSP2_Fw and LSP2_Rv (see Table S13). PCR was performed with an initial heating step of 30 s at 98 °C followed by 35 cycles of amplification (10 s at 98 °C, 20 s at 58 °C, 30 s at 72 °C), and a final step of 7 min at 72 °C. PCR purification and Sanger sequencing were carried out by GENEWIZ (UK) using the LSP2_Fw primer.

#### Cappable-seq library preparation and analysis

Mitochondrial RNA isolated from HeLa, WES, and HEK293T cells were sent for both Cappable-seq (Ettwiller et al., 2016) and TagRNA-seq (Innocenti et al., 2015) services provided by Vertis Biotechnologie (Freising, Germany) according to the specifications required by the company. Cappable-seq allows the enrichment of the 5′-ends of primary transcripts whereas TagRNA-seq allows the identification of the 5′-ends of both primary and processed transcripts, and thus we used the TagRNA-seq reads to filter out ribo-somal RNA contamination in Cappable-Seq reads (described below). Library preparation, sequencing, and adapter trimming of TagRNA-seq reads were performed by Vertis. Single-end sequencing reads from Cappable-seq were trimmed off adapter sequences with cutadapt v1.18 (Kechin et al., 2017) (-a AGATCGGAAGAGCACACGTCTGAACTCCAGTCAC –m 10).

Trimmed reads were mapped to the human transcriptome (human genome from ftp://ftp.ensembl.org/pub/release102/fasta/homo_sapiens/dna/Homo_sapiens.GRCh38.dna.primary_assembly.fa.gz, and annotations from ftp://ftp.ensembl.org/pub/release-102/gtf/homo_sapiens/Homo_sapiens.GRCh38.102.gtf.gz) or chimpanzee transcriptome (chimpanzee genome from http://ftp.ensembl.org/pub/release-104/fasta/pan_troglodytes/dna/Pan_troglodytes.Pan_tro_3.0.dna_sm.toplevel.fa.gz and annotations from http://ftp.ensembl.org/pub/release-104/gtf/pan_troglodytes/Pan_troglodytes.Pan_tro_3.0.104.gtf.gz) using TopHat2 v2.1.1 (Kim et al., 2013) under default parameters. Reads mapped to the mitochondrial genome and reads that remained unmapped were extracted and converted to a concatenated FASTQ file using samtools v1.9 (Li et al., 2009). The FASTQ file was realigned to the mitochondrial genome using bowtie2 v2.2.5 (Langmead and Salzberg, 2012) (–local), and eventually about 50% of total initial Cappable-seq reads and 10% of total initial TagRNA-seq reads (~10 million per sample) were mapped to the mitochondrial genome. 5′-end extraction was performed with samtools and bedtools v2.28.0 (Quinlan and Hall, 2010). The 5′-ends obtained from Cappable-seq and non-enriched total 5′-ends obtained from TagRNA-seq were normalized to total mitochondrial reads from each library as reads per million mapped reads (‘5capRPM’ for Cappable-seq and ‘5tagRPM’ for TagRNA-seq), plus a pseudocount of one. The mean of two Cappable-seq datasets were visualized in 5capRPM using IGV v2.8.4 (Robinson et al., 2017). To remove the ribosomal RNA contamination, the ratio of 5capRPM/5tagRPM was calculated using bigwigCompare v3.1.3 (Ramírez et al., 2016) with a 1 bp window size and the positions with ratio below 1 were filtered out in our analysis.

#### Bioinformatic analysis of mtDNA mutations from human cohorts

Human cohort data of mutations at LSP, HSP and LSP2 in Figures S2J–S2L were analysed as described in previous work (Turro et al., 2020; Wei et al., 2017, 2019). Homoplasmic mtDNA mutations were re-analysed from 30,506 mtDNA sequences from across the globe, derived from GenBank (Wei et al., 2017), where only the major allele was analysed.

### Quantification and Statistical Analysis

Storage phosphor screens were scanned using a FLA-7000 phosphorimager (version 1.12). X-ray films were developed using an XR24-NDT X-ray film processor (DU¨ RR NDT GmbH) and scanned using a desktop scanner. The 16-bit densitometric scans from phosphorimaging, as well as scanned X-ray films, were analysed using MultiGauge software (version 3.0, Fujifilm). Graphs were plotted using Graphpad Prism version 8. Representative gel images were taken from at least two independent repeats unless otherwise stated.

Differential cleavage plots of DNase I footprinting experiments (Figure S4) were derived from densitometric scans of sequencing gels from phosphorimaging, and analysed using the 1D gel analysis software of ImageQuant TL 8.2 (Cytiva). Molecular weight assignments for DNase I cleavage products were assigned by converting pixel position to molecular weight (nt) using the bands from the T-ladder sequencing lane (ranges from 42–176 nt for LSP2-FP, 39–147 nt for LSP-FP, 45–148 nt for HSP-FP) that were fit to a first order Lagrange curve provided by the software. Calibrated lane profiles from ImageQuant were exported to Microsoft Excel. From this dataset, the pixel density values of band sizes closest to the whole numbers used in the analysed size range (60–140 nt, corresponding to +15 to −65 relative to each promoter) were taken and used to calculate the fractional cleavage at each nucleotide. Fractional cleavage (*F*) was calculated as [*F* = *D*_*nt*_/*D*_*T*_] where *D*_*nt*_ is the pixel density at each nucleotide position relative to *D*_*T*_, the total lane density. Values of *F* for each lane were smoothened by taking running averages of 3 nt, calculated by taking the average density at a given position with that of its ± 1 nt neighbours. The smoothened dataset was used to generate differential cleavage plots. Differential cleavage (*D*) at each nucleotide was calculated as [*D* = log_10_(*F*_*p*_/*F*_*c*_)] where *F*_*p*_/*F*_*c*_ is the ratio of the fractional cleavage at a given position in the presence of a particular protein cocktail (*F*_*p*_) relative to the fractional cleavage at the same position in the “no protein” control lane (*F*_*c*_). Positive values of *D* indicate increased cleavage at a particular position, whereas negative values of *D* indicate decreased cleavage (protection), relative to the no protein control.

For the quantification of northern blots in Figure 7O, the pixel density of tRNA bands were first normalised to the pixel density of the membrane-specific loading control (nuclear-encoded 5.8S RNA) for each lane (see Figure S7G), to account for variations in loading, after subtracting a ‘background’ signal taken from the bottom-most part of each lane, for each blot, using identical ‘box’ dimensions to the probed RNA bands. This ‘load-normalised’ pixel density (*d*_*n*_) was calculated as [*d*_*n*_ = *d*_*tRNA*_/*d*_*5.8S*_], where *d*_*tRNA*_ is the pixel density of the probed tRNA band, and *d*_*5.8S*_ is the pixel density of the membrane-specific 5.8S RNA band for the lane in which *d*_*tRNA*_ was taken. The densitometric *d*_*n*_ values for each replicate (including those of the control cell group expressing inactive mtDdCBEs) were then converted to “fold relative to control” values (*d*_*n(fold)*_), calculated as [*d*_*n(fold)*_ = *d*_*n(rep)*_/*d*_*n(mc)*_], where *d*_*n(rep)*_ is the *d*_*n*_ of each replicate, and *d*_*n*(*mc*)_ is the mean *d*_*n*_ value of the control cell group. This conversion allows us to generate datasets that report both the fold changes in tRNA levels of LSP2-edited cells relative to control cells, as well as the degree of variation in the control cell group. The *d*_*n(fold)*_ values for individual tRNAs from biological triplicates were used to calculate significant differences between control and LSP-edited cells using a one-tailed unpaired Student’s t-test (p ≤ 0.05) in Graphpad Prism v8.0. We note that Student’s t-tests conducted on both *d*_*n*_ (the ‘raw’ data normalised to loading control) and *d*_*n(fold)*_ (the ‘fold-normalised’ data) gave identical p-values. Pixel densities were obtained from phosphor images using ImageQuant TL 8.1.

## Supplementary Material

**Supplemental Information**

Supplemental information can be found online at https://doi.org/10.1016/j.molcel.2022.08.011.

Supplemental information

## Data Availability

Cappable-seq and TagRNA-Seq datasets have been deposited in the Gene Expression Omnibus (GEO) on NCBI (accession number: GSE176046) and are publicly available as of the date of publication. This accession number is listed in the key resources table. Expanded sequence alignments of primate mitochondrial promoter regions, as well as uncropped images of northern blots, gels used in promoter mutagenesis, and sequencing gels used in primer extension and DNase I footprinting experiments have been deposited in Mendeley Data (https://doi.org/10.17632/s5434tzz78.1) and are publicly available as of the date of publication. This DOI is listed in the key resources table. This paper does not report original code. Any additional information required to reanalyse the data reported in this paper is available from the lead contact upon request.

## Highlights

- Human mitochondrial DNA (mtDNA) contains an additional light strand promoter (LSP2)
- LSP2 contains identical molecular properties to canonical promoters LSP and HSP
- Base edits to LSP2 affect mitochondrial gene transcript levels in living cells

## In brief

Mitochondrial DNA replication primers and light strand gene products were both thought to arise via transcription from a single promoter (LSP) in humans for over 40 years. Tan et al. report the discovery of an additional promoter (LSP2) on the human mitochondrial genome.
