# Supplementary material for: The human mitochondrial genome contains a second light strand promoter
Source: Mol Cell. Author manuscript; Available in PMC 2026 Sep 1. (PMC7619432; doi:10.1016/j.molcel.2022.08.011)
Supplement: Supplementary file 1 [file EMS217956-supplement-Supplemental_information.pdf]

Molecular Cell, Volume *82*

Supplemental information

The human mitochondrial genome contains a second light strand promoter

Benedict G. Tan, Christian D. Mutti, Yonghong Shi, Xie Xie, Xuefeng Zhu, Pedro Silva-Pinheiro, Katja E. Menger, Héctor Díaz-Maldonado, Wei Wei, Thomas J. Nicholls, Patrick F. Chinnery, Michal Minczuk, Maria Falkenberg, and Claes M. Gustafsson

The human mitochondrial genome contains a second light strand promoter

Benedict G. Tan[1], Christian D. Mutti[2,5], Yonghong Shi[1,5], Xie Xie[1], Xuefeng Zhu[1], Pedro Silva-Pinheiro[2], Katja E. Menger[3], Héctor Díaz-Maldonado[1], Wei Wei[2,4], Thomas J. Nicholls[3], Patrick F. Chinnery[2,4], Michal Minczuk[2*], Maria Falkenberg[1*], and Claes M. Gustafsson[1,6*]

Affiliations

[1] Department of Medical Biochemistry and Cell Biology, University of Gothenburg; Gothenburg 405 30, Sweden.

[2] Medical Research Council Mitochondrial Biology Unit, University of Cambridge; Cambridge CB2 0XY, UK

[3] Wellcome Centre for Mitochondrial Research, Biosciences Institute, Newcastle University; Newcastle upon Tyne NE2 4HH, UK

[4] Department of Clinical Neurosciences, School of Clinical Medicine, University of Cambridge; Cambridge, UK

[5] These authors contributed equally

[6] Lead Contact

* Corresponding authors: michal.minczuk@mrc-mbu.cam.ac.uk, maria.falkenberg@medkem.gu.se, claes.gustafsson@medkem.gu.se

Supplementary Figures S1–S7

Figure S1

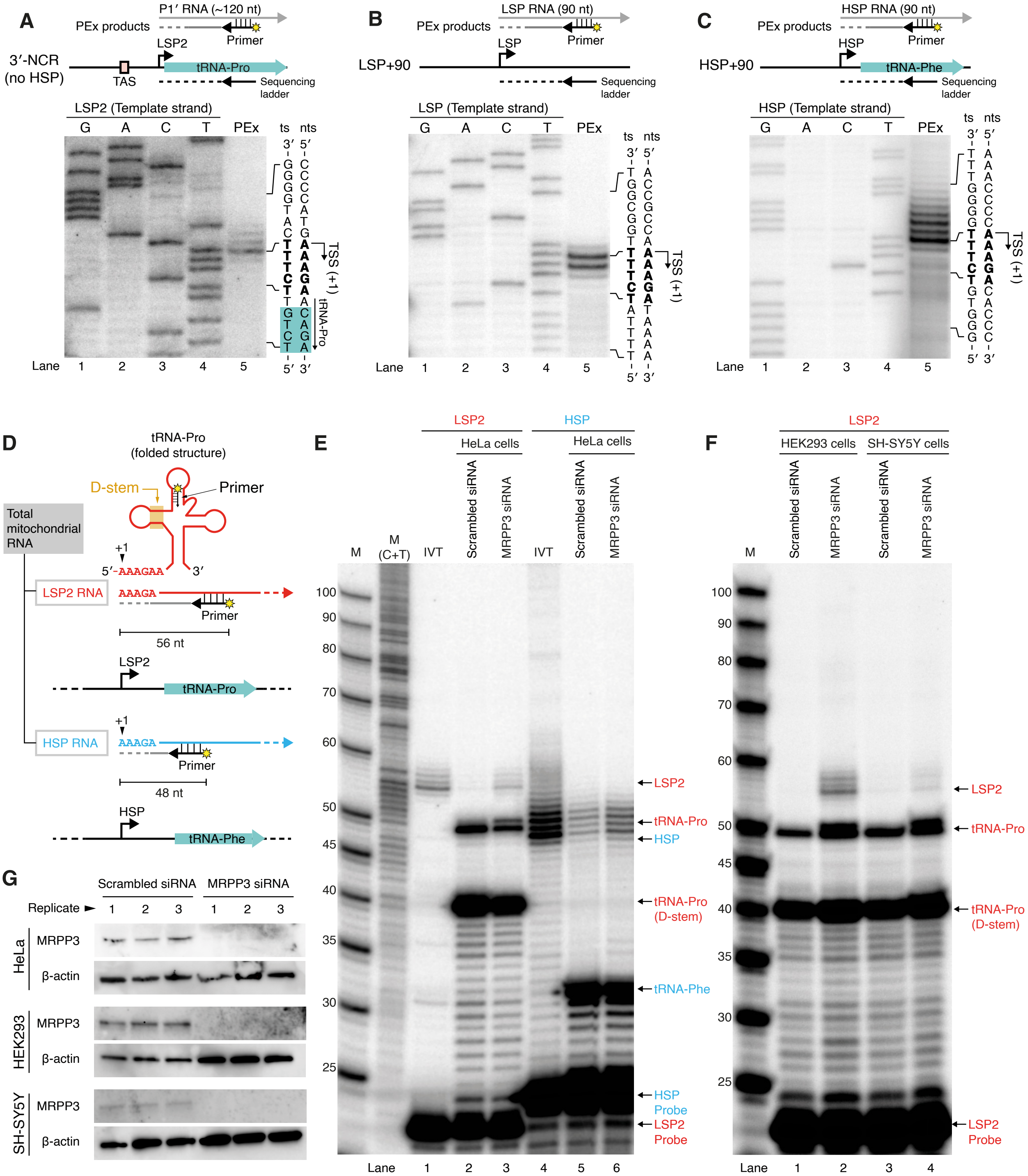
A
PEx products
P1′ RNA (~120 nt)
Primer
LSP2
3′-NCR
(no HSP)
TAS
tRNA-Pro
Sequencing ladder
LSP2 (Template strand)
G A C T PEx
ts nts
TSS (+1)
Lane 1 2 3 4 5
B
PEx products
LSP RNA (90 nt)
Primer
LSP
LSP+90
Sequencing ladder
LSP (Template strand)
G A C T PEx
Lane 1 2 3 4 5
C
PEx products
HSP RNA (90 nt)
Primer
HSP
HSP+90
tRNA-Phe
Sequencing ladder
HSP (Template strand)
G A C T PEx
Lane 1 2 3 4 5
D
tRNA-Pro
(folded structure)
D-stem
Primer
Total mitochondrial RNA
5′-AAAGAA 3′
LSP2 RNA
AAAGA
56 nt
LSP2
tRNA-Pro
HSP RNA
AAAGA
48 nt
HSP
tRNA-Phe
E
LSP2
HSP
HeLa cells
HeLa cells
M
M (C+T)
IVT
Scrambled siRNA
MRPP3 siRNA
IVT
Scrambled siRNA
MRPP3 siRNA
LSP2
tRNA-Pro
HSP
tRNA-Pro (D-stem)
tRNA-Phe
HSP Probe
LSP2 Probe
Lane 1 2 3 4 5 6
F
LSP2
HEK293 cells
SH-SY5Y cells
M
Scrambled siRNA
MRPP3 siRNA
Scrambled siRNA
MRPP3 siRNA
LSP2
tRNA-Pro
tRNA-Pro (D-stem)
LSP2 Probe
Lane 1 2 3 4
G
Scrambled siRNA
MRPP3 siRNA
Replicate 1 2 3 1 2 3
HeLa
MRPP3
β-actin
HEK293
MRPP3
β-actin
SH-SY5Y
MRPP3
β-actin

Figure S1. Detection of LSP2 in different human cell lines. Related to Figure 2.

(A–C) Primer extension (PEx) products of *in vitro* transcripts generated from linear DNA templates containing **(A)** LSP2, **(B)** LSP, or **(C)** HSP, separated on 10% urea-PAGE sequencing gels. DNA templates in (B) and (C) were also used in **Figure 4A,B**. Reverse transcriptase was used to extend 5′-radiolabelled primers (radiolabel: yellow star) annealed to non-radioactive *in vitro* generated RNA, as shown in a schematic above each gel image. PEx products were separated alongside sequencing ladders to provide the base identity of PEx products (sequences of template (ts) and non-template (nts) strands are indicated). **(D)** Schematic of primer extension reactions mapping LSP2 on mitochondrial RNA from human cell lines. The cloverleaf structure of $tRNA^{Pro}$ is shown to highlight the D-stem region, at which the ~40 nt primer extension products of LSP2 transcripts in (B) and (C) are mapped to. This is most likely a result of nucleolytic processing. Total mitochondrial RNA was used as a template for reverse transcriptase primer extension assays using 5′-radiolabelled primer probes (radiolabel: yellow star). Regions of mtDNA encompassing LSP2 and HSP are shown, indicating the predicted sizes (nt) of primer extension products mapping to the +1 sites of either promoter. **(E)** siRNA depletion of MRPP3 enriches for 5′-ends of LSP2 in HeLa cells. Products of primer extension described in (A), from *in vitro* generated RNA ('IVT'; from either 3′-NCR+HSP for LSP2 (panel A), or HSP+90 for HSP (panel C)), or RNA from HeLa cells treated with either a scrambled siRNA (control) or MRPP3 siRNA. Products were separated on a 10% urea-PAGE sequencing gel. Arrows identifying the 5′-ends of LSP2 or HSP indicate the TSS (+1) position. HSP was used as a positive control to illustrate the enrichment of 5′-ends following MRPP3 depletion. Products corresponding to processed 5′-ends mapping to tRNA products of LSP2 ($tRNA^{Pro}$) or HSP ($tRNA^{Phe}$) are also indicated. A combination of C and T sequencing ladders used (A) ('M C+T') was used to provide 1 nt size markers for more accurate mapping of the expected 5′-termini of LSP2 and HSP between the 50 and 60 nt size markers. **(F)** Mapping LSP2 in other human cell lines. Primer extension reactions for LSP2 as in (B) were performed on HEK293 and SH-SY5Y cells. **(G)** Western blots illustrating effective depletion of MRPP3 in the cell lines used in (E) and (F). β-actin was used as a loading control.

Figure S2

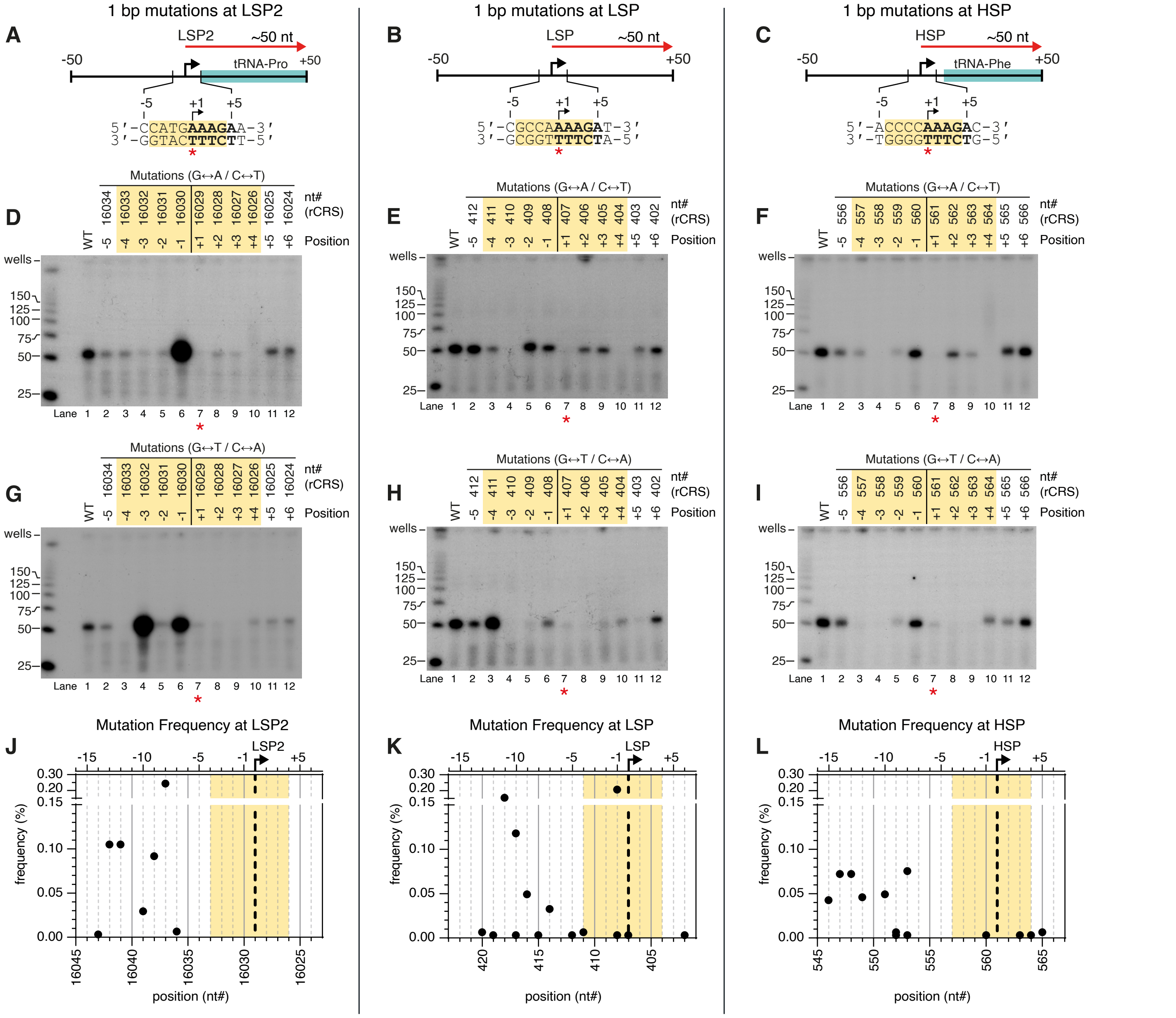

1 bp mutations at LSP2
1 bp mutations at LSP
1 bp mutations at HSP
LSP2
LSP
HSP
~50 nt
tRNA-Pro
tRNA-Phe
5′-CCATGAAAGAA-3′
3′-GGTACTTTCTT-5′
5′-CGCCAAAAGAT-3′
3′-GCGGTTTTCTA-5′
5′-ACCCCAAAGAC-3′
3′-TGGGGTTTCTG-5′
Mutations (G↔A / C↔T)
Mutations (G↔T / C↔A)
nt# (rCRS)
Position
wells
Lane
Mutation Frequency at LSP2
Mutation Frequency at LSP
Mutation Frequency at HSP
frequency (%)
position (nt#)

Figure S2. Genetic variation at the LSP2 locus in humans. Related to Figure 5 and Figure 7.

(A–C) Schematic of DNA templates containing −50 to +50 regions relative to the mapped TSS of (A) LSP2, (B) LSP or (C) HSP, where transcription is expected to produce a 50 nt product (red arrows). Sequences of the −5 to +6 regions of each promoter are shown to highlight the effect of **(D–F)** 1 bp transition (G↔A/C↔T) or **(G–I)** 1 bp transversion (G↔T/C↔A) point mutations at this region on *in vitro* transcription. The sequences of the fragments used are listed in **Table S11** (for LSP2), **Table S13** (for LSP), and **Table S15** (for HSP). Products were separated on 8% urea-PAGE mini gels. The consensus TSS motif common to all human mitochondrial promoters (5′-AAAGA-3′) is indicated in bold, and the +1 position is indicated by a red asterisk. The increase in transcription products observed in the −1G→A and −1G→T mutations to LSP2 (lane 6 in panels D and G) are consistent with the effects of 2 bp mutations to −1/−2 positions observed in **Figure 3E,F**. **(J–L)** Mutation frequency of loci corresponding to (J) LSP2, (K) LSP, or (L) HSP from a re-analysis of data from human cohorts(Turro et al., 2020; Wei et al., 2017, 2019) (see *Materials and Methods*), showing the relatively low frequencies (<0.3%) of homoplasmic mtDNA variations (i.e., only the major allele is considered). The TSS and direction of transcription from each promoter locus is indicated by a bent arrow above the plots.

Figure S3

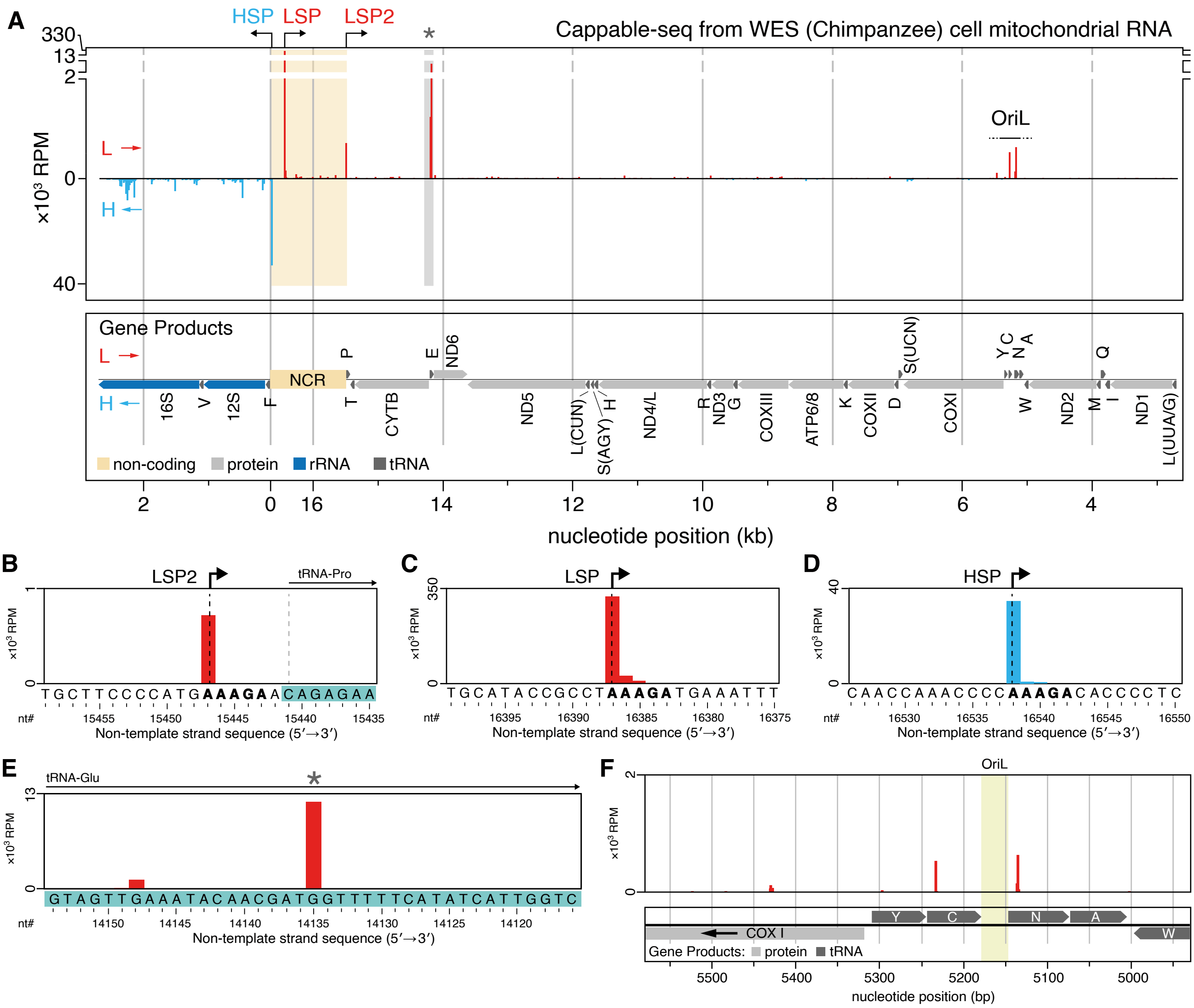

A
HSP LSP LSP2
Cappable-seq from WES (Chimpanzee) cell mitochondrial RNA
×10³ RPM
L
H
OriL
Gene Products
NCR
non-coding
protein
rRNA
tRNA
nucleotide position (kb)
B
LSP2
tRNA-Pro
Non-template strand sequence (5′→3′)
C
LSP
Non-template strand sequence (5′→3′)
D
HSP
Non-template strand sequence (5′→3′)
E
tRNA-Glu
Non-template strand sequence (5′→3′)
F
OriL
COX I
Gene Products: protein tRNA
nucleotide position (bp)

Figure S3. Cappable-seq profiles of WES (chimpanzee) cells. Related to Figure 5.

(A) Global Cappable-seq profiles of 5′-triphosphorylated mitochondrial RNA termini (reads per million, RPM) from the L-strand (red peaks) and H-strand (blue peaks), corresponding to TSS sites from WES (chimpanzee) cell mtDNA. 'L' and 'H' labels with arrows indicate the direction of transcription from either strand, and are aligned to L- and H-strand gene products in the lower panel. An additional prominent peak on the L-strand at $tRNA^{Glu}$ is highlighted in grey and marked with a grey asterisk. Additionally, peaks on the L-strand at the $tRNA^{Tyr/Cys/Asn/Ala/Trp}$ locus occur proximal to the origin of L-strand mtDNA replication (OriL); note that minor peaks are also present in this region in human profiles (**Figure 2D**). **(B–D)** Magnified view of the LSP2, LSP and HSP loci from (A). **(E)** Magnified view of L-strand peaks at the $tRNA^{Glu}$ locus. **(F)** Magnified view of L-strand peaks at the OriL locus.

Figure S4

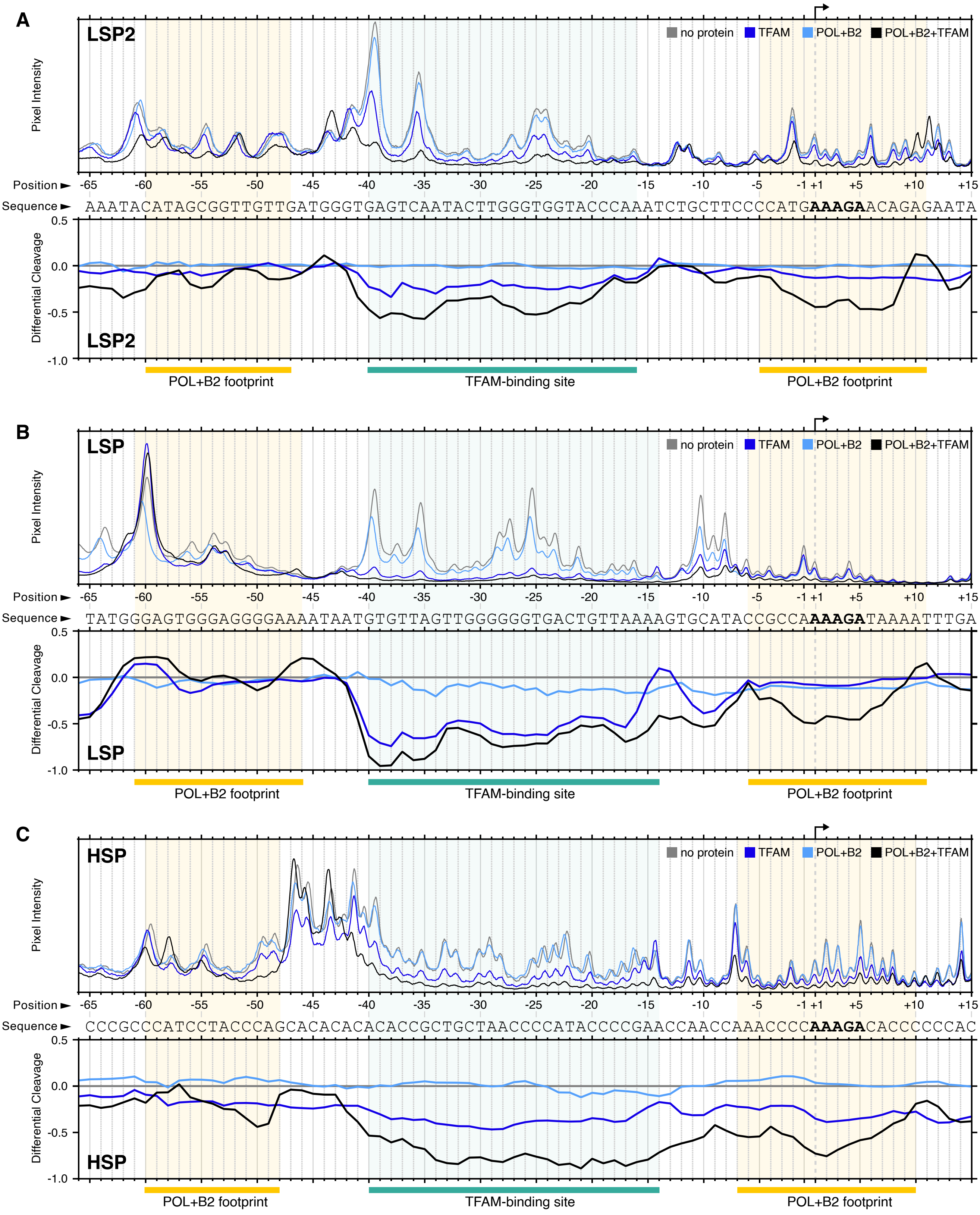
A
LSP2
Pixel Intensity
no protein
TFAM
POL+B2
POL+B2+TFAM
Position ►
-65 -60 -55 -50 -45 -40 -35 -30 -25 -20 -15 -10 -5 -1 +1 +5 +10 +15
Sequence ►
AAATACATAGCGGTTGTTGATGGGTGAGTCAATACTTGGGTGGTACCCAAATCTGCTTCCCCATGAAAGAACAGAGAATA
Differential Cleavage
0.5
0.0
-0.5
-1.0
LSP2
POL+B2 footprint
TFAM-binding site
POL+B2 footprint
B
LSP
Pixel Intensity
no protein
TFAM
POL+B2
POL+B2+TFAM
Position ►
-65 -60 -55 -50 -45 -40 -35 -30 -25 -20 -15 -10 -5 -1 +1 +5 +10 +15
Sequence ►
TATGGGAGTGGGAGGGGAAAATAATGTGTTAGTTGGGGGGTGACTGTTAAAAGTGCATACCGCCAAAAGATAAAATTTGA
Differential Cleavage
0.5
0.0
-0.5
-1.0
LSP
POL+B2 footprint
TFAM-binding site
POL+B2 footprint
C
HSP
Pixel Intensity
no protein
TFAM
POL+B2
POL+B2+TFAM
Position ►
-65 -60 -55 -50 -45 -40 -35 -30 -25 -20 -15 -10 -5 -1 +1 +5 +10 +15
Sequence ►
CCCGCCCATCCTACCCAGCACACACACACCGCTGCTAACCCCATACCCCGAACCAACCAAACCCCAAAGACACCCCCCAC
Differential Cleavage
0.5
0.0
-0.5
-1.0
HSP
POL+B2 footprint
TFAM-binding site
POL+B2 footprint

Figure S4. Densitometry of DNase I footprints. Related to Figure 4.

Analysis of DNase I cleavage profiles for **(A)** LSP2, **(B)** LSP, and **(C)** HSP. Upper plots on each panel show density profiles of DNase I footprinting gels from **Figure 4**. Lower plots on each panel show differential cleavage derived from density profiles of each promoter (see *Materials and Methods*), where negative values indicate less cleavage at a particular nucleotide, and positive values indicate increased cleavage. The indicated DNA sequences in each panel are of the non-template strand (5′→3′ direction), aligned to both density and differential cleavage plots, with TSS motifs indicated in bold text. The respective footprints of either TFAM or POLRMT ('POL') and TFB2M ('B2') for each data set are indicated accordingly below the cleavage plots.

Figure S5

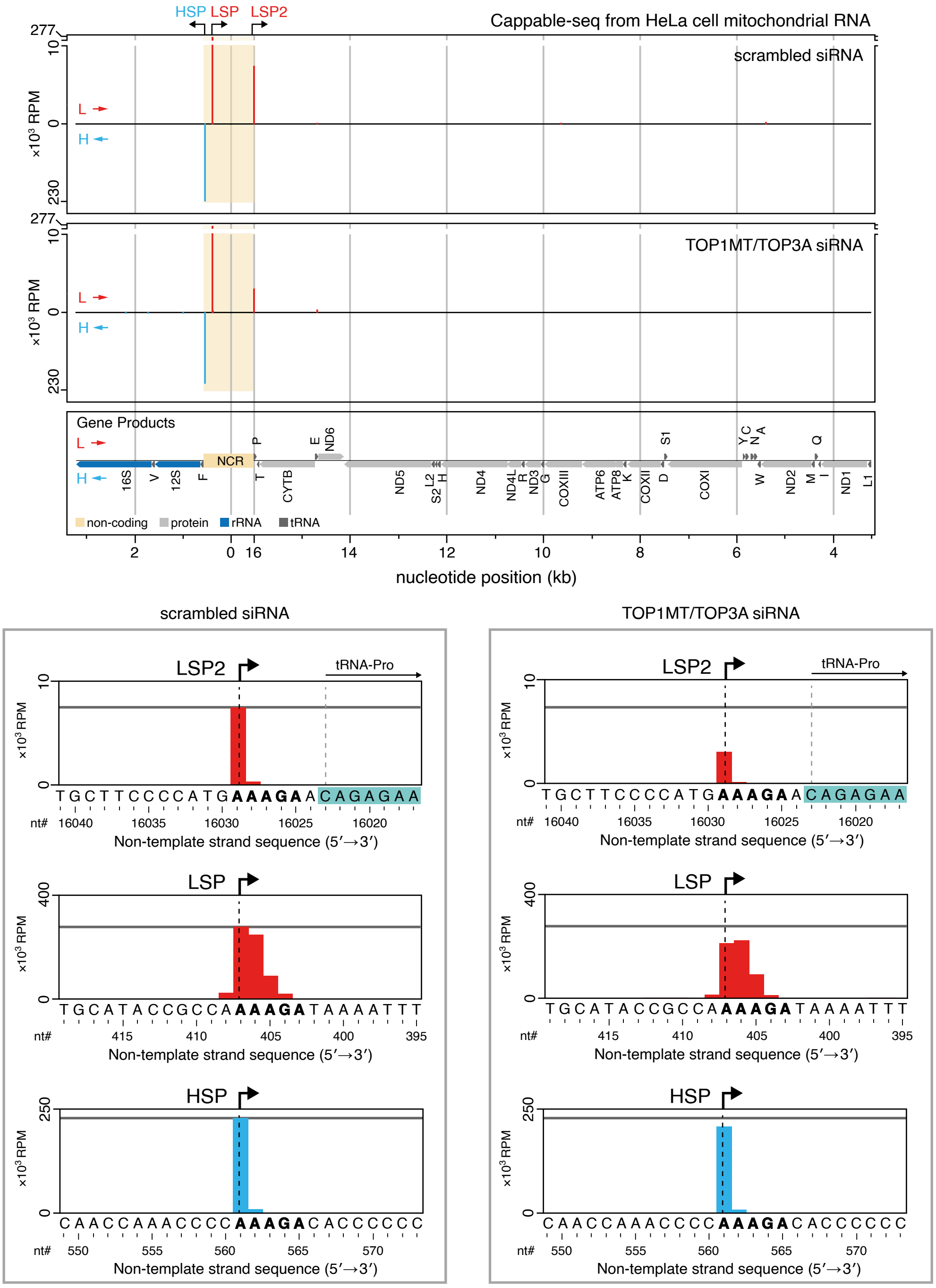

HSP LSP LSP2
Cappable-seq from HeLa cell mitochondrial RNA
scrambled siRNA
TOP1MT/TOP3A siRNA
×10³ RPM
L
H
Gene Products
NCR
non-coding
protein
rRNA
tRNA
nucleotide position (kb)
LSP2
tRNA-Pro
LSP
HSP
T G C T T C C C C A T G A A A G A A C A G A G A A
T G C A T A C C G C C A A A A G A T A A A A T T T
C A A C C A A A C C C C A A A G A C A C C C C C C
nt#
Non-template strand sequence (5′→3′)

Figure S5. Global Cappable-seq profiles of control and TOP1MT/TOP3A depleted cells. Related to Figure 6.

Top panel: Global Cappable-seq profiles of 5-triphosphorylated RNA termini (reads per million, RPM) from the L-strand (red peaks) and H-strand (blue peaks), corresponding to TSS sites from control HeLa cells treated with a scrambled siRNA control, or with TOP1MT and TOP3A siRNA to deplete TOPMT and TOP3A. 'L' and 'H' labels with arrows indicate the direction of transcription from either strand, and are aligned to L- and H-strand gene products in the lower panel. Bottom panels: Magnified Cappable-seq profiles at LSP2, LSP, and HSP, presented for ease of comparison. Horizontal grey lines in indicate peak heights from control profiles.

Figure S6

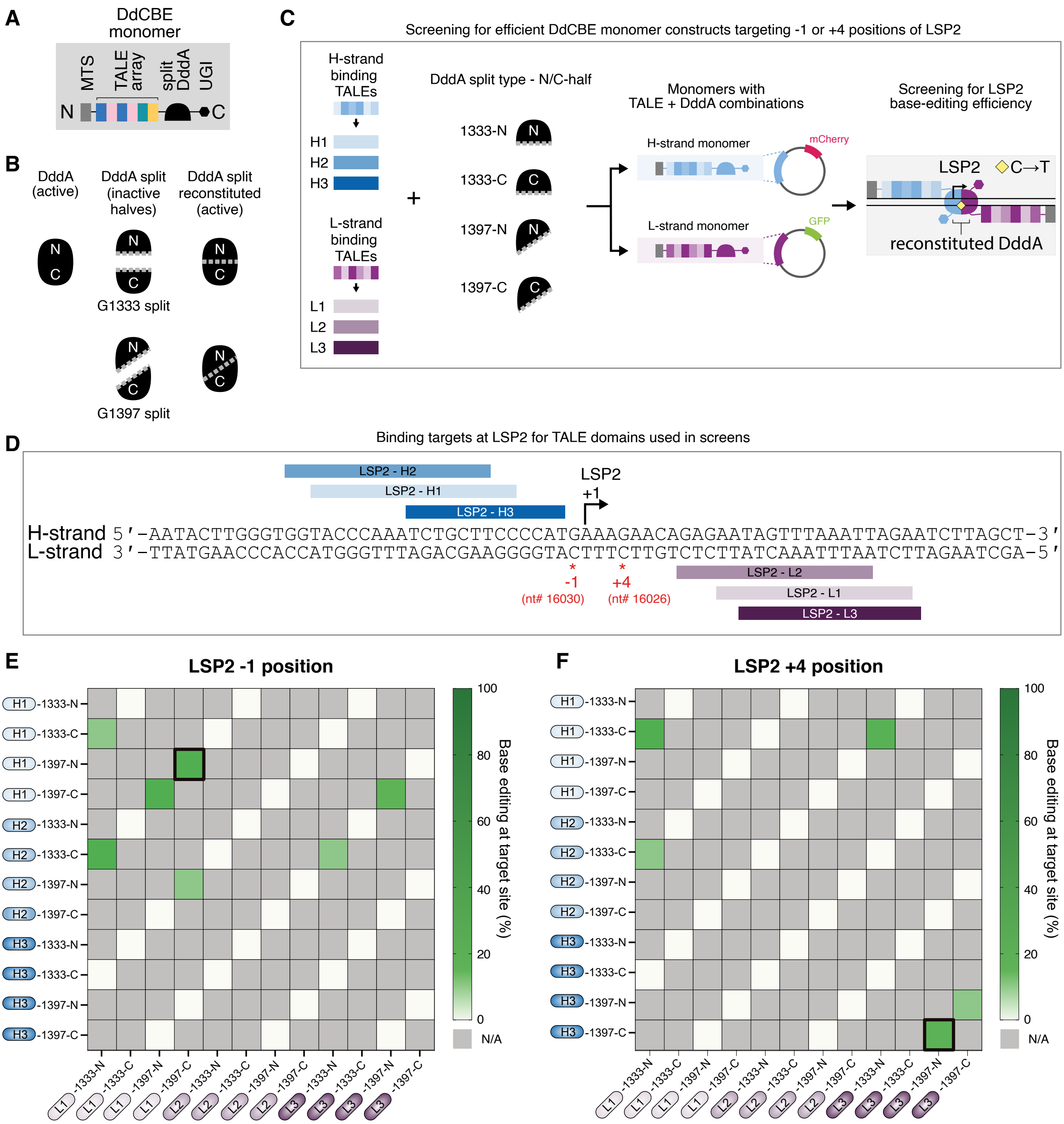

A
DdCBE monomer
MTS
TALE array
split DddA
UGI
N
C
B
DddA (active)
DddA split (inactive halves)
DddA split reconstituted (active)
G1333 split
G1397 split
C
Screening for efficient DdCBE monomer constructs targeting -1 or +4 positions of LSP2
H-strand binding TALEs
H1
H2
H3
L-strand binding TALEs
L1
L2
L3
DddA split type - N/C-half
1333-N
1333-C
1397-N
1397-C
Monomers with TALE + DddA combinations
H-strand monomer
mCherry
L-strand monomer
GFP
Screening for LSP2 base-editing efficiency
LSP2
C→T
reconstituted DddA
D
Binding targets at LSP2 for TALE domains used in screens
LSP2 - H2
LSP2 - H1
LSP2 - H3
LSP2
+1
H-strand 5′-AATACTTGGGTGGTACCCAAATCTGCTTCCCCATGAAAGAACAGAGAATAGTTTAAATTAGAATCTTAGCT-3′
L-strand 3′-TTATGAACCCACCATGGGTTTAGACGAAGGGGTACTTTCTTGTCTCTTATCAAATTTAATCTTAGAATCGA-5′
-1
(nt# 16030)
+4
(nt# 16026)
LSP2 - L2
LSP2 - L1
LSP2 - L3
E
LSP2 -1 position
F
LSP2 +4 position
Base editing at target site (%)
100
80
60
40
20
0
N/A

Figure S6. Design and screening of viable DdCBE constructs for LSP2 base-editing. Related to Figure 7.

(A) Schematic of the DdCBE monomer. Mitochondrial targeting sequence, MTS; transcription activator-like effector array, TALE; split half of the $DddA_{tox}$ cytosine deaminase, DddA; uracil DNA glycosylase inhibitor, UGI; N-terminus of the monomer construct, N; C-terminus of the monomer construct, C. **(B)** Schematic representation of $DddA_{tox}$ split into non-toxic halves at either G1333 or G1397 as described in Mok, *et al*(Mok et al., 2020), which were used in screens. By fusing each half (N or C) to TALE domains that bind to DNA sequences flanking a target site (see right hand side of (C)), both halves are brought together in proximity to a target cytosine base, which reconstitutes the active DddA enzyme for base-editing. **(C)** Modular strategy for the screening of viable L-strand or H-strand targeting DdCBE monomers. Three different TALE domains targeting either L- or H-strand sequences flanking the −1 and +4 residues of LSP2 were combined with N- or C-terminal halves of DddA split at either G1333 or G1397. These were cloned into vectors with distinct fluorescent reporters (mCherry or GFP) to allow successful transfectants to be selected by fluorescence-activated cell sorting (FACS; see **Figure 7A**). The envisaged assembly of DdCBE monomers at LSP2 with the active and reconstituted DddA is also shown. **(D)** Schematic of TALE arrays used in screens designed to target H- and L-strand sequences at the LSP2 locus in human mtDNA. The target cytosine residues (−1C and +4C of LSP2) amenable to DdCBE editing are indicated by red asterisks, along with their coordinates (nt#) on the reference human genome (rCRS). **(E, F)** Quantified heteroplasmy shifts from Sanger sequencing traces of base-edited HEK293T cells, represented as a matrix of DdCBE monomer pair combinations and their base-editing efficiency at (E) −1C or (F) +4C on the L-strand (template strand) of LSP2. A total of 36 combinations of L- and H-strand monomer pairs were tested (white and green boxes in matrices). H- and L-strand binding TALE domains (H1−H3, L1–L3) are coloured according to (D). Thick black squares in screening matrices indicate the chosen DdCBE monomer pairs (H1-1397-N and L1-1397-C targeting LSP2(−1C); H3-1397C and L3-1397-N targeting LSP2(+4C); amino acid sequences listed in **Table S18**.

Figure S7

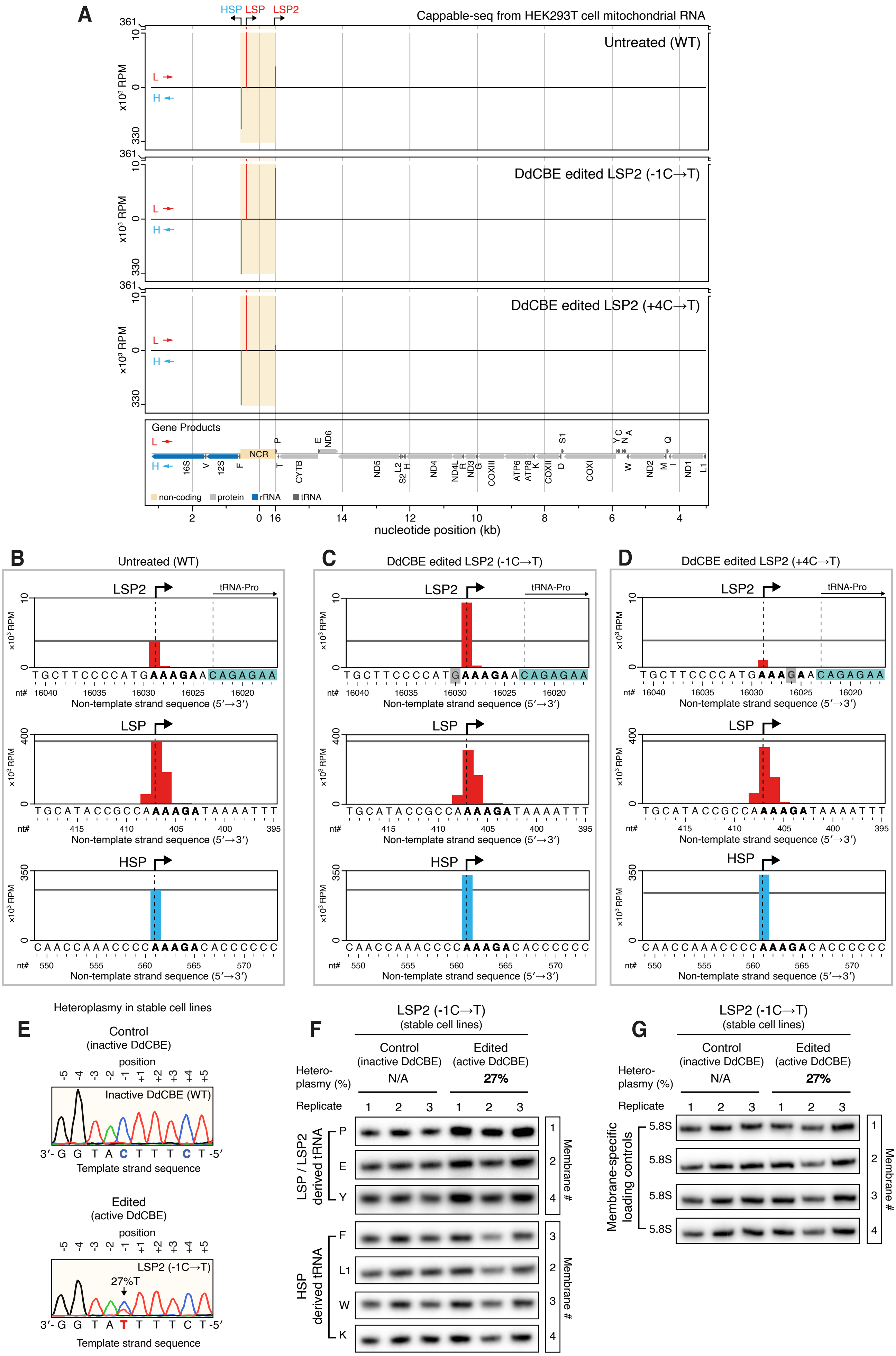
A
HSP LSP LSP2
Cappable-seq from HEK293T cell mitochondrial RNA
Untreated (WT)
DdCBE edited LSP2 (-1C→T)
DdCBE edited LSP2 (+4C→T)
x10³ RPM
L
H
Gene Products
NCR
non-coding protein rRNA tRNA
nucleotide position (kb)
B
Untreated (WT)
C
DdCBE edited LSP2 (-1C→T)
D
DdCBE edited LSP2 (+4C→T)
LSP2
tRNA-Pro
LSP
HSP
Non-template strand sequence (5′→3′)
E
Heteroplasmy in stable cell lines
Control
(inactive DdCBE)
position
Inactive DdCBE (WT)
3′- G G T A C T T T C T -5′
Template strand sequence
Edited
(active DdCBE)
LSP2 (-1C→T)
27%T
3′- G G T A T T T T C T -5′
Template strand sequence
F
LSP2 (-1C→T)
(stable cell lines)
Control
(inactive DdCBE)
Edited
(active DdCBE)
Hetero-
plasmy (%)
N/A
27%
Replicate
LSP / LSP2
derived tRNA
HSP
derived tRNA
Membrane #
G
LSP2 (-1C→T)
(stable cell lines)
Membrane-specific
loading controls
5.8S

Figure S7. Effects on transcription in LSP2-edited cells. Related to Figure 7.

(A) Global Cappable-seq profiles of 5′-triphosphorylated mitochondrial RNA termini (reads per million, RPM) from the L-strand (red peaks) and H-strand (blue peaks), corresponding to TSS sites from mtDNA of control cells (untreated with DdCBEs, 'Untreated (WT)') as well as LSP2-edited ('DdCBE edited LSP2(−1C→T)' and 'DdCBE edited LSP2(+4C→T)') HEK293T cells. 'L' and 'H' labels with arrows indicate the direction of transcription from either strand, and are aligned to L- and H-strand gene products in the lower panel. **(B–D)** Magnified profiles at LSP2, LSP, and HSP loci as in **Figure 7D–L**, expanded to show the absence of additional peaks proximal to the TSS in the cropped view. Horizontal grey lines in (B–D) indicate peak heights from control profiles. **(E)** Representative Sanger sequencing profiles of the LSP2 locus showing heteroplasmies in stable cell lines expressing mtDdCBEs. **(F)** Northern blots of mitochondrial tRNAs as in **Figure 7**, alongside **(G)** the membrane-specific blots of the loading control (5.8S RNA), which was used to quantify Northern blots in **Figure 7O** (e.g. densities of tRNA-Pro (blotted on membrane #1) are paired with densities from 5.8S blots from membrane #1).

The human mitochondrial genome contains a second light strand promoter

Benedict G. Tan[1], Christian D. Mutti[2,5], Yonghong Shi[1,5], Xie Xie[1], Xuefeng Zhu[1], Pedro Silva-Pinheiro[2], Katja E. Menger[3], Héctor Díaz-Maldonado[1], Wei Wei[2,4], Thomas J. Nicholls[3], Patrick F. Chinnery[2,4], Michal Minczuk[2*], Maria Falkenberg[1*], and Claes M. Gustafsson[1,6*]

Affiliations

[1] Department of Medical Biochemistry and Cell Biology, University of Gothenburg; Gothenburg 405 30, Sweden.

[2] Medical Research Council Mitochondrial Biology Unit, University of Cambridge; Cambridge CB2 0XY, UK

[3] Wellcome Centre for Mitochondrial Research, Biosciences Institute, Newcastle University; Newcastle upon Tyne NE2 4HH, UK

[4] Department of Clinical Neurosciences, School of Clinical Medicine, University of Cambridge; Cambridge, UK

[5] These authors contributed equally

[6] Lead Contact

* Corresponding authors: michal.minczuk@mrc-mbu.cam.ac.uk, maria.falkenberg@medkem.gu.se, claes.gustafsson@medkem.gu.se

Supplementary Tables S1–S14

Table S1. PCR templates and primers used to generate *in vitro* transcription templates used in this work. Related to Figures 1–6

The 'T_A' column indicates the annealing temperature used with Phusion DNA Polymerase reactions, where N/A indicates that a 2-step PCR omitting an annealing step was used.

Template Name	Primers		PCR Template	PCR Template type	T_A (°C)	Used in Figure
3′-NCR+HSP series	bt5_aR2	bt6_aR	3′-NCR+HSP	gene strand	N/A	Figure 1, Figure S1
3′-NCR(no HSP)	bt5_bR	bt6_aR	3′-NCR+HSP	gene strand	N/A	Figure 1, Figure S1
LSP+90	bt13_aF	bt13_aR	pEXA128-LSP(HB)	plasmid	65	Figure 2, Fig. S1, Figure 6A,B
HSP+90	bt13_bF	bt13_bR	pEXA128-HSP(HB)	plasmid	65	Figure 2, Figure S1, Figure 6A,B
LSP2+74	bt14_cF	bt14_aR	pEXA128-LSP2(HB)	plasmid	65	Figure 4A, Figure 6A,B
LSP+50 series	bt15_bF	bt15_bR	variable; see Table S10, Table S11 (Mutations)	ssDNA	67	Figure 3
HSP+50 series	bt15_cF	bt15_cR	variable; see Table S8, Table S9 (Mutations)	ssDNA	68	Figure 3
LSP2+50 series	bt15_aF	bt15_aR	variable; see Table S6, Table S7 (Mutations)	ssDNA	67	Figure 3
LSP-FP	bt22_aF	bt22_aR	pEXA128-LSP(HB)	plasmid	68	Figure 4E
HSP-FP	bt22_aF	bt22_bR	pEXA128-HSP(HB)	plasmid	66	Figure 4F
LSP2-FP	bt14_bF	bt14_aR	3′-NCR+HSP	gene strand	68	Figure 4D
F1–F18 series (mtDNA fragments)	Primers and template information listed in Table S8					Figure 2H–J
Primate mitochondrial promoter fragments	Primers and template information listed in Table S9					Figure 5

Table S2. Primers used in Reverse Transcriptase primer extension experiments. Related to Figure 2A,B and Figure S1

Primer sequences can be found in **Table S13**.

Promoter Probed	RNA source	Primer	Used in Figure
LSP	*In vitro* generated RNA from template LSP+90	bt13_aF2	Figure S1B
HSP	*In vitro* generated RNA from template HSP+90	bt13_bR2	Figure S2C
LSP2	*In vitro* generated RNA from template 3′-NCR+HSP(no HSP)	bt9_aF2	Figure 2A/Figure S1A
HSP	Mitochondrial RNA extracted from cell lines	PE-HSP (608-585)	Figure S1E, lanes 4–6
LSP2	Mitochondrial RNA extracted from cell lines	PE-LSP2 (15974-15996)	Figure S1E, lanes 1–3<br>Figure S1F, lanes 1–4

Table S3. Synthetic DNA fragments of 3′-NCR+HSP and of the pEXA128 series. Related to Figure 1 and Figure 4A, and Figure 6A–C.

Construct	Sequence (5′ to 3′ direction)	Details
3′-NCR+HSP	GCTATGACCATGATTACGCCAAGCTT**GTGGTCAAGGGA<br>CCCCTATCTGAGGGGGGTCATCCATGGGGACGAGAAG<br>GGATTTGACTGTAATGTGCTATGTACGGTAAATGGCTTT<br>ATGTACTATGTACTGTTAAGGGTGGGTAGGTTTGTTGG<br>TATCCTAGTGGGTGAGGGGTGGCTTTGGAGTTGCAGTT<br>GATGTGTGATAGTTGAGGGTTGATTGCTGTACTTGCTT<br>GTAAGCATGGGGAGGGGGTTTTGATGTGGATTGGGTTT<br>TTATGTACTACAGGTGGTCAAGTATTTATGGTACCGTAC<br>AATATTCATGGTGGCTGGCAGTAATGTACGAAATACAT<br>AGCGGTTGTTGATGGGTGAGTCAATACTTGGGTGGTAC<br>CCAAATCTGCTTCCCCATGAAAGAACAGAGAATAGTTT<br>AAATTAGAATCTTAGCTTTGGGTGCTAATGGTGGAGTT<br>AAAGACTTTTTCTCTGATT**AGTGTATTGCTTTGAGGAGG<br>TAAGCTACATAAACTGTGGGGGGTGTCTTTGGGGTTTG<br>GTTGGTTCGGGGTATGGGGTTAGCAGCGGTGTGTGTGT<br>GCTGGGTAGGATGGGCGGGGGATCCCCGGGTACCGAG<br>CTCGAATT	Terminal NCR sequence (bold) is nt# 15594–16403. Complementary sequences to pUC19 for Gibson cloning are underlined. Bold text indicates NCR sequences used, with the coordinates indicated accordingly.<br><br>Highlighted sequences indicate the following: cyan, $tRNA^{Pro}$; yellow, HSP fragment (−65 to +50, +1 in red); magenta, TAS-A region; grey, TAS-B region. Mutations to TAS-A and TAS-B indicated in **Table S4**.
pEXA128-LSP(HB)	**AAGCTT**attagtagtatgggagtgggaggggaaaataatgtgttagttgggg<br>gtgactgttaaaagtgcataccgccaaaagataaaatttgaaatctggttaggctgg<br>tgttagggttctttgtttttggggtttggcagagatgtgtttaagtgctgtggccagaa**G<br>GATCC**	The pEXA128 series. Fragments correspond to human mitochondrial promoters inserted into the pEXA128 plasmid used by Eurofins Genomics, in the orientation shown. The +1 nucleotide for each promoter fragment is in red. Promoter fragments contain −73 to +90 of LSP, −75 to +90 of HSP, and −74 to +74 of LSP2 (nt# 318–480, nt# 486–1650, and nt# 15956–16103 of the rCRS, respectively). ‘HB’ and ‘BH’ denote the orientation of HindIII (‘H’) and BamHI (‘B’) restriction sites flanking promoter fragments and are meant to emphasise the inversion of the direction of promoters and thus the direction of transcription. HindIII sites are in bold only, BamHI sites are both in bold and underlined.
pEXA128-HSP(HB)	**AAGCTT**caatacaacccccgcccatcctacccagcacacacacaccgctgct<br>aaccccataccccgaaccaaccaaaccccaaagacacccccacagtttatgta<br>gcttacctcctcaaagcaatacactgaaaatgtttagacgggctcacatcaccccat<br>aaacaaat**GGATCC**	
pEXA128-LSP2(HB)	**AAGCTT**taatgtacgaaatacatagcggttgttgatgggtgagtcaatacttggg<br>tggtacccaaatctgcttccccatgaaagaacagagaatagtttaaattagaatctt<br>agctttgggtgctaatggtggagttaaagactttttctctga**GGATCC**	
pEXA128-LSP(BH)	**GGATCC**ttctggccacagcacttaaacacatctctgccaaaccccaaaaaca<br>aagaaccctaacaccagcctaaccagatttcaaattttatcttttggcggtatgcactt<br>ttaacagtcaccccccaactaacacattattttcccctcccactcccatactactaat<br>**AAGCTT**	
pEXA128-HSP(BH)	**GGATCC**atttgtttatggggtgatgtgagcccgtctaaacattttcagtgtattgcttt<br>gaggaggtaagctacataaactgtgggggtgtctttggggtttggttggttcggggt<br>atggggttagcagcggtgtgtgtgtgctgggtaggatgggcgggggttgtattg**AA<br>GCTT**	
pEXA128-LSP2(BH)	**GGATCC**tcagagaaaaagtctttaactccaccattagcacccaaagctaagat<br>tctaatttaaactattctctgttctttcatggggaagcagatttgggtaccacccaagtat<br>tgactcacccatcaacaaccgctatgtatttcgtacatta**AAGCTT**	

Table S4. Mutations to the TAS-A and TAS-B regions of the 3′-NCR+HSP template (Table S3). Related to Figure 1D.

The sequences of the 20 bp overlapping 'sub-regions' within TAS-A and TAS-B and their respective G↔A/C↔T mutations are indicated. 3′-NCR+HSP constructs containing mutations to each sub-region were ordered as double-stranded synthetic DNA (GeneStrands) from Eurofins Genomics and PCR amplified to generate DNA templates for *in vitro* transcription.

**TAS-A Region**			
**Sub-region**	**Wild-type sequence (5′ to 3′)**	**coordinates on rCRS (nt#)**	**G↔A C↔T mutation (5′ to 3′)**
TAS-A1	CTCACCCATCAACAACCGCT	16067–16086	TCTGTTTGCTGGTGGTTATC
TAS-A2	CAAGTATTGACTCACCCATC	16057–16076	TGGACGCCAGTCTGTTTGCT
TAS-A3	GGGTACCACCCAAGTATTGA	16047–16066	AAACGTTGTTTGGACGCCAG
TAS-A4	AAGCAGATTTGGGTACCACC	16037–16056	GGATGAGCCCAAACGTTGTT
TAS-A5	TTTCATGGGGAAGCAGATTT	16027–16046	CCCTGCAAAAGGATGAGCCC
TAS-A6	TTCTCTGTTCTTTCATGGGG	16017–16036	CCTCTCACCTCCCTGCAAAA
TAS-A7	ATTTAAACTATTCTCTGTTC	16007–16026	GCCCGGGTCGCCTCTCACCT
TAS-A8	TAAGATTCTAATTTAAACTA	15997–16016	CGGAGCCTCGGCCCGGGTCG
TAS-A9	CACCCAAAGCTAAGATTCTA	15987–16006	TGTTTGGGATCGGAGCCTCG
TAS-A10	CCACCATTAGCACCCAAAGC	15977–15996	TTGTTGCCGATGTTTGGGAT
TAS-A11	GTCTTTAACTCCACCATTAG	15967–15986	ACTCCCGGTCTTGTTGCCGA
TAS-A12	CAGAGAAAAAGTCTTTAACT	15957–15976	TGAGAGGGGGACTCCCGGTC
**TAS-B region**			
**Sub-region**	**Wild-type sequence (5′ to 3′)**	**coordinates on rCRS (nt#)**	**G↔A C↔T mutation (5′ to 3′)**
TAS-B1	TACTGCCAGCCACCATGAAT	16102–16121	CGTCATTGATTGTTGCAGGC
TAS-B2	CACCATGAATATTGTACGGT	16112–16131	TGTTGCAGGCGCCACGTAAC
TAS-B3	ATTGTACGGTACCATAAATA	16122–16141	GCCACGTAACGTTGCGGGCG
TAS-B4	ACCATAAATACTTGACCACC	16132–16151	GTTGCGGGCGTCCAGTTGTT
TAS-B5	CTTGACCACCTGTAGTACAT	16142–16161	TCCAGTTGTTCACGACGTGC
TAS-B6	TGTAGTACATAAAAACCCAA	16152–16171	CACGACGTGCGGGGGTTTGG
TAS-B7	AAAAACCCAATCCACATCAA	16162–16181	GGGGGTTTGGCTTGTGCTGG
TAS-B8	TCCACATCAAAACCCCCTCC	16172–16191	CTTGTGCTGGGGTTTTTCTT
TAS-B9	AACCCCCTCCCCATGCTTAC	16182–16201	GGTTTTTCTTTTGCATCCGT
TAS-B10	CCATGCTTACAAGCAAGTAC	16192–16211	TTGCATCCGTGGATGGACGT
TAS-B11	AAGCAAGTACAGCAATCAAC	16202–16221	GGATGGACGTGATGGCTGGT
TAS-B12	AGCAATCAACCCTCAACTAT	16212–16231	GATGGCTGGTTTCTGGTCGC

Table S5. Coordinates of templates F1–F18. Related to Figure 2H.

The 18 Fragments covering the entire annotated rCRS are listed showing the coordinates (nt#) of each fragment. The notation of the fragments (i.e., 1–18) follow the direction of H-strand replication. These fragments were ordered as synthetic linear double-stranded DNA through the GeneStrands service provided by Eurofins Genomics and amplified by PCR (see **Table S1**) to create templates for *in vitro* transcription. PCR primers are listed. The 'T_A' column indicates the annealing temperature used with Phusion DNA Polymerase reactions, where N/A indicates that a 2-step PCR omitting an annealing step was used. Coordinates on the rCRS of mtDNA fragments are indicated.

DNA Template	coordinates (nt#)	Mutations from rCRS	PCR Primers to make linear DNA templates		T_A (°C)	Used in Figure
F1	16134–614	N/A	bt15_F1F	bt15_F1R	N/A	Figure 2H–J
F2	15214–16263	N/A	bt15_F2F	bt15_F2R	N/A	
F3	14294–15343	N/A	bt15_F3F	bt15_F3R	N/A	
F4	13374–14423	N/A	bt15_F4F	bt15_F4R	N/A	
F5	12454–13503	N/A	bt15_F5F	bt15_F5R	N/A	
F6	11534–12583	N/A	bt15_F6F	bt15_F6R	N/A	
F7	10614–11663	N/A	bt15_F7F	bt15_F7R	N/A	
F8	9694–10743	N/A	bt15_F8F	bt15_F8R	N/A	
F9	8774–9823	N/A	bt15_F9F	bt15_F9R	N/A	
F10	7854–8903	N/A	bt15_F10F	bt15_F10R	N/A	
F11	6934–7983	N/A	bt15_F11F	bt15_F11R	N/A	
F12	6014–7063	N/A	bt15_F12F	bt15_F12R	N/A	
F13	5094–6143	N/A	bt15_F13F	bt15_F13R	N/A	
F14	4174–5223	N/A	bt15_F14F	bt15_F14R	N/A	
F15	3254–4303	N/A	bt15_F15F	bt15_F15R	63	
F16	2334–3383	N/A	bt15_F16F	bt15_F16R	N/A	
F17	1414–2463	N/A	bt15_F17F	bt15_F17R	N/A	
F18	494–1543	N/A	bt15_F18F	bt15_F18R	N/A	
F1′ Mut +1/+2	16134–520	nt# 406–407 G↔T/C↔A	bt18_F1Fc	bt15_F1FR	N/A	
F2 Mut +1/+2	15214–16263	nt# 16028–16029 G↔T/C↔A	bt18_F1Fc	bt15_F1FR	N/A	
F18 Mut +1/+2	494–1543	nt# 561–562 G↔T/C↔A	bt15_F2F	bt15_F2R	N/A	
			bt15_F18F	bt15_F18R	N/A	

Table S6. Sequences of ssDNA oligonucleotides used to create 2 bp variants in the LSP2+50 Series. Related to Figure 3.

Sequences of single-stranded DNA used as PCR templates for 2 bp LSP2 promoter mutations in **Figure 3E,F** and referenced in **Table S1**. Mutations are indicated by uppercase lettering.

**LSP2 Promoter mutations**	
**Wild-type LSP2 fragment**	**template strand sequence (5′ to 3′)**
LSP2+50 TS WT	ccattagcacccaaagctaagattctaatttaaactattctctgttctttcatggggaagcagatttgggtaccacccaagtattgactcacccatcaac
**G↔A/C↔T Mutations**	**template strand sequence (5′ to 3′)**
LSP2+50 TSmutA+10+9	ccattagcacccaaagctaagattctaatttaaactattcCTtgttctttcatggggaagcagatttgggtaccacccaagtattgactcacccatcaac
LSP2+50 TSmutA+8+7	ccattagcacccaaagctaagattctaatttaaactattctcCAttctttcatggggaagcagatttgggtaccacccaagtattgactcacccatcaac
LSP2+50 TSmutA+6+5	ccattagcacccaaagctaagattctaatttaaactattctctgCCctttcatggggaagcagatttgggtaccacccaagtattgactcacccatcaac
LSP2+50 TSmutA+4+3	ccattagcacccaaagctaagattctaatttaaactattctctgttTCttcatggggaagcagatttgggtaccacccaagtattgactcacccatcaac
LSP2+50 TSmutA+2+1	ccattagcacccaaagctaagattctaatttaaactattctctgttctCCcatggggaagcagatttgggtaccacccaagtattgactcacccatcaac
LSP2+50 TSmutA-1-2	ccattagcacccaaagctaagattctaatttaaactattctctgttctttTGtggggaagcagatttgggtaccacccaagtattgactcacccatcaac
LSP2+50 TSmutA-3-4	ccattagcacccaaagctaagattctaatttaaactattctctgttctttcaCAgggaagcagatttgggtaccacccaagtattgactcacccatcaac
LSP2+50 TSmutA-5-6	ccattagcacccaaagctaagattctaatttaaactattctctgttctttcatgAAgaagcagatttgggtaccacccaagtattgactcacccatcaac
LSP2+50 TSmutA-7-8	ccattagcacccaaagctaagattctaatttaaactattctctgttctttcatgggAGagcagatttgggtaccacccaagtattgactcacccatcaac
LSP2+50 TSmutA-9-10	ccattagcacccaaagctaagattctaatttaaactattctctgttctttcatggggaGAcagatttgggtaccacccaagtattgactcacccatcaac
LSP2+50 TSmutA-11-12	ccattagcacccaaagctaagattctaatttaaactattctctgttctttcatggggaagTGgatttgggtaccacccaagtattgactcacccatcaac
LSP2+50 TSmutA-13-14	ccattagcacccaaagctaagattctaatttaaactattctctgttctttcatggggaagcaAGtttgggtaccacccaagtattgactcacccatcaac
LSP2+50 TSmutA-15-16	ccattagcacccaaagctaagattctaatttaaactattctctgttctttcatggggaagcagaCCtgggtaccacccaagtattgactcacccatcaac
LSP2+50 TSmutA-17-18	ccattagcacccaaagctaagattctaatttaaactattctctgttctttcatggggaagcagattCAggtaccacccaagtattgactcacccatcaac
LSP2+50 TSmutA-19-20	ccattagcacccaaagctaagattctaatttaaactattctctgttctttcatggggaagcagatttgAAtaccacccaagtattgactcacccatcaac
**G↔T/C↔A Mutations**	**template strand sequence (5′ to 3′)**
LSP2+50 TSmutB+10+9	ccattagcacccaaagctaagattctaatttaaactattcGAtgttctttcatggggaagcagatttgggtaccacccaagtattgactcacccatcaac
LSP2+50 TSmutB+8+7	ccattagcacccaaagctaagattctaatttaaactattctcGTttctttcatggggaagcagatttgggtaccacccaagtattgactcacccatcaac
LSP2+50 TSmutB+6+5	ccattagcacccaaagctaagattctaatttaaactattctctgGGctttcatggggaagcagatttgggtaccacccaagtattgactcacccatcaac
LSP2+50 TSmutB+4+3	ccattagcacccaaagctaagattctaatttaaactattctctgttAGttcatggggaagcagatttgggtaccacccaagtattgactcacccatcaac
LSP2+50 TSmutB+2+1	ccattagcacccaaagctaagattctaatttaaactattctctgttctGGcatggggaagcagatttgggtaccacccaagtattgactcacccatcaac
LSP2+50 TSmutB-1-2	ccattagcacccaaagctaagattctaatttaaactattctctgttctttACtggggaagcagatttgggtaccacccaagtattgactcacccatcaac
LSP2+50 TSmutB-3-4	ccattagcacccaaagctaagattctaatttaaactattctctgttctttcaGTgggaagcagatttgggtaccacccaagtattgactcacccatcaac
LSP2+50 TSmutB-5-6	ccattagcacccaaagctaagattctaatttaaactattctctgttctttcatgTTgaagcagatttgggtaccacccaagtattgactcacccatcaac
LSP2+50 TSmutB-7-8	ccattagcacccaaagctaagattctaatttaaactattctctgttctttcatgggTCagcagatttgggtaccacccaagtattgactcacccatcaac
LSP2+50 TSmutB-9-10	ccattagcacccaaagctaagattctaatttaaactattctctgttctttcatggggaCTcagatttgggtaccacccaagtattgactcacccatcaac
LSP2+50 TSmutB-11-12	ccattagcacccaaagctaagattctaatttaaactattctctgttctttcatggggaagACgatttgggtaccacccaagtattgactcacccatcaac
LSP2+50 TSmutB-13-14	ccattagcacccaaagctaagattctaatttaaactattctctgttctttcatggggaagcaTCtttgggtaccacccaagtattgactcacccatcaac
LSP2+50 TSmutB-15-16	ccattagcacccaaagctaagattctaatttaaactattctctgttctttcatggggaagcagaGGtgggtaccacccaagtattgactcacccatcaac
LSP2+50 TSmutB-17-18	ccattagcacccaaagctaagattctaatttaaactattctctgttctttcatggggaagcagattGTggtaccacccaagtattgactcacccatcaac
LSP2+50 TSmutB-19-20	ccattagcacccaaagctaagattctaatttaaactattctctgttctttcatggggaagcagatttgTTtaccacccaagtattgactcacccatcaac

Table S7. Sequences of ssDNA oligonucleotides used to create 1 bp variants in the LSP2+50 Series. Related to Figure 5, Figure S2 and Figure 7.

Sequences of single-stranded DNA used as PCR templates for 1 bp LSP2 promoter mutations in **Figure 7B** and **Figure S2D,G** and referenced in **Table S1**. Mutations are indicated by uppercase lettering.

**LSP2 Promoter mutations**	
**G↔A/C↔T Mutations**	**template strand sequence (5′ to 3′)**
LSP2_TSmutA+6	ccattagcacccaaagctaagattctaatttaaactattctctgCtctttcatggggaagcagatttgggtaccacccaagtattgactcacccatcaac
LSP2_TSmutA+5	ccattagcacccaaagctaagattctaatttaaactattctctgtCctttcatggggaagcagatttgggtaccacccaagtattgactcacccatcaac
LSP2_TSmutA+4	ccattagcacccaaagctaagattctaatttaaactattctctgttTtttcatggggaagcagatttgggtaccacccaagtattgactcacccatcaac
LSP2_TSmutA+3	ccattagcacccaaagctaagattctaatttaaactattctctgttcCttcatggggaagcagatttgggtaccacccaagtattgactcacccatcaac
LSP2_TSmutA+2	ccattagcacccaaagctaagattctaatttaaactattctctgttctCtcatggggaagcagatttgggtaccacccaagtattgactcacccatcaac
LSP2_TSmutA+1	ccattagcacccaaagctaagattctaatttaaactattctctgttcttCcatggggaagcagatttgggtaccacccaagtattgactcacccatcaac
LSP2_TSmutA-1	ccattagcacccaaagctaagattctaatttaaactattctctgttctttTatggggaagcagatttgggtaccacccaagtattgactcacccatcaac
LSP2_TSmutA-2	ccattagcacccaaagctaagattctaatttaaactattctctgttctttcGtggggaagcagatttgggtaccacccaagtattgactcacccatcaac
LSP2_TSmutA-3	ccattagcacccaaagctaagattctaatttaaactattctctgttctttcaCgggaagcagatttgggtaccacccaagtattgactcacccatcaac
LSP2_TSmutA-4	ccattagcacccaaagctaagattctaatttaaactattctctgttctttcatAgggaagcagatttgggtaccacccaagtattgactcacccatcaac
LSP2_TSmutA-5	ccattagcacccaaagctaagattctaatttaaactattctctgttctttcatgAggaagcagatttgggtaccacccaagtattgactcacccatcaac
**G↔T/C↔A Mutations**	**template strand sequence (5′ to 3′)**
LSP2_TSmutB+6	ccattagcacccaaagctaagattctaatttaaactattctctgGtctttcatggggaagcagatttgggtaccacccaagtattgactcacccatcaac
LSP2_TSmutB+5	ccattagcacccaaagctaagattctaatttaaactattctctgtGctttcatggggaagcagatttgggtaccacccaagtattgactcacccatcaac
LSP2_TSmutB+4	ccattagcacccaaagctaagattctaatttaaactattctctgttAtttcatggggaagcagatttgggtaccacccaagtattgactcacccatcaac
LSP2_TSmutB+3	ccattagcacccaaagctaagattctaatttaaactattctctgttcGttcatggggaagcagatttgggtaccacccaagtattgactcacccatcaac
LSP2_TSmutB+2	ccattagcacccaaagctaagattctaatttaaactattctctgttctGtcatggggaagcagatttgggtaccacccaagtattgactcacccatcaac
LSP2_TSmutB+1	ccattagcacccaaagctaagattctaatttaaactattctctgttcttGcatggggaagcagatttgggtaccacccaagtattgactcacccatcaac
LSP2_TSmutB-1	ccattagcacccaaagctaagattctaatttaaactattctctgttctttAatggggaagcagatttgggtaccacccaagtattgactcacccatcaac
LSP2_TSmutB-2	ccattagcacccaaagctaagattctaatttaaactattctctgttctttcCtggggaagcagatttgggtaccacccaagtattgactcacccatcaac
LSP2_TSmutB-3	ccattagcacccaaagctaagattctaatttaaactattctctgttctttcaGgggaagcagatttgggtaccacccaagtattgactcacccatcaac
LSP2_TSmutB-4	ccattagcacccaaagctaagattctaatttaaactattctctgttctttcatTgggaagcagatttgggtaccacccaagtattgactcacccatcaac
LSP2_TSmutB-5	ccattagcacccaaagctaagattctaatttaaactattctctgttctttcatgTggaagcagatttgggtaccacccaagtattgactcacccatcaac

Table S8. Sequences of ssDNA oligonucleotides used to create 2 bp variants in the LSP+50 Series. Related to Figure 3.

Sequences of single-stranded DNA used as PCR templates for 2 bp LSP promoter mutations in **Figure 3E,F** and referenced in **Table S1**. Mutations are indicated by uppercase lettering.

**LSP Promoter mutations**	
**Wild-type LSP fragment**	**template strand sequence (5′ to 3′)**
LSP+50 TS WT	aaaacaaagaaccctaacaccagcctaaccagatttcaaattttatcttttggcggtatgcacttttaacagtcaccccccaactaacacattattttcc
**G↔A/C↔T Mutations**	**template strand sequence (5′ to 3′)**
LSP+50 TSmutA+10+9	aaaacaaagaaccctaacaccagcctaaccagatttcaaaCCttatcttttggcggtatgcacttttaacagtcaccccccaactaacacattattttcc
LSP+50 TSmutA+8+7	aaaacaaagaaccctaacaccagcctaaccagatttcaaattCCatcttttggcggtatgcacttttaacagtcaccccccaactaacacattattttcc
LSP+50 TSmutA+6+5	aaaacaaagaaccctaacaccagcctaaccagatttcaaattttGCcttttggcggtatgcacttttaacagtcaccccccaactaacacattattttcc
LSP+50 TSmutA+4+3	aaaacaaagaaccctaacaccagcctaaccagatttcaaattttatTCtttggcggtatgcacttttaacagtcaccccccaactaacacattattttcc
LSP+50 TSmutA+2+1	aaaacaaagaaccctaacaccagcctaaccagatttcaaattttatctCCtggcggtatgcacttttaacagtcaccccccaactaacacattattttcc
LSP+50 TSmutA-1-2	aaaacaaagaaccctaacaccagcctaaccagatttcaaattttatctttCAgcggtatgcacttttaacagtcaccccccaactaacacattattttcc
LSP+50 TSmutA-3-4	aaaacaaagaaccctaacaccagcctaaccagatttcaaattttatcttttgATggtatgcacttttaacagtcaccccccaactaacacattattttcc
LSP+50 TSmutA-5-6	aaaacaaagaaccctaacaccagcctaaccagatttcaaattttatcttttggcAAtatgcacttttaacagtcaccccccaactaacacattattttcc
LSP+50 TSmutA-7-8	aaaacaaagaaccctaacaccagcctaaccagatttcaaattttatcttttggcggCGtgcacttttaacagtcaccccccaactaacacattattttcc
LSP+50 TSmutA-9-10	aaaacaaagaaccctaacaccagcctaaccagatttcaaattttatcttttggcggtaCAcacttttaacagtcaccccccaactaacacattattttcc
LSP+50 TSmutA-11-12	aaaacaaagaaccctaacaccagcctaaccagatttcaaattttatcttttggcggtatgTGcttttaacagtcaccccccaactaacacattattttcc
LSP+50 TSmutA-13-14	aaaacaaagaaccctaacaccagcctaaccagatttcaaattttatcttttggcggtatgcaTCtttaacagtcaccccccaactaacacattattttcc
LSP+50 TSmutA-15-16	aaaacaaagaaccctaacaccagcctaaccagatttcaaattttatcttttggcggtatgcactCCtaacagtcaccccccaactaacacattattttcc
LSP+50 TSmutA-17-18	aaaacaaagaaccctaacaccagcctaaccagatttcaaattttatcttttggcggtatgcactttCGacagtcaccccccaactaacacattattttcc
LSP+50 TSmutA-19-20	aaaacaaagaaccctaacaccagcctaaccagatttcaaattttatcttttggcggtatgcacttttaGTagtcaccccccaactaacacattattttcc
**G↔T/C↔A Mutations**	**template strand sequence (5′ to 3′)**
LSP+50 TSmutB+10+9	aaaacaaagaaccctaacaccagcctaaccagatttcaaaGGttatcttttggcggtatgcacttttaacagtcaccccccaactaacacattattttcc
LSP+50 TSmutB+8+7	aaaacaaagaaccctaacaccagcctaaccagatttcaaattGGatcttttggcggtatgcacttttaacagtcaccccccaactaacacattattttcc
LSP+50 TSmutB+6+5	aaaacaaagaaccctaacaccagcctaaccagatttcaaattttCGcttttggcggtatgcacttttaacagtcaccccccaactaacacattattttcc
LSP+50 TSmutB+4+3	aaaacaaagaaccctaacaccagcctaaccagatttcaaattttatAGtttggcggtatgcacttttaacagtcaccccccaactaacacattattttcc
LSP+50 TSmutB+2+1	aaaacaaagaaccctaacaccagcctaaccagatttcaaattttatctGGtggcggtatgcacttttaacagtcaccccccaactaacacattattttcc
LSP+50 TSmutB-1-2	aaaacaaagaaccctaacaccagcctaaccagatttcaaattttatctttGTgcggtatgcacttttaacagtcaccccccaactaacacattattttcc
LSP+50 TSmutB-3-4	aaaacaaagaaccctaacaccagcctaaccagatttcaaattttatcttttgTAggtatgcacttttaacagtcaccccccaactaacacattattttcc
LSP+50 TSmutB-5-6	aaaacaaagaaccctaacaccagcctaaccagatttcaaattttatcttttggcTTtatgcacttttaacagtcaccccccaactaacacattattttcc
LSP+50 TSmutB-7-8	aaaacaaagaaccctaacaccagcctaaccagatttcaaattttatcttttggcggGCtgcacttttaacagtcaccccccaactaacacattattttcc
LSP+50 TSmutB-9-10	aaaacaaagaaccctaacaccagcctaaccagatttcaaattttatcttttggcggtaGTcacttttaacagtcaccccccaactaacacattattttcc
LSP+50 TSmutB-11-12	aaaacaaagaaccctaacaccagcctaaccagatttcaaattttatcttttggcggtatgACcttttaacagtcaccccccaactaacacattattttcc
LSP+50 TSmutB-13-14	aaaacaaagaaccctaacaccagcctaaccagatttcaaattttatcttttggcggtatgcaAGtttaacagtcaccccccaactaacacattattttcc
LSP+50 TSmutB-15-16	aaaacaaagaaccctaacaccagcctaaccagatttcaaattttatcttttggcggtatgcactGGtaacagtcaccccccaactaacacattattttcc
LSP+50 TSmutB-17-18	aaaacaaagaaccctaacaccagcctaaccagatttcaaattttatcttttggcggtatgcactttGCacagtcaccccccaactaacacattattttcc
LSP+50 TSmutB-19-20	aaaacaaagaaccctaacaccagcctaaccagatttcaaattttatcttttggcggtatgcacttttaCAagtcaccccccaactaacacattattttcc

Table S9. Sequences of ssDNA oligonucleotides used to create 1 bp variants in the LSP+50 Series. Related to Figure 5 and Figure S2.

Sequences of single-stranded DNA used as PCR templates for 1 bp LSP promoter mutations in **Figure S2E,H** and referenced in **Table S1**. Mutations are indicated by uppercase lettering.

LSP Promoter mutations	
**G↔A/C↔T Mutations**	**template strand sequence (5′ to 3′)**
LSP_TSmutA+6	aaaacaaagaaccctaacaccagcctaaccagatttcaaattttGtcttttggcggtatgcacttttaacagtcaccccccaactaacacattattttcc
LSP_TSmutA+5	aaaacaaagaaccctaacaccagcctaaccagatttcaaattttaCcttttggcggtatgcacttttaacagtcaccccccaactaacacattattttcc
LSP_TSmutA+4	aaaacaaagaaccctaacaccagcctaaccagatttcaaattttatTttttggcggtatgcacttttaacagtcaccccccaactaacacattattttcc
LSP_TSmutA+3	aaaacaaagaaccctaacaccagcctaaccagatttcaaattttatcCtttggcggtatgcacttttaacagtcaccccccaactaacacattattttcc
LSP_TSmutA+2	aaaacaaagaaccctaacaccagcctaaccagatttcaaattttatctCttggcggtatgcacttttaacagtcaccccccaactaacacattattttcc
LSP_TSmutA+1	aaaacaaagaaccctaacaccagcctaaccagatttcaaattttatcttCtggcggtatgcacttttaacagtcaccccccaactaacacattattttcc
LSP_TSmutA-1	aaaacaaagaaccctaacaccagcctaaccagatttcaaattttatctttCggcggtatgcacttttaacagtcaccccccaactaacacattattttcc
LSP_TSmutA-2	aaaacaaagaaccctaacaccagcctaaccagatttcaaattttatcttttAgcggtatgcacttttaacagtcaccccccaactaacacattattttcc
LSP_TSmutA-3	aaaacaaagaaccctaacaccagcctaaccagatttcaaattttatcttttgAcggtatgcacttttaacagtcaccccccaactaacacattattttcc
LSP_TSmutA-4	aaaacaaagaaccctaacaccagcctaaccagatttcaaattttatcttttggTggtatgcacttttaacagtcaccccccaactaacacattattttcc
LSP_TSmutA-5	aaaacaaagaaccctaacaccagcctaaccagatttcaaattttatcttttggcAgtatgcacttttaacagtcaccccccaactaacacattattttcc
**G↔T/C↔A Mutations**	**template strand sequence (5′ to 3′)**
LSP_TSmutB+6	aaaacaaagaaccctaacaccagcctaaccagatttcaaattttCtcttttggcggtatgcacttttaacagtcaccccccaactaacacattattttcc
LSP_TSmutB+5	aaaacaaagaaccctaacaccagcctaaccagatttcaaattttaGcttttggcggtatgcacttttaacagtcaccccccaactaacacattattttcc
LSP_TSmutB+4	aaaacaaagaaccctaacaccagcctaaccagatttcaaattttatAttttggcggtatgcacttttaacagtcaccccccaactaacacattattttcc
LSP_TSmutB+3	aaaacaaagaaccctaacaccagcctaaccagatttcaaattttatcGtttggcggtatgcacttttaacagtcaccccccaactaacacattattttcc
LSP_TSmutB+2	aaaacaaagaaccctaacaccagcctaaccagatttcaaattttatctGttggcggtatgcacttttaacagtcaccccccaactaacacattattttcc
LSP_TSmutB+1	aaaacaaagaaccctaacaccagcctaaccagatttcaaattttatcttGtggcggtatgcacttttaacagtcaccccccaactaacacattattttcc
LSP_TSmutB-1	aaaacaaagaaccctaacaccagcctaaccagatttcaaattttatctttGggcggtatgcacttttaacagtcaccccccaactaacacattattttcc
LSP_TSmutB-2	aaaacaaagaaccctaacaccagcctaaccagatttcaaattttatcttttTgcggtatgcacttttaacagtcaccccccaactaacacattattttcc
LSP_TSmutB-3	aaaacaaagaaccctaacaccagcctaaccagatttcaaattttatcttttgTcggtatgcacttttaacagtcaccccccaactaacacattattttcc
LSP_TSmutB-4	aaaacaaagaaccctaacaccagcctaaccagatttcaaattttatcttttggAggtatgcacttttaacagtcaccccccaactaacacattattttcc
LSP_TSmutB-5	aaaacaaagaaccctaacaccagcctaaccagatttcaaattttatcttttggcTgtatgcacttttaacagtcaccccccaactaacacattattttcc

Table S10. Sequences of ssDNA oligonucleotides used to create 2 bp variants in the HSP+50 Series. Related to Figure 3.

Sequences of single-stranded DNA used as PCR templates for 2 bp HSP promoter mutations in **Figure 3E,F** and referenced in **Table S1**. Mutations are indicated by uppercase lettering.

**HSP Promoter mutations**	
**Wild-type HSP fragment**	**template strand sequence (5′ to 3′)**
HSP+50 TS WT	agtgtattgctttgaggaggtaagctacataaactgtggggggtgtctttggggtttggttggttcggggtatggggttagcagcggtgtgtgtgctg
**G↔A/C↔T Mutations**	**template strand sequence (5′ to 3′)**
HSP+50 TSmutA+10+9	agtgtattgctttgaggaggtaagctacataaactgtgggAAgtgtctttggggtttggttggttcggggtatggggttagcagcggtgtgtgtgctg
HSP+50 TSmutA+8+7	agtgtattgctttgaggaggtaagctacataaactgtggggACgtctttggggtttggttggttcggggtatggggttagcagcggtgtgtgtgctg
HSP+50 TSmutA+6+5	agtgtattgctttgaggaggtaagctacataaactgtggggggtACctttggggtttggttggttcggggtatggggttagcagcggtgtgtgtgctg
HSP+50 TSmutA+4+3	agtgtattgctttgaggaggtaagctacataaactgtggggggtgtTCttggggtttggttggttcggggtatggggttagcagcggtgtgtgtgctg
HSP+50 TSmutA+2+1	agtgtattgctttgaggaggtaagctacataaactgtggggggtgtctCCggggtttggttggttcggggtatggggttagcagcggtgtgtgtgctg
HSP+50 TSmutA-1-2	agtgtattgctttgaggaggtaagctacataaactgtggggggtgtctttAAggtttggttggttcggggtatggggttagcagcggtgtgtgtgctg
HSP+50 TSmutA-3-4	agtgtattgctttgaggaggtaagctacataaactgtggggggtgtctttggAAtttggttggttcggggtatggggttagcagcggtgtgtgtgctg
HSP+50 TSmutA-5-6	agtgtattgctttgaggaggtaagctacataaactgtggggggtgtctttggggCCtggttggttcggggtatggggttagcagcggtgtgtgtgctg
HSP+50 TSmutA-7-8	agtgtattgctttgaggaggtaagctacataaactgtggggggtgtctttggggttCAgttggttcggggtatggggttagcagcggtgtgtgtgctg
HSP+50 TSmutA-9-10	agtgtattgctttgaggaggtaagctacataaactgtggggggtgtctttggggtttgACtggttcggggtatggggttagcagcggtgtgtgtgctg
HSP+50 TSmutA-11-12	agtgtattgctttgaggaggtaagctacataaactgtggggggtgtctttggggtttggtCAgttcggggtatggggttagcagcggtgtgtgtgctg
HSP+50 TSmutA-13-14	agtgtattgctttgaggaggtaagctacataaactgtggggggtgtctttggggtttggttgACtcggggtatggggttagcagcggtgtgtgtgctg
HSP+50 TSmutA-15-16	agtgtattgctttgaggaggtaagctacataaactgtggggggtgtctttggggtttggttggtCTggggtatggggttagcagcggtgtgtgtgctg
HSP+50 TSmutA-17-18	agtgtattgctttgaggaggtaagctacataaactgtggggggtgtctttggggtttggttggttcAAggtatggggttagcagcggtgtgtgtgctg
HSP+50 TSmutA-19-20	agtgtattgctttgaggaggtaagctacataaactgtggggggtgtctttggggtttggttggttcggAAtatggggttagcagcggtgtgtgtgctg
**G↔T/C↔A Mutations**	**template strand sequence (5′ to 3′)**
HSP+50 TSmutB+10+9	agtgtattgctttgaggaggtaagctacataaactgtgggTTgtgtctttggggtttggttggttcggggtatggggttagcagcggtgtgtgtgctg
HSP+50 TSmutB+8+7	agtgtattgctttgaggaggtaagctacataaactgtggggTGgtctttggggtttggttggttcggggtatggggttagcagcggtgtgtgtgctg
HSP+50 TSmutB+6+5	agtgtattgctttgaggaggtaagctacataaactgtggggggtTGctttggggtttggttggttcggggtatggggttagcagcggtgtgtgtgctg
HSP+50 TSmutB+4+3	agtgtattgctttgaggaggtaagctacataaactgtggggggtgtAGttggggtttggttggttcggggtatggggttagcagcggtgtgtgtgctg
HSP+50 TSmutB+2+1	agtgtattgctttgaggaggtaagctacataaactgtggggggtgtctGGggggtttggttggttcggggtatggggttagcagcggtgtgtgtgtgctg
HSP+50 TSmutB-1-2	agtgtattgctttgaggaggtaagctacataaactgtggggggtgtctttTTggtttggttggttcggggtatggggttagcagcggtgtgtgtgctg
HSP+50 TSmutB-3-4	agtgtattgctttgaggaggtaagctacataaactgtggggggtgtctttggTTtttggttggttcggggtatggggttagcagcggtgtgtgtgctg
HSP+50 TSmutB-5-6	agtgtattgctttgaggaggtaagctacataaactgtggggggtgtctttggggGGtggttggttcggggtatggggttagcagcggtgtgtgtgtgctg
HSP+50 TSmutB-7-8	agtgtattgctttgaggaggtaagctacataaactgtggggggtgtctttggggttGTgttggttcggggtatggggttagcagcggtgtgtgtgctg
HSP+50 TSmutB-9-10	agtgtattgctttgaggaggtaagctacataaactgtggggggtgtctttggggtttgTGtggttcggggtatggggttagcagcggtgtgtgtgctg
HSP+50 TSmutB-11-12	agtgtattgctttgaggaggtaagctacataaactgtggggggtgtctttggggtttggtGTgttcggggtatggggttagcagcggtgtgtgtgctg
HSP+50 TSmutB-13-14	agtgtattgctttgaggaggtaagctacataaactgtggggggtgtctttggggtttggttgTGtcggggtatggggttagcagcggtgtgtgtgctg
HSP+50 TSmutB-15-16	agtgtattgctttgaggaggtaagctacataaactgtggggggtgtctttggggtttggttggtGAggggtatggggttagcagcggtgtgtgtgctg
HSP+50 TSmutB-17-18	agtgtattgctttgaggaggtaagctacataaactgtggggggtgtctttggggtttggttggttcTTggtatggggttagcagcggtgtgtgtgctg
HSP+50 TSmutB-19-20	agtgtattgctttgaggaggtaagctacataaactgtggggggtgtctttggggtttggttggttcggTTtatggggttagcagcggtgtgtgtgctg

Table S11. Sequences of ssDNA oligonucleotides used to create 1 bp variants in the HSP+50 Series. Related to Figure 5 and Figure S2.

Sequences of single-stranded DNA used as PCR templates for 1 bp HSP promoter mutations in **Figure S2F,I** and referenced in **Table S1**. Mutations are indicated by uppercase lettering.

**HSP Promoter mutations**	
**G↔A/C↔T Mutations**	**template strand sequence (5′ to 3′)**
HSP_TSmutA+6	agtgtattgctttgaggaggtaagctacataaactgtggggggtAtctttggggtttggttggttcggggtatggggttagcagcggtgtgtgtgtgctg
HSP_TSmutA+5	agtgtattgctttgaggaggtaagctacataaactgtggggggtgCctttggggtttggttggttcggggtatggggttagcagcggtgtgtgtgtgctg
HSP_TSmutA+4	agtgtattgctttgaggaggtaagctacataaactgtggggggtgtTtttggggtttggttggttcggggtatggggttagcagcggtgtgtgtgtgctg
HSP_TSmutA+3	agtgtattgctttgaggaggtaagctacataaactgtggggggtgtcCttggggtttggttggttcggggtatggggttagcagcggtgtgtgtgtgctg
HSP_TSmutA+2	agtgtattgctttgaggaggtaagctacataaactgtggggggtgtctCtggggtttggttggttcggggtatggggttagcagcggtgtgtgtgtgctg
HSP_TSmutA+1	agtgtattgctttgaggaggtaagctacataaactgtggggggtgtcttCgggtttggttggttcggggtatggggttagcagcggtgtgtgtgtgctg
HSP_TSmutA-1	agtgtattgctttgaggaggtaagctacataaactgtggggggtgtctttAgggtttggttggttcggggtatggggttagcagcggtgtgtgtgtgctg
HSP_TSmutA-2	agtgtattgctttgaggaggtaagctacataaactgtggggggtgtctttgAggtttggttggttcggggtatggggttagcagcggtgtgtgtgtgctg
HSP_TSmutA-3	agtgtattgctttgaggaggtaagctacataaactgtggggggtgtctttggAgtttggttggttcggggtatggggttagcagcggtgtgtgtgtgctg
HSP_TSmutA-4	agtgtattgctttgaggaggtaagctacataaactgtggggggtgtctttgggAtttggttggttcggggtatggggttagcagcggtgtgtgtgtgctg
HSP_TSmutA-5	agtgtattgctttgaggaggtaagctacataaactgtggggggtgtctttggggCttggttggttcggggtatggggttagcagcggtgtgtgtgtgctg
**G↔T/C↔A Mutations**	**template strand sequence (5′ to 3′)**
HSP_TSmutB+6	agtgtattgctttgaggaggtaagctacataaactgtggggggtTtctttggggtttggttggttcggggtatggggttagcagcggtgtgtgtgtgctg
HSP_TSmutB+5	agtgtattgctttgaggaggtaagctacataaactgtggggggtgGctttggggtttggttggttcggggtatggggttagcagcggtgtgtgtgtgctg
HSP_TSmutB+4	agtgtattgctttgaggaggtaagctacataaactgtggggggtgtAtttggggtttggttggttcggggtatggggttagcagcggtgtgtgtgtgctg
HSP_TSmutB+3	agtgtattgctttgaggaggtaagctacataaactgtggggggtgtcGttggggtttggttggttcggggtatggggttagcagcggtgtgtgtgtgctg
HSP_TSmutB+2	agtgtattgctttgaggaggtaagctacataaactgtggggggtgtctGtggggtttggttggttcggggtatggggttagcagcggtgtgtgtgtgctg
HSP_TSmutB+1	agtgtattgctttgaggaggtaagctacataaactgtggggggtgtcttGgggtttggttggttcggggtatggggttagcagcggtgtgtgtgtgctg
HSP_TSmutB-1	agtgtattgctttgaggaggtaagctacataaactgtggggggtgtctttTgggtttggttggttcggggtatggggttagcagcggtgtgtgtgtgctg
HSP_TSmutB-2	agtgtattgctttgaggaggtaagctacataaactgtggggggtgtctttgTggtttggttggttcggggtatggggttagcagcggtgtgtgtgtgctg
HSP_TSmutB-3	agtgtattgctttgaggaggtaagctacataaactgtggggggtgtctttggTgtttggttggttcggggtatggggttagcagcggtgtgtgtgtgctg
HSP_TSmutB-4	agtgtattgctttgaggaggtaagctacataaactgtggggggtgtctttgggTtttggttggttcggggtatggggttagcagcggtgtgtgtgtgctg
HSP_TSmutB-5	agtgtattgctttgaggaggtaagctacataaactgtggggggtgtctttggggGttggttggttcggggtatggggttagcagcggtgtgtgtgtgctg

Table S12. Sequences of ssDNA oligonucleotides used as PCR templates for Primate mitochondrial promoter experiments. Related to Figure 5.

Primate mtDNA fragments were ordered as single-stranded DNA oligonucleotides and amplified by PCR with Phusion DNA Polymerase to create DNA templates for *in vitro* transcription (see **Table S1**). LSP and HSP fragments used an annealing temperature of 65 °C, and LSP2 fragments were made with 2-step PCR omitting an annealing step. The coordinates indicated refer to GenBank annotated genomes: Human (*Homo sapiens,* NC_012920), Neanderthal (*H.s. neanderthalensis* AM948965), Chimpanzee (*Pan troglodytes,* NC_001643), Gorilla (*Gorilla gorilla,* NC_001645), Sumatran Orangutan (*Pongo abelii,* NC_002083), Lar Gibbon (*Hylobates lar,* NC_002082).

	Primate Promoter Fragment	Coordinates (nt)	template strand sequence (5′ to 3′)	Primers	
Primate LSP fragments	lsp_human	358–457	used template "LSP+50 TS WT" (see **Table S8**)	bt15_bF	bt15_bR
	lsp_neanderthal	358–457	aaaacaaagaaccctaacaccagcctaaccagatttcaaattttatcttttggcggtatacacttttaacagtcaccccctaactaacacattattttcc	neanLSPF	neanLSPR
	lsp_chimpanzee	16338–16437	aaaacaaagaacccagacgccagcctagccagacttcaaatttcatctttaggcggtatgcacttttaacagtcacccctcaattaacatgccctccccc	chimpLSPF	chimpLSPR
	lsp_gorilla	16178–16277	cccccccccgcactagaaaacagccccaccaaactccaaatttcatcttttggcggtatgcacttttaacagtcacccctcaactaacatagtcagccca	gorillaLSPF	gorillaLSPR
	lsp_orangutan	16285–16384	ctctcaacaaccctaccccatcaacccaacaaaatccaatttttatctttaggctatgtgcactttcaacaggcacccctcaactaacacaatctccttc	orangLSPF	orangLSPR
	lsp_gibbon	16305–16404	tttgccaaaccccaaaaacaaagtaaccccagtgagccagacccatcttttggcggtacacgcctttaacagccaccccctcaactaacacatatttttt	gibbonLSPF	gibbonLSPR
Primate HSP fragments	hsp_human	497–616	attttcagtgtattgctttgaggaggtaagctacataaactgtgggggtgtctttggggtttggttggttcggggtatggggttagcagcggtgtgtgtgctgggtaggatgggcgg	humanHSPF	humanHSPR
	hsp_neanderthal	493–612	attttcagtgtattgctttgaggaggtaagctacataaactgtgggggtgtctttggggtttggttggctcggggtatggggttagcagcggtgtgtgctgggtaggatgggcgggggt	neanHSPF	neanHSPR
	hsp_chimpanzee	16475–40	attttcagtgtattgctttgagggggtaagctacataaactgtgtaggggtgtctttggggtttggttggttcagggtatggggttagcagcggtatatgtgttgagtagggtgagtagg	chimpHSPF	chimpHSPR
	hsp_gorilla	16285–40	attttcagtgtattgctttggggaggtaagctacataaactgtgtggggtgtctttggggtttggttaattcggggtataggatcagcagcagtgtgtgttgctagggcgggcgggggtt	gorillaHSPF	gorillaHSPR
	hsp_orangutan	16420–40	ttttcagtgcattgctttggatagaataagctacataaaccgtgcggggtgtcttttgggttgggttggtctggggtgtgggggtagtagcggtgtttgtggggaaaatggagaaagagg	orangHSPF	orangHSPR
	hsp_gibbon	16393–40	attttcagtgttttgctttgggtagttaagctacataaacggggactctttggggataggctggtttgaggtaagggagtagtagtaggtgggagggaaaagaagaaaaaaaaaatatgtg	gibbonHSPF	gibbonHSPR
Primate LSP fragments	lsp2_human	16004–16123	ctaatttaaactattctctgttctttcatggggaagcagatttgggtaccacccaagtattgactcacccatcaacaaccgctatgtatttcgtacattactgccagccaccatgaatat	humanLSP2F	humanLSP2R
	lsp2_neanderthal	15999–16118	ctaatttaaactattctctgttctttcatgggggagcagatttgggtaccacccaagtattgactcacccatcagcaaccgctatgtatttcgtacattactgccagccaccatgaatat	neanLSP2F	neanLSP2R
	lsp2_chimpanzee	15422–15541	ctaatttaaactattctctgttctttcatggggaagcaaatttaggtaccacctaagtactggctcattcattacaaccgctatgtatttcgtacattactgccagccaccatgaatatc	chimpLSP2F	chimpLSP2R
	lsp2_gorilla	15427–15546	ctaatttaaactattctctgttctttcatggggagacaaatttgggtactaccagtattagctaacccatcataattatcatgtatgtcgtgcattactgccagacaccatgaataatgt	gorillaLSP2F	gorillaLSP2R
	lsp2_orangutan	15464–15583	taattttaaactactctctgttctttcatgggggaccagatttgggtgccaccccagtactgacccatttctaacggcctatgtatttcgtacattcctgctagccaacatgaatatcac	orangLSP2F	orangLSP2R
	lsp2_gibbon	15424–15543	ctaacttaaactattctctgtattctcatgtggaagccattttgggtacaaccccagtactaacccacttctccacaactctatgtacttcgtacattactgccagtccccatgcatatt	gibbonLSP2F	gibbonLSP2R

Table S13. List of Primers and Probes used in this study. Related to STAR Methods

Oligo Name	Sequence (5′ to 3′)
bt5_aR2	ATGCGCGGATCCCCCGCCCATCCTACCCAGCACACAC
bt5_aF2	GACCATAAGCTTTGGGAGTGGGAGGGGAAAATAATG
bt5_bR	CCCACAGTTTATGTAGCTTACCTCCTCAAAGCAAT
bt5_bF	TTTGAAATCTGGTTAGGCTGGTGTTAGGGTTCTTT
bt6_aR	TACGCCAAGCTTGTGGTCAAGGGACCCCTA
bt9_aF2	CTCCACCATTAGCACCCAAAGC
bt10_R	GACGTAGGATCCTTTACCATCATTGGACAAGTAGCATC
bt13_aF	TTCTGGCCACAGCACTTAAACACATCTCTG
bt13_aF2	CCCCAAAAACAAAGAACCCTAACAC
bt13_bF	CAATACAACCCCCGCCCATCCTAC
bt13_aR	ATTAGTAGTATGGGAGTGGGAGGGG
bt13_bR	ATTTGTTTATGGGGTGATGTGAGCCCG
bt13_bR2	TTTTCAGTGTATTGCTTTGAGGAGGT
bt14_aR	TCAGAGAAAAAGTCTTTAACTCCACCATTAGCAC
bt14_cF	TAATGTACGAAATACATAGCGGTTGTTGATG
bt14_bF	AGTTGAGGGTTGATTGCTGTACTTGCTTG
bt22_aF	ACAAGCCCGTCAGGGCGCGTC
bt22_aR	TAAACACATCTCTGCCAAACCCCAAAAACAAAG
bt22_bR	ATGTGAGCCCGTCTAAACATTTTCAGTGTATTGC
bt15_aF	GTTGATGGGTGAGTCAATACTTGGGTGG
bt15_bF	GGAAAATAATGTGTTAGTTGGGGGGTGAC
bt15_cF	CAGCACACACACACCGCTGCTAACCCC
bt15_aR	CCATTAGCACCCAAAGCTAAGATTCTAATTTAAAC
bt15_bR	AAAACAAAGAACCCTAACACCAGCCTAACC
bt15_cR	AGTGTATTGCTTTGAGGAGGTAAGCTACATAAAC
bt15_F1F	TTTCAGTGTATTGCTTTGAGGAGGTAAGCTAC
bt15_F1R	CATAAATACTTGACCACCTGTAGTACATAAAAACCC
bt15_F2F	AGGGGTGGCTTTGGAGTTGCAGTTGATG
bt15_F2R	TGGGACAGACCTAGTTCAATGAATCTGAGG
bt15_F3F	GAATAGGAGGTGGAGTGTTGCTAGGGC
bt15_F3R	TAAATTATTCAGCTTCCTACACTATTAAAGTTTAC
bt15_F4F	CAGGGGTTGAGGTCTTGGTGAGTGTTTTAG
bt15_F4R	CATCCACAACCTTAACAATGAACAAGATATTCG
bt15_F5F	GAAACCTGTGAGGAAAGGTATTCCTGCTAATG
bt15_F5R	GTCGCATCCACCTTTATTATCAGTCTCTTCCCC
bt15_F6F	CTAGTTTGAAGCTTAGGGAGAGCTGGGTTG
bt15_F6R	TACCCCTTCCTTGTACTATCCCTATGAGGC
bt15_F7F	GAATGGCTGTTACTACGAGGGCTATGTGG
bt15_F7R	CTCAACACCCACTCCCTCTTAGCCAATATTG
bt15_F8F	TATGTACGTAGTCTAGGCCATATGTGTTGGAG
bt15_F8R	TGCTTATTACAATTTTACTGGGTCTCTATTTTACCC
bt15_F9F	AGTCCGTGGAAGCCTGTGGCTAC
bt15_F9R	ACCTCCTCGGACTCCTGCCTCACTC
bt15_F10F	GCTAGGGCATTTTTAATCTTAGAGCGAAAGCC
bt15_F10R	TCAACGATCCCTCCCTTACCATCAAATCAATTGG
bt15_F11F	AGGTCGCCTGGTTCTAGGAATAATGGGGG
bt15_F11R	TCATCTTTCTTTTCACCGTAGGTGGCCTGACTGG

Table S13 (continued).

Oligo Name	Sequence (5′ to 3′)
bt15_F12F	AATACAGCTCCTATTGATAGGACATAGTGGAAG
bt15_F12R	TCGAGCCGAGCTGGGCCAGCC
bt15_F13F	GTTGCCAAAGCCTCCGATTATGATGGG
bt15_F13R	ATTATCCTAACTACTACCGCATTCCTACTACTC
bt15_F14F	CTAGGGAGAGGAGGGTGGATGGAATTAAG
bt15_F14R	TGAAAAAACTTCCTACCACTCACCCTAGCATTAC
bt15_F15F	ATTTACTCTATCAAAGTAACTCTTTTATCAGACATATTTC
bt15_F15R	CGCATAAAACTTAAAACTTTACAGTCAGAGGTTC
bt15_F16F	TTTCGTTCGGTAAGCATTAGGAATGCCATTGC
bt15_F16R	CATAAGCCTGCGTCAGATTAAAACACTGAACTG
bt15_F17F	TTTTTTTAACCTTTCCTTATGAGCATGCCTGTGTTG
bt15_F17R	CGAAGGTGGATTTAGCAGTAAACTAAGAGTAG
bt15_F18F	AAATGCGTAGGGGTTTTAGTTAAATGTCCTTTGAAG
bt15_F18R	CCCCCGCCCATCCTACCCAGC
bt18_F1Fc	GTGTGTGCTGGGTAGGATGG
bt22_aF	ACAAGCCCGTCAGGGCGCGTC
bt22_aR	TAAACACATCTCTGCCAAACCCCAAAAACAAAG
bt22_bR	ATGTGAGCCCGTCTAAACATTTTCAGTGTATTGC
PE-HSP(608-585)	TGTATTGCTTTGAGGAGGTAAGCT
PE-LSP2(15974-15996)	ACTCCACCATTAGCACCCAAAGC
LSP2_Fw	GGGCCTGTCCTTGTAGTATAAAC
LSP2_Rv	GCATGGGGAGGGGGTTTTGATG
5.8SRNA_probe	TCATCGACGCACGAGCCGAGTGATC
bt32_tRNAPro	GAGAAAAAGTCTTTAACTCCACCATTAGCAC
bt32_tRNAGlu	CTCGCACGGACTACAACCACGACCAATGATATG
bt32_tRNATyr	GGCCTAACCCCTGTCTTTAGATTTACAGTCCAATG
tRNAPhe_probe	CTAAACATTTTCAGTGTATTGC
tRNALeu1_probe	GTTTTATGCGATTACCGGGC
tRNATrp_probe	CAAAGCCCTCAGTAAGTTGCAA
tRNALys_probe	AAAGGTTAATGCTAAGTTAGC

Table S14. Amino acid sequences of DdCBE pairs used to edit LSP2. Related to Figure S6E,F and STAR Methods.

DdCBE pairs targeting LSP2 (−1C) made use of H1-1397-N and L1-1397-C, and DdCBE pairs targeting LSP2(+4C) made use of H3-1397C and L3-1397-N. The asterisk (*) indicates a stop codon.

**DdCBE monomer**	**Amino acid sequence (1-letter code)**
H1-G1397-N<br>(H-strand DdCBE monomer)	MALSRAVCGTSRQLAPVLGYLGSRQKHSLPDYPYDVPDYAGYPYDVPDYAGYPYDVPDYAMDI<br>ADLRTLGYSQQQQEKIKPKVRSTVAQHHEALVGHGFTHAHIVALSQHPAALGTVAVKYQDMIAA<br>LPEATHEAIVGVGKQWSGARALEALLTVAGELRGPPLQLDTGQLLKIAKRGGVTAVEAVHAWR<br>NALTGAPLNLTPQQVVAIASNIGGKQALETVQRLLPVLCQAHGLTPDQVVAIASHDGGKQALETV<br>QRLLPVLCQAHGLTPAQVVAIASHDGGKQALETVQRLLPVLCQAHGLTPAQVVAIASHDGGKQA<br>LETVQRLLPVLCQAHGLTPAQVVAIASNIGGKQALETVQRLLPVLCQAHGLTPDQVVAIASNIGG<br>KQALETVQRLLPVLCQAHGLTPDQVVAIASNIGGKQALETVQRLLPVLCQAHGLTPDQVVAIASN<br>GGGKQALETVQRLLPVLCQAHGLTPEQVVAIASHDGGKQALETVQRLLPVLCQAHGLTPAQVV<br>AIASNGGGKQALETVQRLLPVLCQAHGLTPEQVVAIASNNGGKQALETVQRLLPVLCQAHGLTP<br>EQVVAIASHDGGKQALETVQRLLPVLCQAHGLTPAQVVAIASNGGGKQALETVQRLLPVLCQAH<br>GLTPEQVVAIASNGGGKQALETVQRLLPVLCQAHGLTPEQVVAIASHDGGKQALETVQRLLPVL<br>CQAHGLTPAQVVAIASHDGGRPALESIVAQLSRPDPALAALTNDHLVALACLGGRPALDAVKKG<br>LGGSGSYALGPYQISAPQLPAYNGQTVGTFYYVNDAGGLESKVFSSGGPTPYPNYANAGHVE<br>GQSALFMRDNGISEGLVFHNNPEGTCGFCVNMTETLLPENAKMTVVPPEGSGGSTNLSDIIEKE<br>TGKQLVIQESILMLPEEVEEVIGNKPESDILVHTAYDESTDENVMLLTSDAPEYKPWALVIQDSNG<br>ENKIKML*
L1-G1397-C<br>(L-strand DdCBE monomer)	MASVLTPLLLRGLTGSARRLPVPRAKIHSLDYKDHDGDYKDHDIDYKDDDDKMDIADLRTLGYS<br>QQQQEKIKPKVRSTVAQHHEALVGHGFTHAHIVALSQHPAALGTVAVKYQDMIAALPEATHEAIV<br>GVGKQWSGARALEALLTVAGELRGPPLQLDTGQLLKIAKRGGVTAVEAVHAWRNALTGAPLNL<br>TPQQVVAIASHDGGKQALETVQRLLPVLCQAHGLTPAQVVAIASNGGGKQALETVQRLLPVLCQ<br>AHGLTPEQVVAIASNIGGKQALETVQRLLPVLCQAHGLTPDQVVAIASNIGGKQALETVQRLLPV<br>LCQAHGLTPDQVVAIASNGGGKQALETVQRLLPVLCQAHGLTPEQVVAIASNGGGKQALETVQ<br>RLLPVLCQAHGLTPEQVVAIASNGGGKQALETVQRLLPVLCQAHGLTPEQVVAIASNIGGKQAL<br>ETVQRLLPVLCQAHGLTPDQVVAIASNIGGKQALETVQRLLPVLCQAHGLTPDQVVAIASNIGGK<br>QALETVQRLLPVLCQAHGLTPDQVVAIASHDGGKQALETVQRLLPVLCQAHGLTPAQVVAIASN<br>GGGKQALETVQRLLPVLCQAHGLTPEQVVAIASNIGGKQALETVQRLLPVLCQAHGLTPDQVVA<br>IASNGGGKQALETVQRLLPVLCQAHGLTPEQVVAIASNGGGRPALESIVAQLSRPDPALAALTN<br>DHLVALACLGGRPALDAVKKGLGGSAIPVKRGATGETKVFTGNSNSPKSPTKGGCSGGSTNLS<br>DIIEKETGKQLVIQESILMLPEEVEEVIGNKPESDILVHTAYDESTDENVMLLTSDAPEYKPWALVI<br>QDSNGENKIKML*
H3-G1397-C<br>(H-strand DdCBE monomer)	MALSRAVCGTSRQLAPVLGYLGSRQKHSLPDYPYDVPDYAGYPYDVPDYAGYPYDVPDYAMDI<br>ADLRTLGYSQQQQEKIKPKVRSTVAQHHEALVGHGFTHAHIVALSQHPAALGTVAVKYQDMIAA<br>LPEATHEAIVGVGKQWSGARALEALLTVAGELRGPPLQLDTGQLLKIAKRGGVTAVEAVHAWR<br>NALTGAPLNLTPQQVVAIASHDGGKQALETVQRLLPVLCQAHGLTPAQVVAIASNGGGKQALET<br>VQRLLPVLCQAHGLTPEQVVAIASNNGGKQALETVQRLLPVLCQAHGLTPEQVVAIASHDGGKQ<br>ALETVQRLLPVLCQAHGLTPAQVVAIASNGGGKQALETVQRLLPVLCQAHGLTPEQVVAIASNG<br>GGKQALETVQRLLPVLCQAHGLTPEQVVAIASHDGGKQALETVQRLLPVLCQAHGLTPAQVVAI<br>ASHDGGKQALETVQRLLPVLCQAHGLTPAQVVAIASHDGGKQALETVQRLLPVLCQAHGLTPA<br>QVVAIASHDGGKQALETVQRLLPVLCQAHGLTPAQVVAIASNIGGKQALETVQRLLPVLCQAHG<br>LTPDQVVAIASNGGGRPALESIVAQLSRPDPALAALTNDHLVALACLGGRPALDAVKKGLGGSAI<br>PVKRGATGETKVFTGNSNSPKSPTKGGCSGGSTNLSDIIEKETGKQLVIQESILMLPEEVEEVIG<br>NKPESDILVHTAYDESTDENVMLLTSDAPEYKPWALVIQDSNGENKIKML*
L3-G1397-N<br>(L-strand DdCBE monomer)	MASVLTPLLLRGLTGSARRLPVPRAKIHSLDYKDHDGDYKDHDIDYKDDDDKMDIADLRTLGYS<br>QQQQEKIKPKVRSTVAQHHEALVGHGFTHAHIVALSQHPAALGTVAVKYQDMIAALPEATHEAIV<br>GVGKQWSGARALEALLTVAGELRGPPLQLDTGQLLKIAKRGGVTAVEAVHAWRNALTGAPLNL<br>TPQQVVAIASNGGGKQALETVQRLLPVLCQAHGLTPEQVVAIASHDGGKQALETVQRLLPVLCQ<br>AHGLTPAQVVAIASNGGGKQALETVQRLLPVLCQAHGLTPEQVVAIASNIGGKQALETVQRLLP<br>VLCQAHGLTPDQVVAIASNIGGKQALETVQRLLPVLCQAHGLTPDQVVAIASNGGGKQALETVQ<br>RLLPVLCQAHGLTPEQVVAIASNGGGKQALETVQRLLPVLCQAHGLTPEQVVAIASNGGGKQAL<br>ETVQRLLPVLCQAHGLTPEQVVAIASNIGGKQALETVQRLLPVLCQAHGLTPDQVVAIASNIGGK<br>QALETVQRLLPVLCQAHGLTPDQVVAIASNIGGKQALETVQRLLPVLCQAHGLTPDQVVAIASHD<br>GGKQALETVQRLLPVLCQAHGLTPAQVVAIASNGGGKQALETVQRLLPVLCQAHGLTPEQVVAI<br>ASNIGGRPALESIVAQLSRPDPALAALTNDHLVALACLGGRPALDAVKKGLGGSGSYALGPYQIS<br>APQLPAYNGQTVGTFYYVNDAGGLESKVFSSGGPTPYPNYANAGHVEGQSALFMRDNGISEG<br>LVFHNNPEGTCGFCVNMTETLLPENAKMTVVPPEGSGGSTNLSDIIEKETGKQLVIQESILMLPE<br>EVEEVIGNKPESDILVHTAYDESTDENVMLLTSDAPEYKPWALVIQDSNGENKIKML*
